# Targeting Menin disrupts the KMT2A/B and polycomb balance to paradoxically activate bivalent genes

**DOI:** 10.1038/s41556-022-01056-x

**Published:** 2023-01-12

**Authors:** Christina E. Sparbier, Andrea Gillespie, Juliana Gomez, Nishi Kumari, Ali Motazedian, Kah Lok Chan, Charles C. Bell, Omer Gilan, Yih-Chih Chan, Sarah Popp, Daniel J. Gough, Melanie A. Eckersley-Maslin, Sarah-Jane Dawson, Paul J. Lehner, Kate D. Sutherland, Patricia Ernst, Gerard M. McGeehan, Enid Y. N. Lam, Marian L. Burr, Mark A. Dawson

**Affiliations:** 1 Cancer Research Division, Peter MacCallum Cancer Centre, Melbourne, Victoria 3000, Australia; 2 Sir Peter MacCallum Department of Oncology, University of Melbourne, Victoria 3052, Australia; 3 The John Curtin School of Medical Research, The Australian National University, ACT 2601, Australia; 4 Department of Clinical Haematology, Peter MacCallum Cancer Centre & Royal Melbourne Hospital, Melbourne, Victoria 3000, Australia; 5 Australian Centre for Blood Diseases, Monash University, Melbourne, VIC 3004, Australia; 6 Department of Molecular Translational Science, Faculty of Medicine, Nursing and Health Sciences, Monash University, Clayton, Victoria, 3800, Australia; 7 Centre for Cancer Research, Hudson Institute of Medical Research, Clayton, Victoria 3168, Australia; 8 Department of Anatomy and Physiology, University of Melbourne, Melbourne, Victoria 3000, Australia; 9 Centre for Cancer Research, University of Melbourne, Melbourne, Victoria 3000, Australia; 10 Cambridge Institute of Therapeutic Immunology and Infectious Disease, Jeffrey Cheah Biomedical Centre, Cambridge Biomedical Campus, Cambridge, UK; 11 ACRF Cancer Biology and Stem Cells Division, Walter and Eliza Hall Institute of Medical Research, Parkville, Victoria 3052, Australia; 12 Department of Medical Biology, The University of Melbourne, Parkville, Victoria 3010, Australia; 13 Department of Pediatrics, Section of Hematology/Oncology/Bone Marrow Transplant, University of Colorado/Anschutz Medical Campus, Aurora, CO, USA; 14 Pharmacology, University of Colorado/Anschutz Medical Campus, Aurora, CO, USA; 15 Syndax Pharmaceuticals, Waltham, MA 02451, USA; 16 Department of Anatomical Pathology, ACT Pathology, Canberra Health Services, Australia

## Abstract

Precise control of activating H3K4me3 and repressive H3K27me3 histone modifications at bivalent promoters is essential for normal development and is frequently corrupted in cancer. By coupling a cell surface readout of bivalent MHC class I gene expression with whole genome CRISPR/Cas9 screens, we identify specific roles for MTF2-PRC2.1, PCGF1-PRC1.1 and Menin-KMT2A/B complexes in maintaining bivalency. Unexpectedly, genetic loss or pharmacological inhibition of Menin phenocopies the effects of polycomb disruption, resulting in derepression of bivalent genes in both cancer cells and pluripotent stem cells. Whilst Menin and KMT2A/B contribute to H3K4me3 at active genes, a separate Menin-independent function of KMT2A/B maintains H3K4me3 and opposes polycomb-mediated repression at bivalent genes. Release of KMT2A from active genes following Menin targeting alters the balance of polycomb and KMT2A at bivalent genes, facilitating gene activation. This functional partitioning of Menin-KMT2A/B complex components reveals therapeutic opportunities that can be leveraged through inhibition of Menin.

## Introduction

Critical genes primed for tissue-specific expression may be concurrently marked by activating H3K4me3 and repressive H3K27me3 histone modifications, respectively catalysed by trithorax- and polycomb-group proteins^1–6^. These ‘bivalent’ chromatin domains maintain genes in a transcriptionally inert state, poised for subsequent activation or stable repression^7–9^. Polycomb repressive complex 2 (PRC2) catalyses methylation of histone H3 lysine 27 and contains a core complex composed of EED, SUZ12 and either EZH1 or EZH2^10–12^. Accessory proteins associate with SUZ12 to define two main PRC2 subcomplexes; PRC2.1 contains a polycomb-like protein (MTF2, PHF1 or PHF19) plus EPOP or PALI1/2, while PRC2.2 contains JARID2 and AEBP2^6,13–16^. Polycomb repressive complex 1 (PRC1) contains core RING1A or RING1B ubiquitin ligase subunits that dimerise with a PCGF protein to ubiquitinate histone H2A lysine 119 (H2AK119Ub). Whilst canonical PRC1 complexes can mediate chromatin compaction^17,18^, most H2AK119Ub is catalysed by variant PRC1 complexes containing RYBP or YAF2 and either PCGF1/3/5 or 6^19–21^.

Conversely, KMT2 (SET1/MLL) histone methyltransferases catalyse methylation of histone H3 lysine 4, which is associated with transcriptional activation. In mammals, six different KMT2 methyltransferases form distinct multiprotein complexes^4^. Menin (*MEN1*) is unique to KMT2A (MLL1) or KMT2B (MLL2) containing complexes, which catalyse H3K4me3 at the promoters of developmental genes^22,23^. The frequency of mutations or dysregulation of polycomb and trithorax proteins in cancer^4,24,25^ highlights the fundamental importance of the polycomb-trithorax axis for normal cellular differentiation and has prompted substantial investment in developing targeted inhibitors of these complexes^26^. Characterising the mechanisms regulating bivalency is therefore vital to understand how these processes become corrupted in cancer and identify opportunities for therapeutic intervention.

## Results

### Genome-scale CRISPR screens identify regulators of bivalency

**Extended Data Fig. 1:**
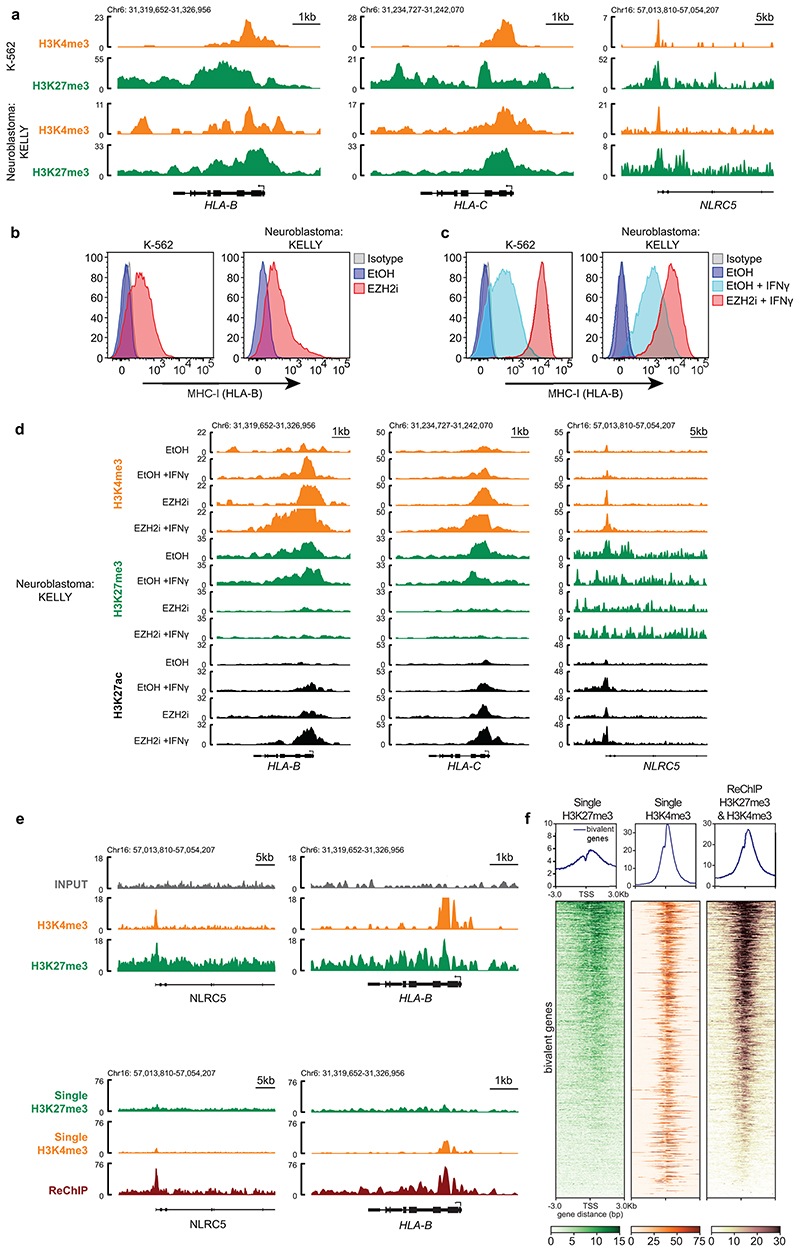
MHC-I genes harbour bivalent H3K4me3 and H3K27me3 modifications. (a) Genomic snapshots of MHC-I genes showing H3K4me3 and H3K27me3 CUT&Tag in K-562 and ChIPseq in Neuroblastoma KELLY cell lines. The K-562 tracks are also shown in the control cells in Fig. 2h and H3K27me3 control cells in Fig. 6f. (b & c) Cell surface MHC-I in K-562 (left) and KELLY (right) cells following treatment with EPZ-011989 and (c) ± 10ng/mL IFN-*γ* (48h K-562, 24h KELLY). (d) Genomic snapshots of MHC-I genes showing ChIP-seq for H3K4me3, H3K27me3 and H3K27ac in KELLY cells treated with EtOH (control) or EPZ-011989 ± IFN-*γ*. (e & f) ChIP re-ChIP-seq of single H3K27me3, single H3K4me3 and reChIP (H3K27me3 and H3K4me3) in K-562 cells. (e) Genomic snapshots of bivalent MHC-I genes. (f) Heatmaps show bivalent genes -3kb TSS/ +3kb TES, with genomic regions ordered by H3K27me3 read density in the single H3K27me3 ChIP sample. (b/c) show representative plots from 3 experiments (Supplementary Figure 3).

MHC-I gene promoters are bivalently modified during development, which can be exploited in cancer cells to silence MHC-I expression and evade CD8^+^ T-cells^27^. In cells exhibiting bivalent modification of MHC-I genes, low levels of cell surface MHC-I can be induced either by PRC2 inhibition or by exposing cells to interferon-gamma (IFN-*γ*), which induces expression of IRF1 and the MHC-I specific coactivator NLRC5 (Extended Data Fig. 1a/b/c). Induction of high MHC-I levels requires IFN-*γ* treatment in conjunction with inhibition of polycomb to establish a permissive chromatin state (Extended Data Fig. 1c/d). This dynamic system is amenable for high-throughput screening to study how chromatin modifiers and transcription factors induced by external stimuli resolve bivalency.

**Extended Data Fig. 2:**
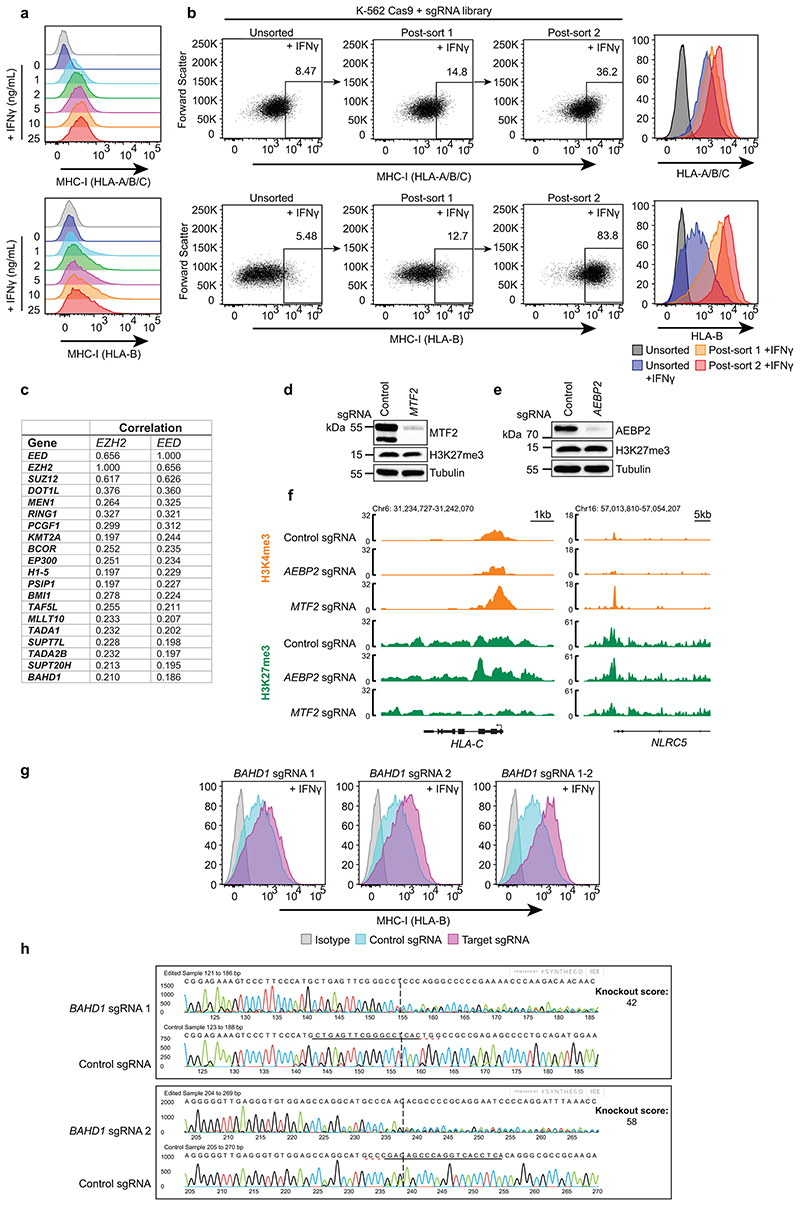
Genome wide CRISPR/Cas9 screen identifies regulators of MHC Class I expression. (a) Cell surface MHC-I, pan-HLA-A,B,C (top panel) and HLA-B (bottom) specific antibodies, in K-562 Cas9 cells treated with the indicated IFN-*γ* doses for 24h. (b) K-562 cells stably expressing Cas9 were mutagenised by infection with a pooled lentiviral sgRNA library and treated with 1ng/mL IFN-*γ* for 24h prior to FACS sorting. Rare MHC-I high cells were enriched by 2 successive rounds of FACS sorting for mCherry positive (containing sgRNA vector) MHC-I positive cells. FACS dot plots and histograms show MHC-I expression in unsorted, post sort 1 and post sort 2 in K-562 Cas9 cells transduced with the CRIPSR sgRNA library and sorted with either pan-HLA-A,B,C (top panels) or HLA-B (bottom panels) specific antibodies. (c) Table depicting correlation between CRISPR gene effect scores (Fig. 1e) for top 20 shared *EZH2* and *EED* co-dependent genes calculated from combined CRISPR survival screens in 990 cancer cell lines in Cancer Dependency Map (httns://denman.org/nortal/)^31, 32^ Table indicates Pearson correlation coefficients. (d & e) Immunoblots of K-562 Cas9 cells transduced with control and (d) *MTF2* or (e) *AEBP2* sgRNA. (f) H3K4me3 and H3K27me3 CUT&Tag. Genomic snapshots of bivalent MHC-I genes in K-562 cells transduced with control, *MTF2* and *AEBP2* sgRNA. The H3K4me3 control tracks are the same control tracks in Fig. 7c. (g) Cell surface MHC-I in K-562 Cas9 cells transduced with control or *BAHD1-specific* sgRNAs and treated with 10ng/mL IFN-*γ* for 48h. Representative plots from 3 experiments (Supplementary Figure 3). (h) Knockout scores of individual sgRNA targeting *BAHD1* measured using Synthego Performance Analysis, Interference of CRISPR editing (ICE) Analysis.

**Figure 1:**
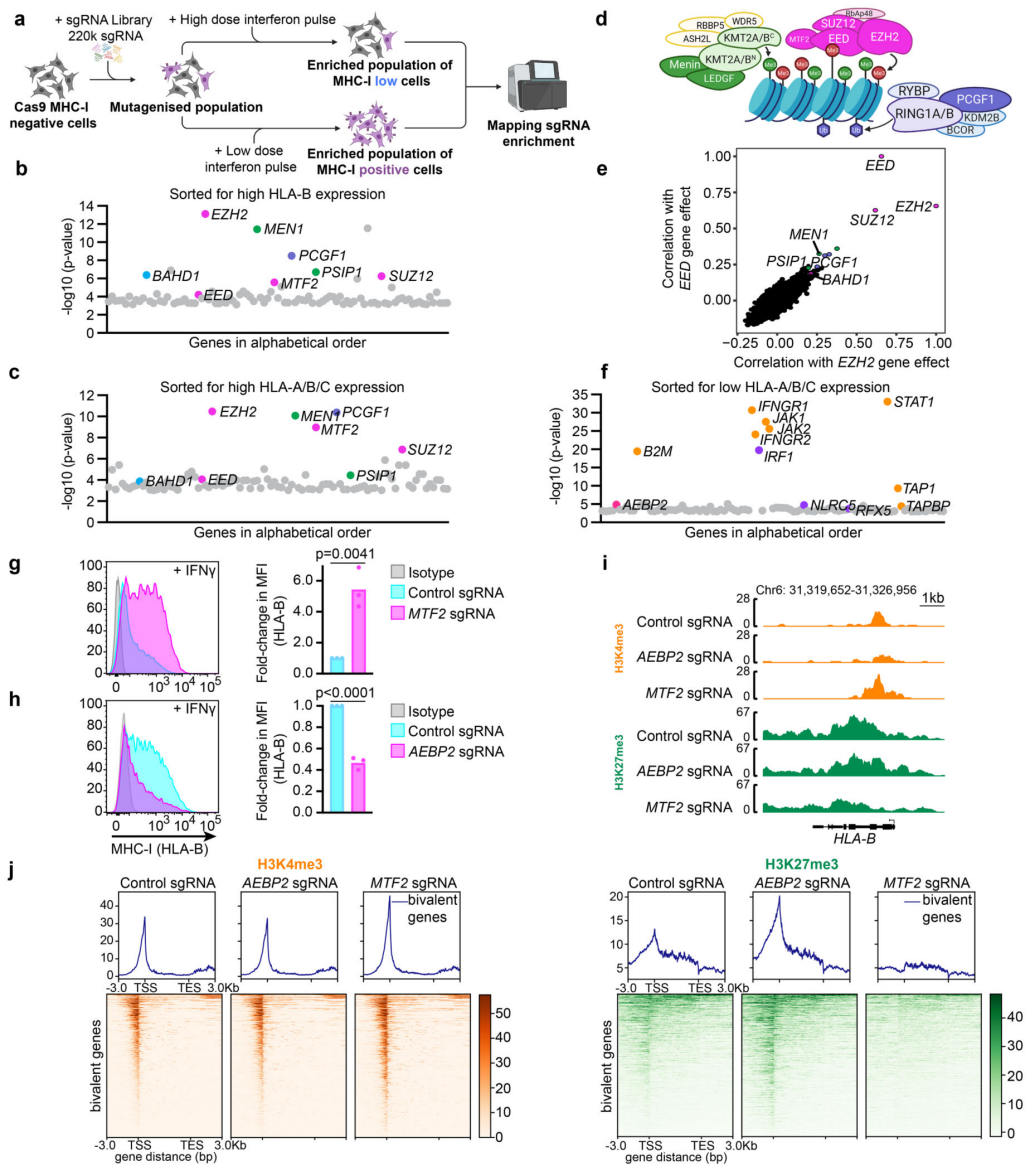
Genome-scale CRISPR/Cas9 screens identify specific polycomb and KMT2complex components regulating bivalent gene activation. (a) Schematic view of CRISPR screens to identify regulators that either enhance or restrict cytokine induced MHC-I expression. K-562 cells were mutagenised by infection with a genome-scale pooled lentiviral library comprising 220,000 sgRNA. Cells were pulsed with 1ng/mL or 25ng/mL IFN-*γ* for 24h prior to enrichment of MHC-I high or low cells by two-rounds of fluorescence-activated cell sorting (FACS). (b & c) Bubble plots show top 100 enriched genes identified in MHC-I high CRISPR screens. PRC2 complex highlighted in pink, PRC1 in purple and the KMT2A/KMT2B complex in green. p-values calculated using the RSA algorithm^83^. (d) Schematic depicting complexes identified in CRISPR screens. KMT2A/B complex (*MEN1*, *PSIP1/LEDGF*), PRC2.1 (*EED*, *EZH2*, *SUZ12*, *MTF2*) and PRC1.1 (*PCGF1*). (e) EED and EZH2 co-dependent genes derived from CERES gene effect scores in combined CRISPR survival screens in 990 cancer cell lines in Cancer Dependency Map (https://depmap.org/portal/)^31,32^. Plot displays correlation between CRISPR gene effect scores for indicated genes with CRISPR gene effect scores for *EZH2* and *EED* (Pearson correlation coefficient). Genes highlighted correspond with hits identified in our CRISPR screen. (f) Bubble plot shows top 100 enriched genes identified in MHC-I low CRISPR screen. Genes highlighted in orange are known components of the interferon response pathway and MHC-I antigen processing pathway, in purple are transcription factors and enhanceosome components driving MHC-I expression, and pink, *AEBP2*, a PRC2.2 component. p-values calculated using the RSA algorithm^83^. (g & h) K-562 Cas9 cells transduced with indicated sgRNA and treated with 10ng/mL IFN-*γ* for (g) 24h or (h) 36h. Cell surface MHC-I from a representative experiment (left). Bars (right) show mean fold change in median fluorescence intensity (MFI) from 3 experiments, indicated by points. Unpaired two-tailed t-test, p-values are (g) p=0.0041 and (h) p<0.0001. (i & j) H3K4me3 and H3K27me3 CUT&Tag in K-562 cells transduced with control, *MTF2* or *AEBP2* sgRNA. (i) Genomic snapshot of bivalent MHC-I gene, *HLA-B*. (j) Heatmaps show bivalent genes -3kb TSS/ +3kb TES. Genomic regions ordered by H3K4me3 or H3K27me3 read density in control samples.

To identify factors regulating transcription factor-driven MHC-I gene activation, we performed genome-scale CRISPR/Cas9 knockout screens in K-562 cells (Fig. 1a), in which we confirmed bivalent modification of MHC-I gene promoters by performing ChIP-reChIP using H3K27me3- and H3K4me3-specific antibodies (Extended Data Fig. 1e/f). As expected, in parallel screens performed with HLA-B- or HLA-A/B/C-specific antibodies, PRC2 components *EED*, *EZH2* and *SUZ12* were amongst the top regulators restricting IFN-*γ* induced activation of bivalent MHC-I genes (Fig. 1b/c, Extended Data Fig 2a/b & Supplementary Tables 1–3). Top candidates included hits identified in screens for repressors of basal MHC-I expression^27^, including polycomb proteins *MTF2* and *PCGF1*, plus *BAHD1*, an H3K27me3 binding protein implicated in polycomb-mediated repression^28–30^. Counterintuitively, our screens also identified *MEN1* and *PSIP1*, which encode KMT2A/B complex components Menin and LEDGF. Although KMT2A/B and polycomb co-occupy bivalent gene promoters, identifying a potential role for Menin and LEDGF in restricting bivalent gene expression contradicts the canonical view of directly opposing functions of these complexes (Fig. 1d).

To investigate the broader significance of these findings, we performed a co-dependency analysis using data from CRISPR survival screens in 990 cancer cell lines within Cancer Dependency Map^31,32^. This analysis revealed a striking concordance with our CRISPR screens assessing the regulation of bivalency (Fig. 1e & Extended Data Fig. 2c). *EZH2* dependency was most closely correlated with a requirement for *EED* and *SUZ12;* and the top 20 *EED* and *EZH2* co-dependent genes included *PCGF1*, *BAHD1*, *MEN1* and *PSIP1*. These data highlight the broader relevance of our approach and imply that interplay between these specific PRC1, PRC2 and KMT2 complex components represents a conserved mechanism of transcriptional control of PRC2-regulated genes across diverse tissue contexts.

### PRC1.1 and PRC2.1 co-operate to restrict bivalent gene activation

**Figure 2:**
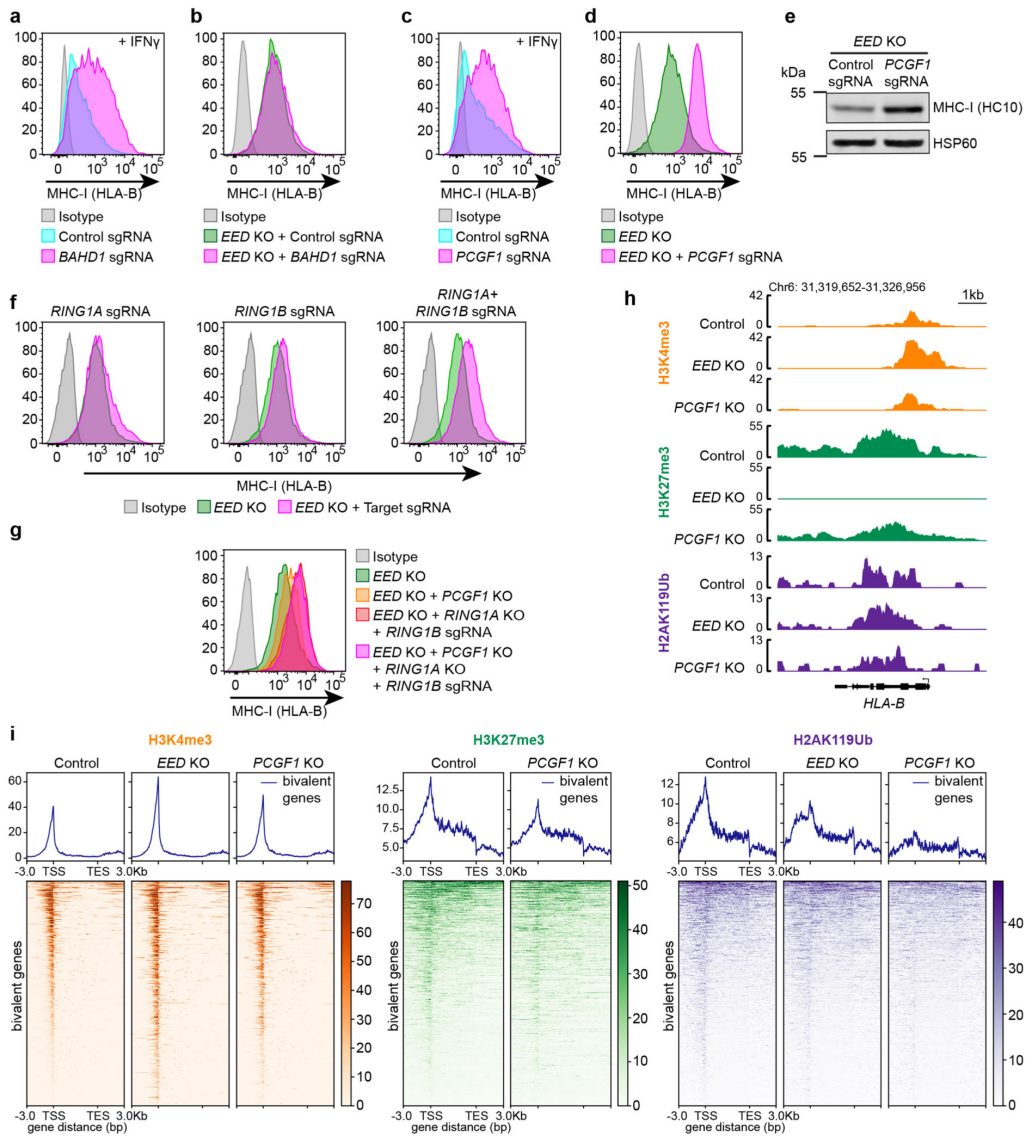
PRC1.1 and PRC2.1 co-operate to restrict activation of bivalent genes. (a-d) Cell surface MHC-I in K-562 Cas9 (a & c) or *EED* KO cells (b & d) transduced with the indicated sgRNA and treated with 10ng/mL IFN-*γ* for 24h as indicated. (e) Immunoblot in *EED* KO cells transduced with control or *PCGF1* sgRNA. (f) Cell surface MHC-I in K-562 *EED* KO cells transduced with *RING1A* and/or *RING1B* sgRNA. (g) Cell surface MHC-I in K-562 cells with indicated knockouts, transduced with *RING1B* sgRNA as indicated. Representative plot from 2 experiments. (h & i) H3K4me3, H3K27me3 and H2AK119Ub CUT&Tag in K-562 Cas9 (control), *EED* KO and *PCGF1* KO cells. (h) Genomic snapshot of bivalent MHC-I gene, *HLA-B*. (i) Heatmaps show bivalent genes -3kb TSS/ +3kb TES. Genomic regions are respectively ordered by H3K4me3, H3K27me3 or H2AK119Ub read density in control samples. (a-d) and (f) show representative plots from 3 experiments (Supplementary Figure 2).

Having previously identified a specific role for the PRC2.1 component *MTF2* in restricting MHC-I gene activation^27^ (Fig. 1b/c), we performed a converse genome-scale CRISPR screen to identify factors required to oppose PRC2.1 and IFN-*γ* induced activation of bivalent MHC-I genes. Intriguingly, this screen identified PRC2.2 complex component *AEBP2* (Fig. 1f & Supplementary Tables 1–3). Consistently, knockout of *MTF2* enhanced, while knockout of *AEBP2* inhibited, IFN-*γ*-induced MHC-I upregulation. Whilst MTF2 knockout dramatically reduced H3K27me3 at bivalent promoters, H3K27me3 was retained or increased at bivalent promoters upon *AEBP2* knockout (Fig. 1g-j & Extended data Fig. 2d-f). This reciprocal function of PRC2.1 and PRC2.2 is consistent with data in embryonic stem cells (ESCs), in which loss of AEBP2 leads to enhanced PRC2.1 activity and H3K27me3 deposition at PRC2.1 target sites^33–35^. Our screens also identified BAHD1, a potential ancillary PRC2 component^28^, as a repressor of bivalent MHC-I gene expression. Additional depletion of BAHD1 in *EED* knockout cells yielded no additional induction of MHC-I, suggesting that BAHD1 contributes to MHC-I gene silencing by reinforcing PRC2.1 activity (Fig. 2a/b & Extended Data Fig. 2g/h). Together, our findings reveal a central role for MTF2-containing PRC2.1 complexes in restricting activation of bivalent genes.

**Extended Data Fig. 3:**
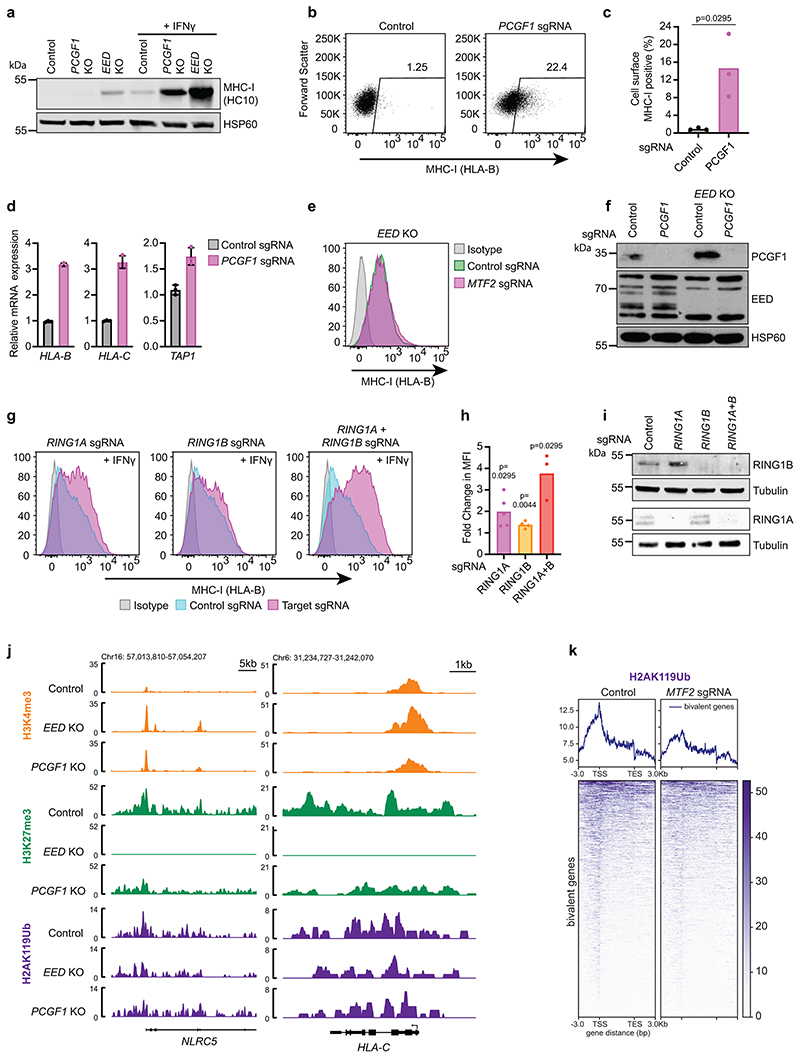
Loss of PRC1 drives derepression of bivalent genes. (a) Immunoblot of K-562 Cas9, *PCGF1* KO and *EED* KO cells ± 10ng/mL IFN-*γ* (40h). (b & c) Cell surface MHC-I in K-562 Cas9 cells transduced with either control or *PCGF1* sgRNA. (c) Bars show mean percentage of MHC-I expression from 3 experiments, indicated by points. Unpaired two-tailed t-test, p=0.0295. (d) qRT-PCR for MHC-I genes in K-562 Cas9 cells transduced with control or *PCGF1* sgRNA. Bars indicate mean ± s.d. of technical triplicates from a representative experiment. (e) Cell surface MHC-I in *EED* KO cells transduced with control or *MTF2* sgRNA. Representative plot from 3 experiments (Supplementary Figure 3). (f) Immunoblot of K-562 Cas9 and *EED* KO cells transduced with control and *PCGF1* sgRNA. (g & h) Cell surface MHC-I in K-562 Cas9 cells transduced with *RING1A* and/or *RING1B* sgRNA, following treatment with 10ng/mL IFN-*γ* for 36h. (h) Bars show mean fold change in MFI from 3-5 experiments, indicated by points. Unpaired two-tailed t-test, p-values are indicated. (i) Immunoblot of K-562 Cas9 cells transduced with indicated sgRNA. (j) Genomic snapshots of bivalent MHC-I genes showing H3K4me3, H3K27me3 and H2AK119Ub CUT&Tag in K-562 Cas9 (control), *EED* KO and *PCGF1* KO cells. The H3K4me3 and H3K27me3 control tracks are the same control tracks in Fig. 6f. (k) H2AK119Ub CUT&Tag in K-562 cells transduced with control or *MTF2* sgRNA. Heatmaps show bivalent genes -3kb TSS/ +3kb TES. Genomic regions are ordered by H2AK119Ub read density in the control sample.

Bivalent genes are defined by PRC2 mediated H3K27me3, however, the contribution of PRC1 to bivalency regulation is less well characterised. Our screens specifically identified PCGF1, a component of variant PRC1.1 complexes^20,36^, as a repressor of MHC-I gene activation (Fig. 2c & Extended Data Fig. 3a-d). PCGF1 containing PRC1 complexes catalyse H2AK119Ub and have been implicated in recruiting PRC2 to chromatin^20^. However, our experiments revealed an additional PRC2-independent contribution of PCGF1 to gene silencing as, in contrast to *MTF2* and BAHD1 loss, PCGF1 depletion in *EED* knockout cells further augmented MHC-I expression (Fig. 2b/d/e and Extended Data Fig. 3e). Other PRC1 components were not identified amongst the top hits in our screens. However, consistent with their known functional redundancy, combined depletion of catalytic PRC1 components RING1A and RING1B substantially enhanced MHC-I expression beyond the loss of PRC2 (Fig. 2f/g and Extended Data Fig. 3f-i). Moreover, the absence of PCGF1 in cells lacking *EED* and RING1A/RING1B failed to further induce MHC-I, indicating that PCGF1 contributes to silencing through PRC1 activity (Fig. 2f/g). The promoters of bivalent genes including MHC-I were marked by H2AK119Ub and PCGF1 knockout led to substantial loss of H2AK119Ub at bivalent promoters, with a corresponding reduction in H3K27me3 and increased H3K4me3. Conversely, EED or MTF2 knockout led to H3K27me3 loss, increased H3K4me3 and reduced, but not absent, H2AK119Ub (Fig. 2h/i & Extended Data Fig. 3j/k). Thus, PCGF1-containing PRC1 complexes maintain H2AK119Ub at bivalent promoters and provide an additional layer of regulation that restrains bivalent gene expression even in the absence of H3K27me3.

### Targeting Menin drives derepression of bivalent genes

**Extended Data Fig. 4:**
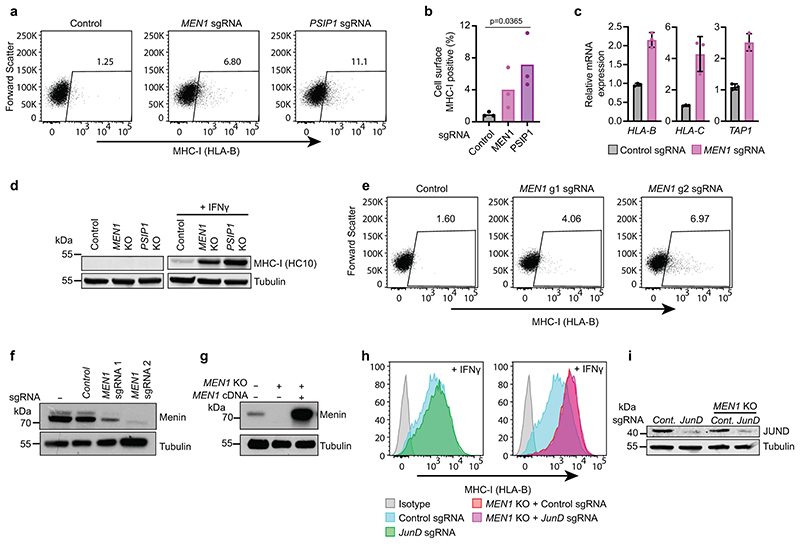
Depletion of Menin or LEDGF enhances basal and IFN-*γ* induced bivalent MHC-I gene expression. (a & b) Cell surface MHC-I in K-562 Cas9 cells transduced with either control, *MEN1* or *PSIP1* sgRNA. (b) Bars show mean percentage of MHC-I expression from 3 experiments, indicated by points. Unpaired two-tailed t-test, significant changes are indicated, p=0.0356. (c) qRT-PCR for MHC-I genes in K-562 Cas9 cells transduced with control or *MEN1* sgRNA. Bars indicate mean ± s.d. of technical triplicates from a representative experiment. (d) Immunoblot of K-562 Cas9, *MEN1* KO and *PSIP1* KO cells ± 10ng/mL IFN-*γ* for 40h. (e) Cell surface MHC-I in K-562 Cas9 cells transduced with control or indicated sgRNA targeting *MEN1*. (f & g) Immunoblots of (f) K-562 Cas9 cells transduced with control sgRNA or sgRNA targeting *MEN1*, (g) *MEN1* KO cells ± *MEN1* cDNA. (h & i) JunD is not required for enhanced MHC-I expression following *MEN1* KO. K-562 Cas9 and *MEN1* KO cells transduced with control or *JunD* sgRNA and analysed by (h) flow cytometry, following treatment with 10ng/mL IFN-*γ* for 48h, and (i) immunoblot. (h) Shows representative plots from 3 experiments (Supplementary Figure 3).

**Figure 3:**
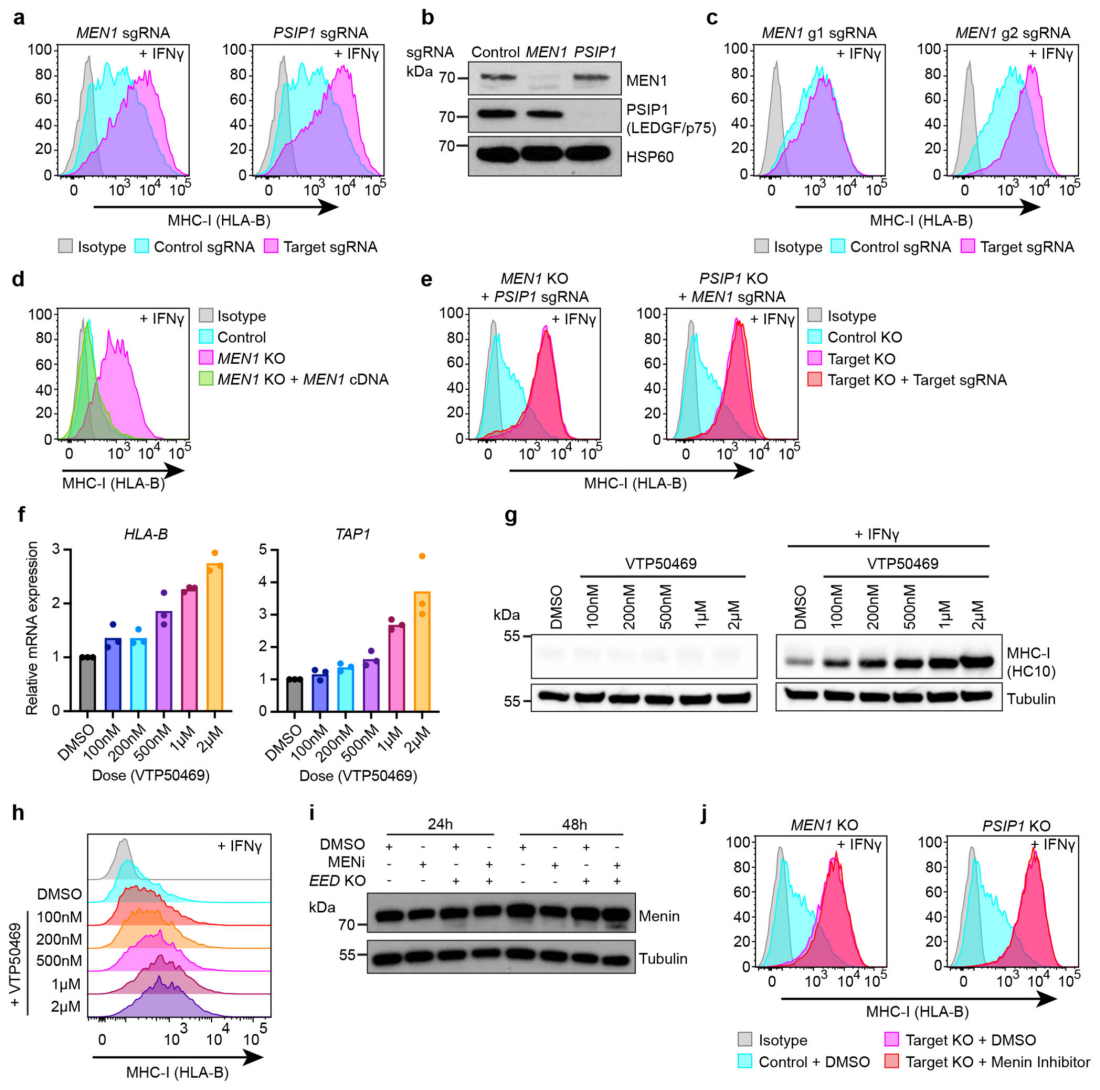
Targeting Menin drives derepression of bivalent genes. (a-c) K-562 Cas9 cells transduced with indicated sgRNA, treated ± 10ng/mL IFN-*γ* (48h) as indicated and analysed by (a & c) flow cytometry (a) from 2 (Supplementary Figure 2), and (c) from 4 experiments (Supplementary Figure 2), and (b) immunoblot. (d) Cell surface MHC-I in K-562 *MEN1* KO cells transduced with a vector encoding *MEN1* cDNA, treated with 10ng/mL IFN-*γ* for 24h. (e) K-562 *MEN1* KO cells transduced with *PSIP1* sgRNA and *PSIP1* KO cells transduced with *MEN1* sgRNA analysed by flow cytometry following treatment with 10ng/mL IFN-*γ* for 36h. (f) K-562 cells treated with indicated doses of VTP50469 for 48h and analysed by qRT-PCR, points indicate 3 independent replicates. (g & h) K-562 cells treated with the indicated doses of VTP50469 for 4 days ± 10ng/mL IFN-*γ* for 40h and analysed by (g) flow cytometry and (h) immunoblot. (i) Menin immunoblot in K-562 and *EED* KO cells treated ± 2μM VTP50469 for 24h or 48h. (j) Cell surface MHC-I in K-562 Cas9, *MEN1* KO or *PSIP1* KO cells treated ± 500nM VTP50469 and 10ng/mL IFN-*γ* for 36h. Representative plots from 2 experiments (Supplementary Figure 2). (d/e) each show representative plots from 3 experiments (Supplementary Figure 2).

Validating the screens, Menin (*MEN1*) or LEDGF (*PSIP1*) knockout augmented basal and cytokine-induced MHC-I expression. The level of MHC-I induction was proportionate to the degree of Menin depletion, and re-expression of *MEN1* cDNA in *MEN1* knockout cells restored MHC-I silencing (Fig. 3a-d & Extended Data Fig. 4a-g). Menin has been reported to repress JUND-mediated transcriptional activation ^37,38^; however, JUND was not required for increased MHC-I expression following Menin depletion (Extended Data Fig. 4h/i). Although Menin and LEDGF have several independent functions, epistatic effects of *MEN1* and *PSIP1* targeting pointed to disruption of KMT2A/B complexes as a potential cause of MHC-I derepression following Menin or LEDGF loss (Fig. 3e).

**Extended Data Fig. 5:**
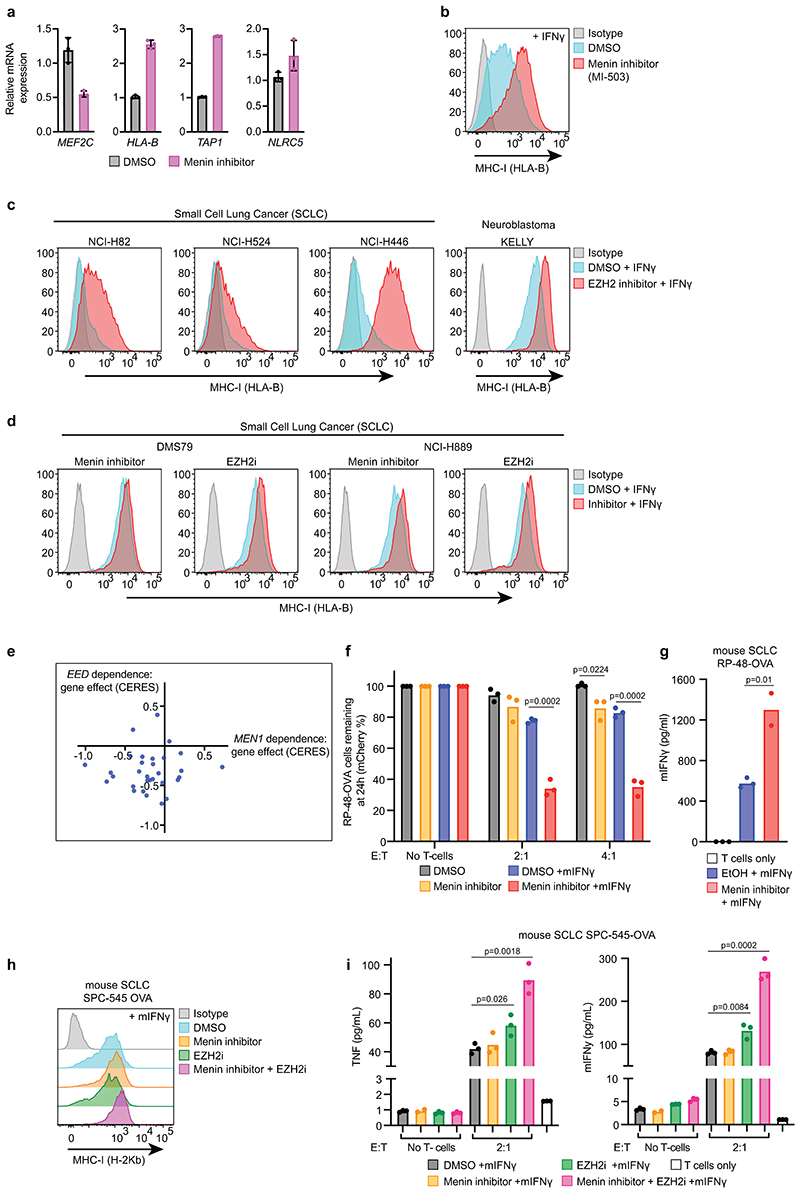
Pharmacological targeting of Menin-KMT2A/B and PRC2 similarly augment IFN-*γ* induced MHC-I expression in MHC-I low cancers and enhance T cell mediated killing. (a) qRT-PCR analysis in K-562 cells treated ± 500nM VTP50469. Bars indicate mean ± s.d. of technical triplicates. (b) MI-503, a chemically distinct inhibitor of the Menin-KMT2A/B interaction, also enhanced IFN-*γ* induced MHC-I expression. Cell surface MHC-I in K562 Cas9 cells pre-treated with 500nM MI-503 and 10ng/mL IFN-*γ* (48h). Representative plot from 3 experiments (Supplementary Figure 3). (c) Cell surface MHC-I in cells treated with DMSO or 3μM EPZ-011989 and 10ng/mL IFN-*γ* (24h SCLC, 40h KELLY), (VTP50469 treatment: Figure 4a). Representative plots from independent experiments (n=2 SCLC, n=3 KELLY (Supplementary Figure 3)). (d) Cell surface MHC-I expression in SCLC cells treated with DMSO, 1μM VTP50469 or 3μM EPZ-011989 and 10ng/mL IFN-*γ* for 24h. Representative plots from 2 experiments (Supplementary Figure 3). (e) Scatter plot indicating *MEN1* and *EED* CERES gene perturbation effects for neuroblastoma cell lines evaluated in combined CRISPR screens in DepMap (DepMap 21Q2 Public+Score, CERES (httos://denman.org/nortal/)^31, 32^ (f) Flow cytometry analysis of RP-48-OVA cells pre-treated with DMSO or 1μM VTP50469 and 10ng/mL murine IFN-*γ* (24h) prior to co-culture with OVA antigen-specific OT-I T cells at the indicated effector:target (E:T) ratios. Bars indicate mean percent remaining mCherry positive (RP-48-OVA) cells compared to no T-cell control from 3 independent replicates, indicated by points. Unpaired two-tailed t-tests compared to respective DMSO controls. Significant changes are indicated. (g) Cytometric Beads Array (CBA) assay for mIFN-*γ* following 24h co-culture of RP-48-OVA cells pre-treated with DMSO or 1μM VTP50469 and 10ng/mL murine IFN-*γ* (24h) prior to co-culture with OVA antigen-specific OT-I T cells at a 2:1 (E:T) ratio. Bars show mean expression from 2-3 independent replicates, indicated by points. Unpaired two-tailed t-test, p=0.01. (h) Cell surface MHC-I in SPC-545-OVA cells pre-treated with DMSO, 1μM VTP50469 and/or 3μM EPZ-011989, and 1ng/mL murine IFN-*γ* (24h). Representative plot from 2 experiments (Supplementary Figure 3). (i) CBA assay for mIFN-*γ* and TNF following 4 days co-culture of pre-treated SPC-545-OVA cells (DMSO, 1μM VTP50469 and/or 3μM EPZ-011989 and 2h 20ng/mL mIFN-*γ*) with OVA antigen-specific OT-I T cells at a 2:1 (E:T) ratio. Bars show mean expression from 3 independent replicates, indicated by points. Unpaired two-tailed t-test compared to respective DMSO +mIFN-*γ* controls. Significant changes are indicated.

To determine whether disruption of the specific interaction between KMT2A/B and Menin drives derepression of bivalent MHC-I genes, we utilised small molecule inhibitors developed for the treatment of KMT2A-rearranged leukaemia^39–42^. Treatment of MHC-I low cells with VTP50469, a specific Menin-KMT2A/B^42^ inhibitor, activated MHC-I gene expression in a dose-dependent manner and enhanced IFN-*γ*-induced MHC-I protein levels (Fig. 3f-h & Extended Data Fig. 5a/b). VTP50469 treatment did not destabilise Menin protein and there was no additional induction of MHC-I following VTP50469 treatment in Menin or LEDGF deficient cells (Fig. 3i/j). Thus, disrupting the Menin-KMT2A/B interaction provides a means to chemically target Menin function and induce derepression of bivalent genes.

### Inhibition of Menin functionally phenocopies EZH2 inhibition

**Figure 4:**
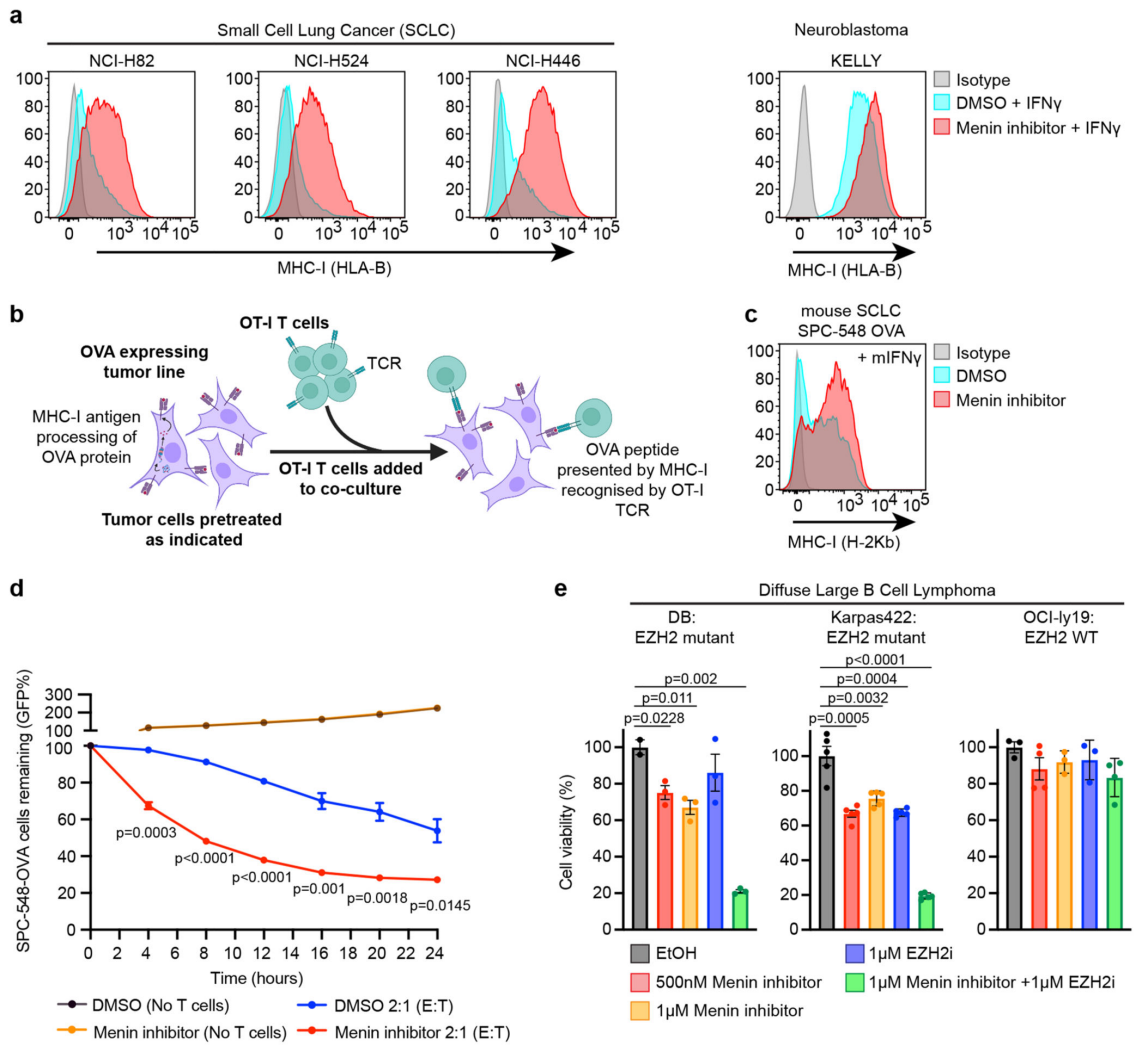
Pharmacological inhibitors targeting the Menin-KMT2A/B interaction drive derepression of bivalent MHC-I genes in MHC-I low cancer cells and enhance T cell mediated tumour killing. (a) Cell surface MHC-I in cells treated ± 1μM VTP50469 and 10ng/mL (SCLC) or 25ng/mL (Neuroblastoma) IFN-*γ* for 24h. Representative plots from 2 (SCLC) and 3 (KELLY) experiments (Supplementary Figure 2). (b) Schematic view of co-culture assay. (c) Cell surface MHC-I in SPC-548-OVA cells treated ± 1μM VTP50469 and 1ng/mL murine IFN-*γ* for 24h. Representative plot from 3 experiments (Supplementary Figure 2). (d) IncuCyte live cell analysis of SPC-548-OVA cells treated ± 1μM VTP50469 prior to co-culture with OVA antigen-specific OT-I T cells at a 2:1 effector:target (E:T) ratio. Points indicate mean ± s.e.m. percent remaining GFP positive (SPC-548-OVA) cells compared to baseline from 3 independent replicates. p-values calculated using unpaired two-tailed t-test comparing Menin inhibitor treated sample to DMSO control. p-values are indicated. (e) CellTiter-Glo assay in specified DLBCL cell lines treated with VTP50469 and/or EPZ-011989 for 5 days. Points indicate mean percent signal relative to vehicle treated control ± s.e.m. Points indicate biologically independent replicates (DB n=2-3, Karpas422 n=5 and OCI-ly19 n=3-4). p-values calculated using unpaired two-tailed t-tests comparing each sample to respective EtOH control. Significant changes are indicated.

Aggressive malignancies such as SCLC and neuroblastoma fail to elicit an effective anti-tumour immune response and exhibit PRC2-mediated silencing of MHC-I genes. Restoring MHC-I antigen presentation in SCLC enables targeting by CD8^+^ T-cells^27^. Treatment of MHC-I low human SCLC and neuroblastoma lines with VTP50469 increased IFN-*γ*-induced cell surface MHC-I, with the degree of induction mirroring the effects of EZH2 inhibitor (EZH2i) EPZ-011989 (Fig. 4a & Extended Data Fig. 5c/d). Neuroblastoma also exhibits cell intrinsic sensitivity to EZH2 inhibition^43^ and CRISPR survival screen data demonstrated frequent *MEN1* and *EED* co-dependency (Extended Data Fig. 5e).

To investigate T-cell responses to SCLC, we expressed ovalbumin (OVA) in SCLC lines derived from mouse models of SCLC driven by inactivation of *Trp53* and *Rb1*, with or without *Myc* overexpression^44–46^. OVA is processed and presented on MHC-I (H-2Kb) and specifically recognised by OT-I T-cells (Fig. 4b). Like the human cancers, these murine SCLC tumours exhibit MHC-I silencing^27^ and treatment of SCLC-OVA with VTP50469 increased IFN-*γ* induced MHC-I levels. Pre-treating cells with VTP50469 prior to co-culture with OT-I T-cells enhanced tumour cell killing and induced greater T-cell IFN-*γ* production (Fig. 4c/d & Extended Data Fig. 5f/g). The SCLC lines exhibited variable MHC-I induction following EZH2i or Menin inhibitor (MENi) treatment and in a line that showed little response to either inhibitor alone, combined treatment enhanced IFN-*γ* induced tumour MHC-I expression and cytokine production from co-cultured T-cells (Extended Data Fig. 5h/i).

To investigate whether MENi could also target tumour-intrinsic PRC2 dependence, we evaluated the effects of VTP50469 on the survival of diffuse large B-cell lymphoma (DLBCL) cell lines harbouring gain-of-function mutations in *EZH2*. These tumours demonstrate sensitivity to EZH2i, which are being evaluated in clinical trials and have been approved for use in *EZH2*-mutant follicular lymphoma^47^. Notably, *EZH2*-mutant DLBCL exhibited similar sensitivity to VTP50469 as EZH2i and combination therapy substantially enhanced tumour killing (Fig. 4e). MENi therefore phenocopies and augments the effects of EZH2i in specific cellular contexts, and can overcome both tumour-promoting and immunosuppressive functions of polycomb.

### Combined Menin and PRC2 targeting augments bivalent gene derepression

**Figure 5:**
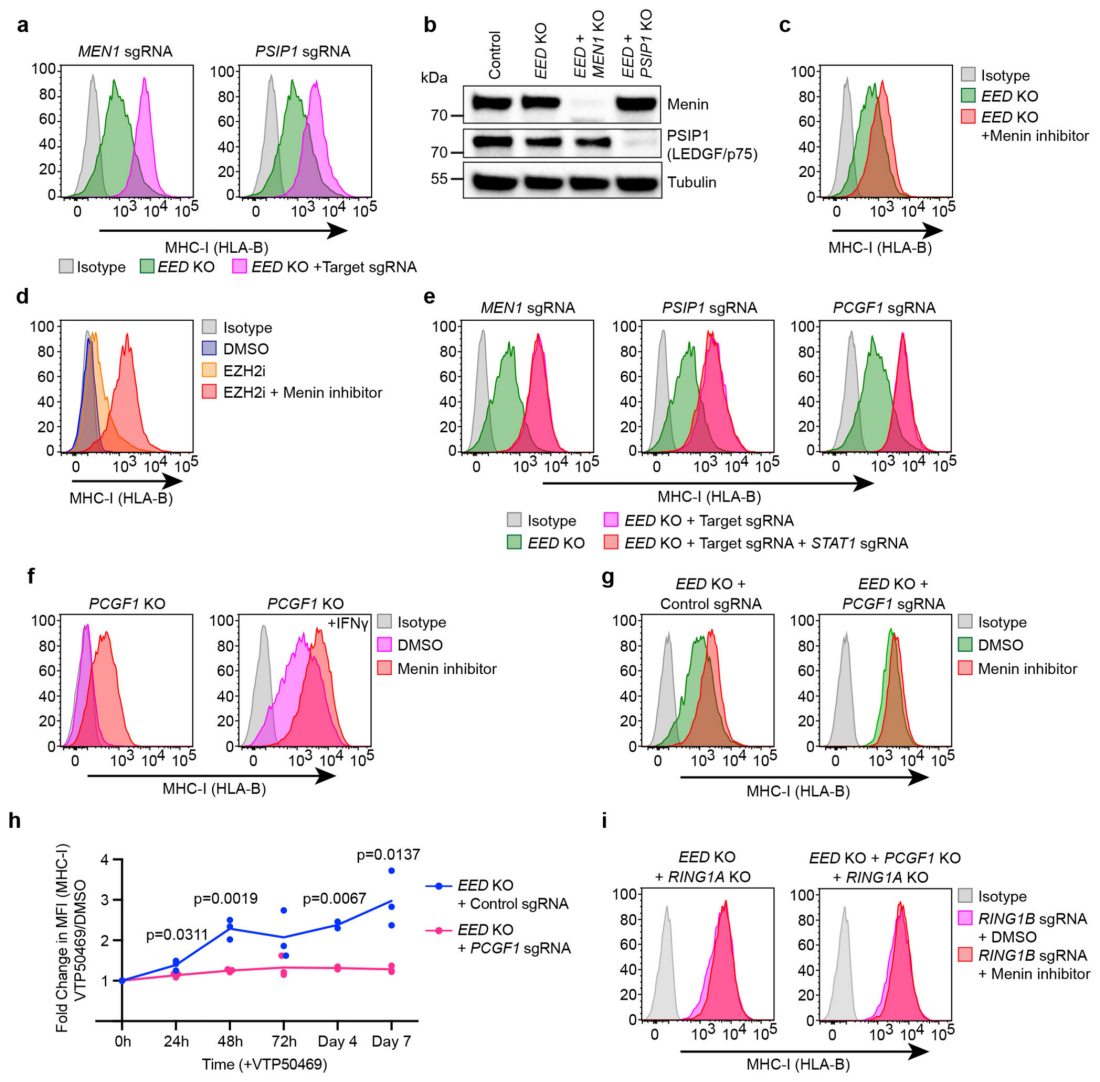
Targeting Menin alleviates polycomb-mediated repression of bivalent genes. (a) Cell surface MHC-I in K-562 *EED* KO cells transduced with indicated sgRNA. (b) Immunoblot in K-562 Cas9 (control) and *EED* KO cells transduced with indicated sgRNA. (c) Cell surface MHC-I in K-562 *EED* KO cells treated ± 500nM VTP50469 for 48h. (d) Cell surface MHC-I in K-562 Cas9 treated with DMSO, 3μM EPZ-011989 ± 500nM VTP50469. (e) Loss of *MEN1*, *PSIP1* and *PCGF1* upregulates MHC-I independently of STAT1. Cell surface MHC-I in K-562 *EED* KO cells transduced with indicated target sgRNA ± *STAT1* sgRNA. (f) Combined targeting of PRC1 and Menin enhances MHC-I expression. Cell surface MHC-I in K-562 *PCGF1* KO cells pre-treated ± 500nM VTP50469 and ± 10ng/mL IFN-*γ* (40h) where indicated. (g-i) Menin inhibition does not further induce MHC-I in polycomb deficient cells. (g & h) Cell surface MHC-I in K-562 *EED* KO cells transduced with control or *PCGF1* sgRNA, treated with 1μM VTP50469 for (g) 48h and (h) indicated time points. Points show mean fold change in MFI from 3 experiments. p-values calculated using unpaired two-tailed t-tests comparing *PCGF1* sgRNA to Control sgRNA at each time-point. Significant changes are indicated. (i) Cell surface MHC-I in K-562 cells with indicated knockouts, transduced with *RING1B* sgRNA and treated with DMSO or 1μM VTP50469. Representative plots from 2 experiments (Supplementary Figure 2). (a) and (c-h) each show representative plots from 3 experiments (Supplementary Figure 2).

MHC-I is not fully derepressed following deletion of *EED* and additional depletion of Menin or LEDGF in *EED* knockout cells augmented MHC-I activation (Fig. 5a/b). Treatment of *EED* knockout cells with VTP50469 recapitulated these findings, and combination with MENi dramatically augmented the capacity of EZH2i to induce cell surface MHC-I (Fig. 5c/d).

**Extended Data Fig. 6:**
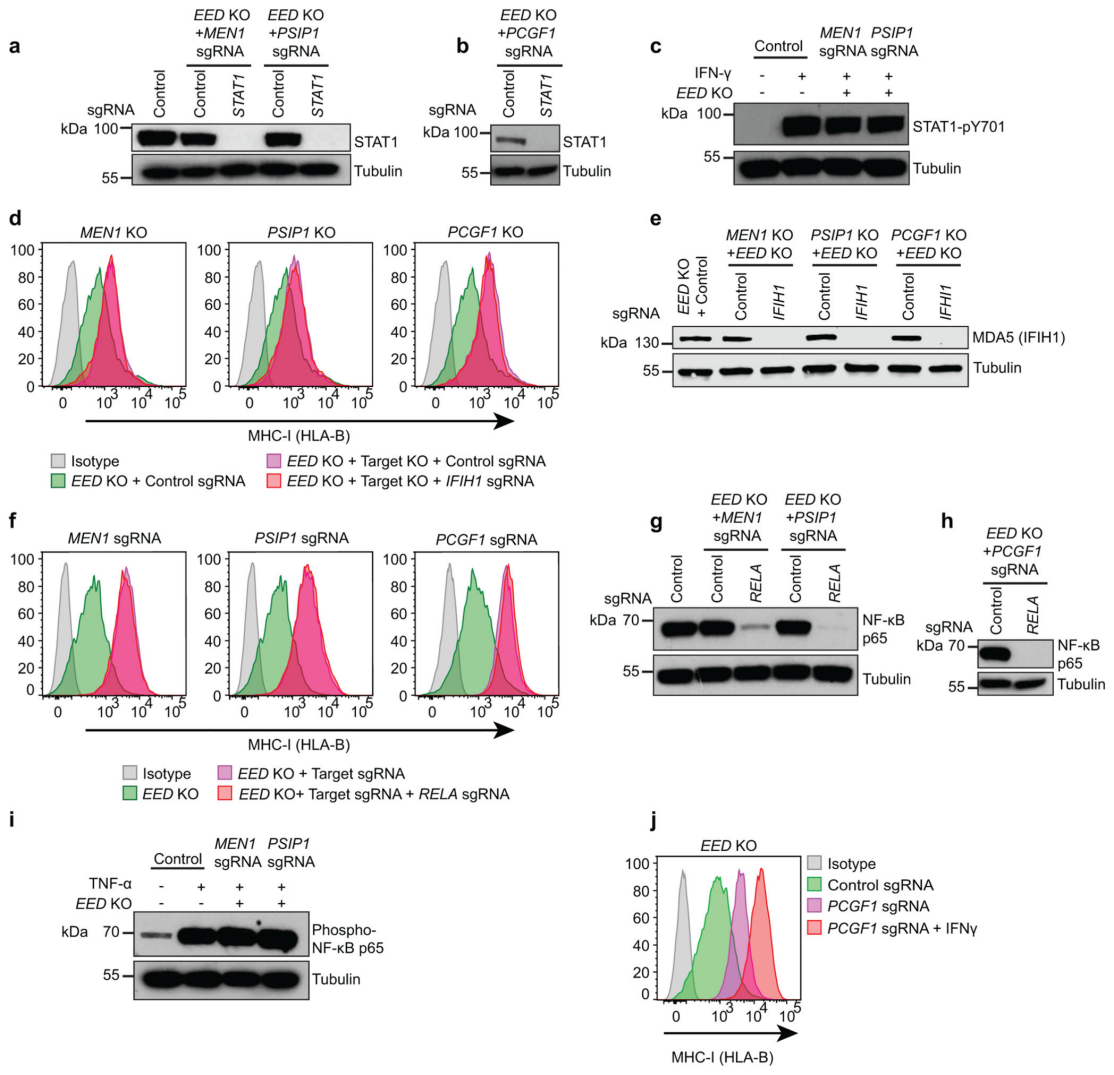
Targeting Menin drives expression of bivalent genes independently of interferon and NFkB signalling. (a & b) Immunoblot in K-562 *EED* KO cells depleted of (a) *MEN1, PSIP1* or (b) *PCGF1*, then transduced with indicated sgRNA. (c) Immunoblot in K-562 Cas9 and *EED* KO cells transduced with indicated sgRNA and treated ± 10ng/mL IFN-*γ* for 48h. (d-h) K-562 *EED* KO cells depleted of *MEN1, PSIP1* or *PCGF1* and transduced with indicated sgRNA, analysed by (d & f) flow cytometry, and (e, g & h) immunoblot. (i) Immunoblot in K-562 Cas9 and *EED* KO cells transduced with indicated sgRNA and treated ± 20ng/mL TNF-α for 48h. (j) Cell surface MHC-I expression in K-562 *EED* KO cells transduced with control or *PCGF1* sgRNA and treated ± 25ng/mL IFN-*γ* for 24h. (d), (f) and (j) each show representative plots from 3 experiments (Supplementary Figure 3).

Inhibition of various epigenetic repressors in cancer cells triggers derepression of endogenous retroviruses, leading to formation of double stranded RNA (dsRNA) and activation of type I interferon signalling in a process dubbed ‘viral mimicry’^48–50^. However, neither depletion of STAT1, which is essential for interferon-induced MHC-I upregulation^51^, nor MDA-5 (*IFIH1*), the key cytoplasmic dsRNA receptor implicated in viral mimicry^48,50^, impaired MHC-I gene activation following Menin targeting (Fig. 5e & Extended Data Fig. 6a-e). Menin has been reported to inhibit NFkB p65-mediated transactivation^52^. However, *RELA* (encodes p65) was not required for MHC-I gene activation following *MEN1* targeting; and there was no increase in TNFα-induced p65 phosphorylation in Menin or LEDGF deficient cells (Extended Data Fig. 6f-i). Therefore, induction of MHC-I expression following Menin loss is not dependent on STAT1- or p65-mediated cytokine signalling.

### Targeting Menin alleviates polycomb-mediated gene repression

Our CRISPR screens and Cancer Dependency Map analyses pointed to interplay between Menin, PRC1.1 and PRC2.1 in the regulation of polycomb-target genes (Fig. 1b-e). Similar to *EED* knockout cells, MENi further increased MHC-I expression in *PCGF1* knockout cells (Fig. 5f). However, disrupting both PRC1 and PRC2 activity by combining *EED* knockout with PCGF1 and/or RING1A/RING1B depletion, largely abrogated the effect of MENi in inducing MHC-I despite the fact that *PCGF1/EED* double knockout cells had not reached the maximum limit of MHC-I expression as evidenced by further MHC-I induction following IFN-*γ* stimulation (Fig. 5g-i & Extended Data Fig 6j). Collectively, these results suggested that targeting Menin activates bivalent genes by alleviating polycomb-mediated repression.

**Extended Data Fig. 7:**
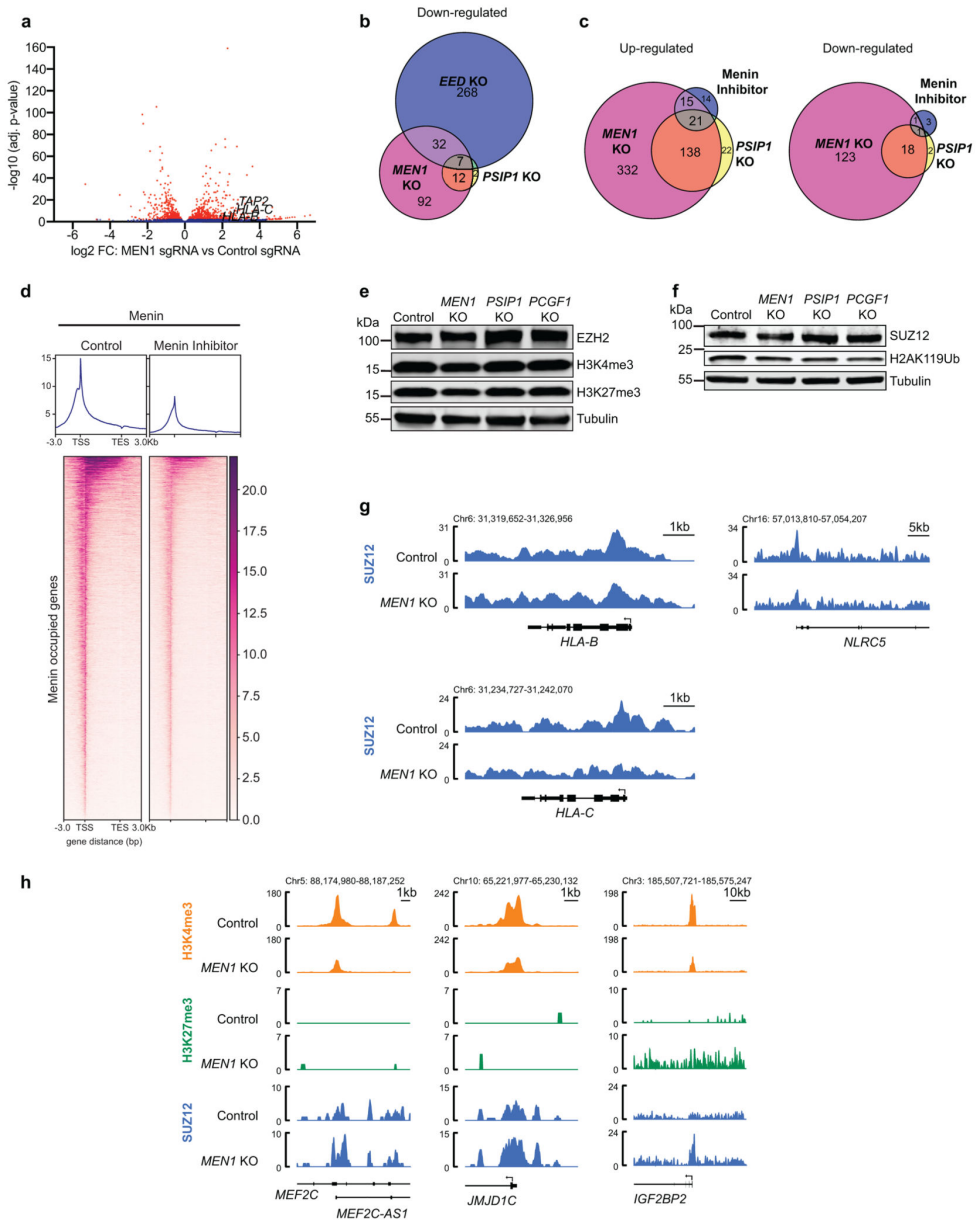
Loss of Menin alleviates repression of bivalent genes. (a) Volcano plot showing Log_2_FC gene expression from RNA-seq data in K-562 cells expressing *MEN1* sgRNA compared with control sgRNA. Selected MHC class I genes are labelled. Two-sided Wald test, p-values adjusted for multiple testing. (b) Venn diagram depicting overlap in genes down-regulated (p-adj <0.05 and fold-change >2) after CRISPR deletion of *MEN1, PSIP1* or *EED*. (c) Venn diagrams depicting overlap in genes up and down-regulated (p-adj <0.05 and fold-change >2) after CRISPR deletion of *MEN1* or *PSIP1* or 500nM VTP50469 treatment. (d) Pharmacological inhibition of Menin-KMT2A/B induces genome wide displacement of Menin from chromatin. Menin ChIP-seq in K-562 cells treated for 48h with DMSO or 1μM VTP50469. Average profile plots (top) and heatmaps (bottom) of Menin occupied sites - 3kb TSS/ +3kb TES. Genomic regions are ordered by Menin occupancy in control sample. (e & f) Immunoblots of K-562 Cas9 (control), *MEN1* KO, *PSIP1* KO and *PCGF1* KO cells. (g) Genomic snapshots of MHC-I genes from SUZ12 ChIP-seq data in K-562 Cas9 control and *MEN1* KO cells. (h) Genomic snapshots of H3K4me3, SUZ12 ChIP-seq and H3K27me3 CUT&Tag in K-562 Cas9 control and *MEN1* KO cells.

**Figure 6:**
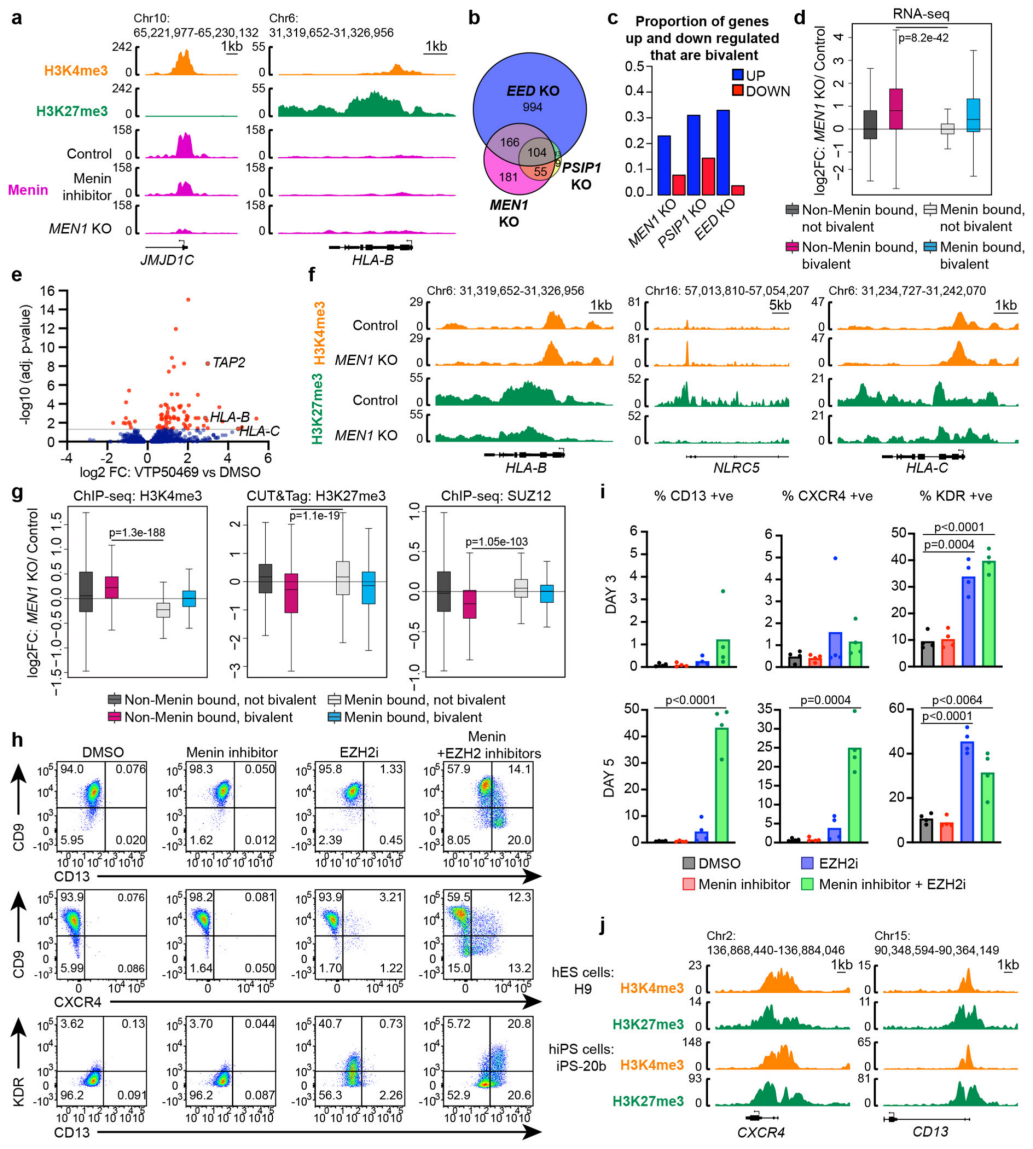
Displacement of Menin from distant genomic loci activates bivalent gene expression. (a) H3K4me3 ChIP-seq and H3K27me3 CUT&Tag in K-562 Cas9 cells, and Menin ChIP-seq data in Cas9, 1μM VTP50469 treated (48h) and *MEN1* KO cells. Genomic snapshots of Menin-KMT2A/B target gene, *JMJD1C*, and bivalent MHC-I gene, *HLA-B*. The H3K4me3 and H3K27me3 tracks are the same control tracks in Fig. 2h. (b) Venn diagram depicting overlap in genes up-regulated (FDR p-adj <0.05 and fold-change >2) after CRISPR deletion of *MEN1*, *PSIP1* and *EED*. (c) Proportion of genes that are bivalent amongst genes showing increased or decreased expression (FDR p-adj <0.05 and fold-change >2) following CRISPR deletion of *MEN1*, *PSIP1* and *EED*. (d) Box-plot depicting Log_2_FC in gene expression in K-562 *MEN1* KO cells compared to Cas9 control from 3 biological replicates. Two-sided Welch’s t-tests, p=8.2e-42. (e) Volcano plot of the top 3,000 genes (Log_2_FC) from RNA-seq in K-562 cells treated with DMSO or 500nM VTP50469. Selected MHC-I genes are labelled. Two-sided Wald test, p-values adjusted for multiple testing. (f & g) H3K4me3, SUZ12 ChIP-seq and H3K27me3 CUT&Tag in K-562 Cas9 control and *MEN1* KO cells. (f) Genomic snapshots of MHC-I genes. The tracks in control cells are the same control tracks in Fig. 2h. (g) Box-plots, from a representative experiment, depicting changes H3K4me3 (left), H3K27me3 (middle) and SUZ12 (right) at Menin and non-Menin bound, bivalent and not-bivalent genes (Log_2_FC). Two-sided Welch’s t-tests, p-values indicated. (h & i) Flow cytometry in human iPSCs treated with VTP50469 and/or EPZ-011989 for 5 days or indicated times. (h) Cell surface CD9, CD13, CXCR4 and KDR. (i) Points show mean percentage of CD13, CXCR4 and KDR positive cells from 4 independent experiments. p-values calculated using unpaired two-tailed t-tests comparing each sample to respective DMSO control. Significant changes are indicated. (j) Genomic snapshots of bivalent genes *CD13* (*ANPEP*) and *CXCR4* showing H3K4me3 and H3K27me3 ChIP-seq data in human embryonic cell line H9 (GEO:GSE96336, GEO:GSE96353) and human induced pluripotent stem cell line iPS-20b (GEO:GSM772844, GEO:GSM772847) ^84^. (d & g) Whiskers represent minimum and maximum, the box represents the interquartile range, and the centre line represents the median.

Menin has been proposed to function as a transcriptional repressor in specific contexts^37,38^. However, while Menin ChIP-seq revealed ubiquitous Menin binding at the promoters of active genes, no significant Menin binding was detected at bivalent MHC-I promoters, arguing against a direct repressive role for Menin at MHC-I genes (Fig. 6a). Following *MEN1* or *PSIP1* knockout, more genes showed increased as opposed to decreased expression (Fig. 6b & Extended Data Fig. 7a/b & Supplementary Table 4). Key candidate repressors of MHC-I identified in the CRISPR screens (such as PRC2.1 or PRC1.1 components) were not significantly downregulated following loss of Menin or LEDGF (Supplementary Table 5). Notably, nearly two-thirds of genes upregulated in Menin/LEDGF knockout cells were also upregulated following *EED* knockout and were enriched for bivalent genes lacking Menin occupancy (Fig. 6b-d). VTP50469 treatment induced remarkably few significant gene expression changes, which is consistent with previous findings in KMT2A-FP leukaemia cells^42^. More genes were upregulated following VTP50469 treatment than were downregulated and there was substantial overlap with genes also upregulated following genetic depletion of Menin or LEDGF (Fig. 6e & Extended Data Fig. 7c). Despite few genes losing expression following VTP50469 treatment, Menin chromatin occupancy was globally reduced (Extended Data Fig. 7d).

To investigate how global loss of Menin from distant sites impacts bivalency, we profiled H3K4me3, H3K27me3 and SUZ12 chromatin occupancy in control and *MEN1* knockout cells. Total levels of H3K4me3, H3K27me3, H2AK119Ub, SUZ12 and EZH2 were unchanged following *MEN1* knockout (Extended Data Fig. 7e/f). At bivalent non-Menin-bound promoters, we observed increased H3K4me3 and reduced H3K27me3 and SUZ12 occupancy following *MEN1* knockout (Fig. 6f/g & Extended data Fig. 7g), implying that Menin is not required for KMT2A/B activity at these genes. In contrast, at active Menin-bound genes, Menin loss led to modestly reduced H3K4me3, and at some genes an associated increase in SUZ12 and H3K27me3 (Fig. 6g & Extended Data Fig. 7h). These results suggested that liberation of KMT2A/B from active genes following Menin loss may alter the equilibrium between KMT2A/B and polycomb at bivalent genes, creating a chromatin environment that is more permissive to gene activation.

### Targeting Menin potentiates bivalent gene derepression in human pluripotent stem cells

**Extended Data Fig. 8:**
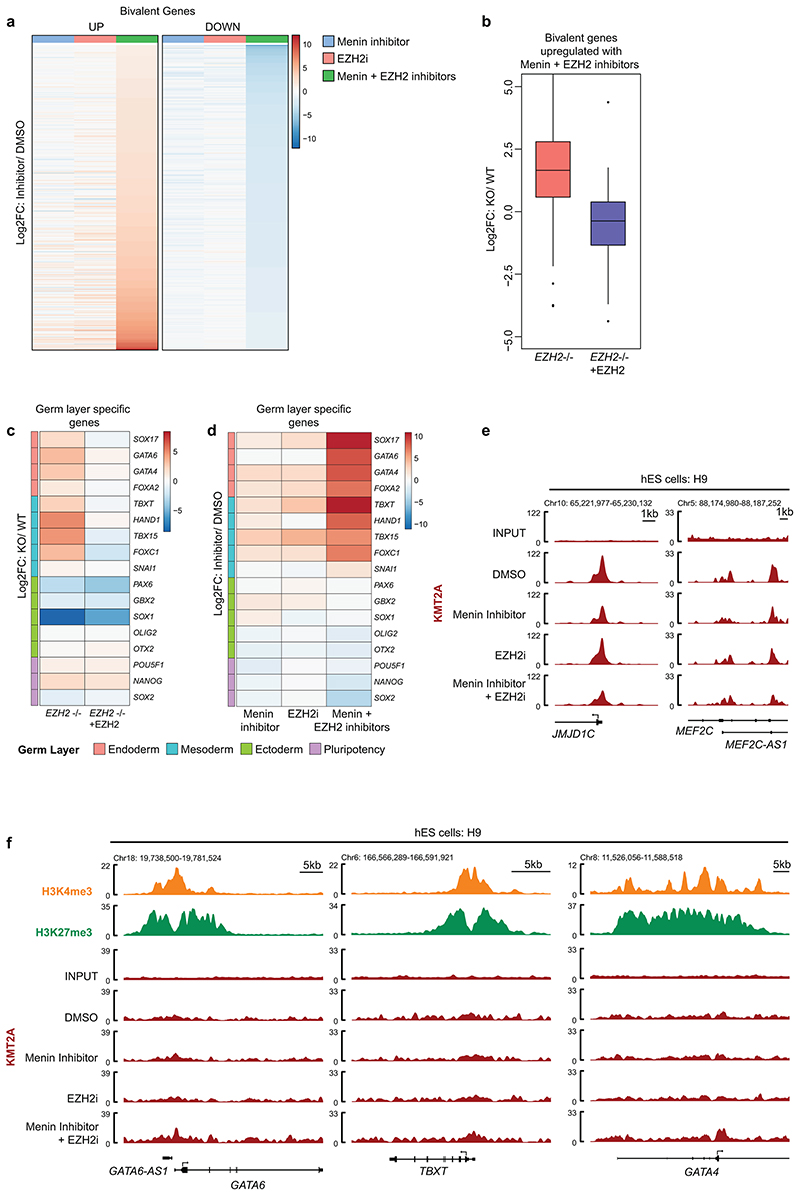
Targeting Menin potentiates bivalent gene derepression in human pluripotent stem cells. (a) RNA-seq in H9 hESCs treated with DMSO, 1μM VTP50469 and/or 3μM EPZ-011989 for 5 days. Heatmap includes bivalent genes significantly up- or down-regulated in combination Menin/EZH2 inhibitor treated cells compared to DMSO control (p-adj <0.05 and Log_2_FC >1 or <-1). (b & c) RNA-seq in wildtype (WT), EZH2null (*EZH2*-/-) and EZH2-complemented EZH2null *(EZH2-/-+EZH2)* H9 hESCs (GEO: GSE76626)^60^. (b) Box-plots include the top upregulated bivalent genes in combination Menin + EZH2 inhibitor treated H9 hESCs (Log_2_FC >4 compared to DMSO control) and depict median Log_2_FC in expression in EZH2null or EZH2-complemented H9 hESCs compared to wildtype control^60^. Whiskers represent the minimum and maximum, the box represents the interquartile range, and the centre line represents the median. (c) Heatmap shows Log_2_FC in expression of selected germ layer specific genes in either EZH2null or EZH2-complemented H9 hESCs compared to wildtype control^60^. (d) Heatmap shows Log_2_FC in expression of selected germ layer specific genes in H9 hESCs treated with 1μM VTP50469 and/or 3μM EPZ-011989 compared to DMSO control. (e & f) ChIP-seq in H9 hESCs. Genomic snapshots showing data from (e) KMT2A, and (f) KMT2A, H3K4me3 (GEO: GSE96336) and H3K27me3 (GEO: GSE96353)^84^.

Polycomb is essential for normal development and in ESCs, lineage specification gene promoters are typically bivalently modified^7,53–59^. EZH2 deletion in human ESCs (hESCs) leads to derepression of polycomb target genes and spontaneous partial differentiation of subsets of cells with a bias towards endoderm and mesoderm lineages^60^. In human induced pluripotent stem cells (iPSCs) derived from peripheral blood mononuclear cells^61^, EZH2i treatment induced expression of the early endodermal marker CXCR4 or mesodermal marker KDR in small subpopulations of cells^62,63^. Combining EZH2i and MENi dramatically enhanced iPSC differentiation, characterised by reduced expression of the stem cell marker CD9 and derepression of CXCR4 and CD13, which exhibit bivalency in hESCs and iPSCs (Fig. 6h-j). To explore genome-wide effects of MENi on bivalent gene regulation, we performed RNA-sequencing in H9 hESCs and integrated with available H3K4me3 and H3K27me3 ChIP-seq. Bivalent genes significantly up- or downregulated following combined EZH2i and MENi treatment showed modest changes in expression with either inhibitor alone (Extended Data Fig. 8a). Supporting our proposal that Menin inhibition potentiates derepression of PRC2 target genes, the bivalent genes most potently upregulated following combined EZH2 and Menin inhibition were modestly upregulated in previously characterised EZH2 null H9 hESCs (Extended Data Fig. 8b) ^60^. Mesodermal and endodermal lineage specific transcription factors amongst the top upregulated genes in EZH2 null hESCs^60^, were also the most highly upregulated genes in MENi and EZH2i treated cells (Extended Data Fig. 8c/d). KMT2A ChIP-seq showed that Menin contributes to KMT2A binding at known active target genes in hESCs but is not required for KMT2A binding at bivalent genes upregulated following EZH2 and Menin inhibition (Extended Data Fig. 8e/f). Altogether, Menin inhibition potentiates derepression of bivalent genes in multiple different cellular contexts, phenocopying and augmenting the effects of PRC2 inhibition on cancer cell viability and immunogenicity, and pluripotent stem cell differentiation.

### Opposing functions of Menin and KMT2A/B in bivalent gene regulation

**Extended Data Fig. 9:**
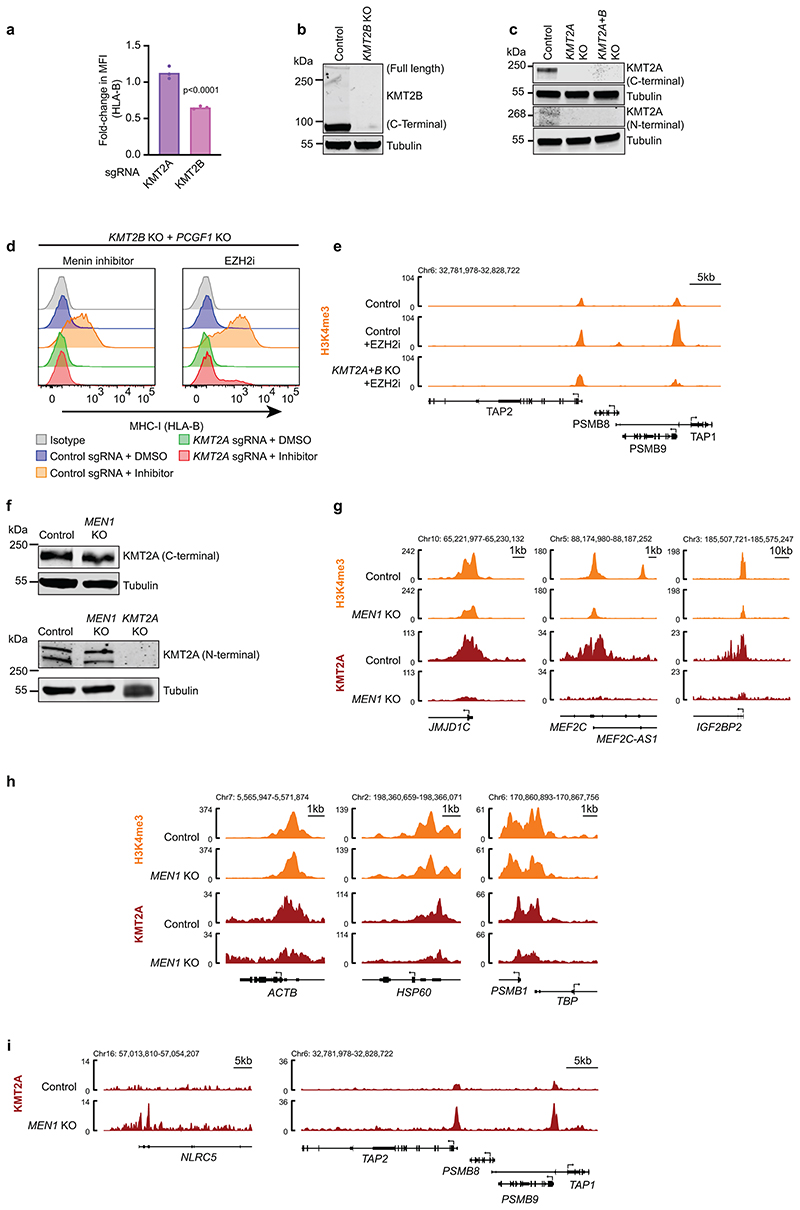
KMT2A/B is required for basal MHC-I expression. (a) Cell surface MHC-I in K-562 Cas9 cells transduced with *KMT2A* or *KMT2B* sgRNA compared to control sgRNA and treated with 10ng/mL IFN-*γ* for 48h. Bars show mean percentage of MHC-I expression from 3 experiments, indicated by points. Unpaired two-tailed t-test compared to control sgRNA. Significant changes are indicated, p<0.0001. (b & c) Immunoblots in K-562 Cas9 and, (b) *KMT2B* KO cells, (c) *KMT2A* KO ± *KMT2B* KO cells. (d) Cell surface MHC-I in K-562 *KMT2B + PCGF1* KO cells transduced with indicated sgRNA and treated for 5 days with DMSO, 1μM VTP50469 or 3μM EPZ-011989. Representative plot from 3 experiments (Supplementary Figure 3). (e) Genomic snapshots of H3K4me3 CUT&Tag in K-562 Cas9 and *KMT2A/B* KO cells treated ± EPZ-011989. The EZH2i treated (no IFN-*γ*) track is also shown in 8g. (f) Immunoblots in K-562 Cas9, *MEN1* KO and *KMT2A* KO cells. (g-i) Genomic snapshots of K-562 Cas9, and *MEN1* KO cells (g & h) H3K4me3 ChIP-seq and KMT2A CUT&RUN. The H3K4me3 tracks are also shown in Extended Data Fig. 7h. (i) KMT2A CUT&RUN.

**Figure 7:**
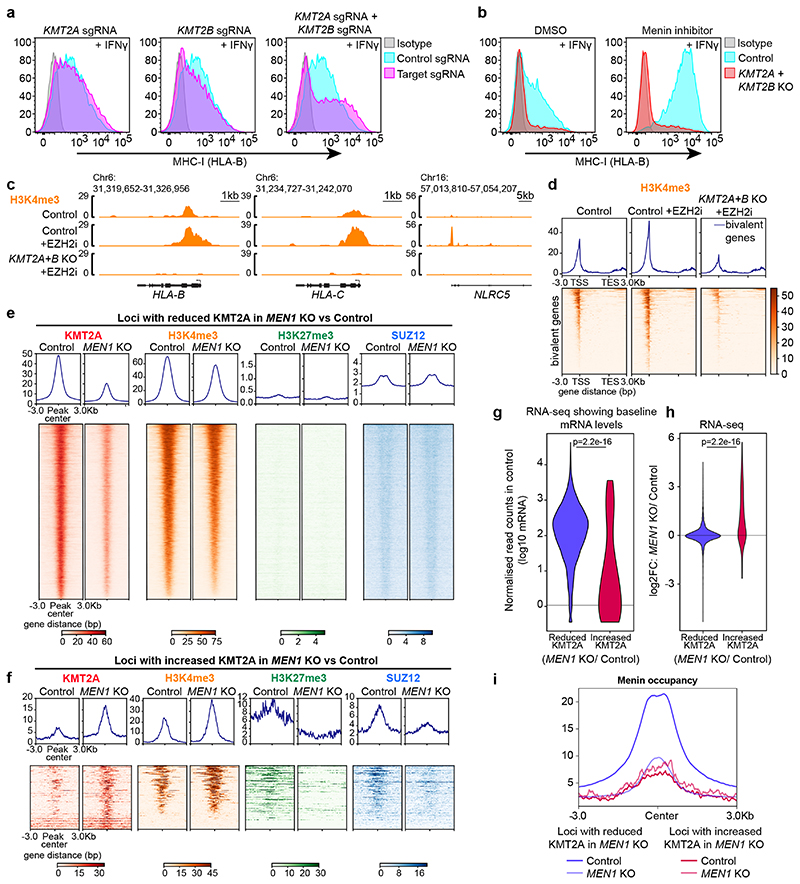
Opposing functions of KMT2A/B and Menin in regulation of bivalent gene expression. (a & b) MHC-I gene expression is dependent on KMT2A/B. (a) Cell surface MHC-I in K-562 Cas9 cells transduced with sgRNA targeting *KMT2A* and/or *KMT2B* (polyclonal population) and treated with 25ng/mL IFN-*γ* for 48h. (b) Cell surface MHC-I in K-562 Cas9 and *KMT2A/B* KO clone pre-treated with DMSO or 1μM VTP50469 and 25ng/mL IFN-*γ* for 36h. (c & d) H3K4me3 CUT&Tag in K-562 Cas9 and *KMT2A/B* KO cells treated ± EPZ-011989. (c) Genomic snapshots of MHC-I genes. The control cell tracks are also shown in Fig. 1i. (d) Average profile plots (top) and heatmaps (bottom) of bivalent genes -3kb TSS/ +3kb TES. Genomic regions ordered by read density in the control sample. (e-i) KMT2A (CUT&Run), H3K4me3, SUZ12 (ChIP-seq) and H3K27me3 (CUT&Tag) in K562 Cas9 control and *MEN1* KO cells. (e & f) Heatmaps show loci with (e) reduced, or (f) increased KMT2A occupancy in *MEN1* KO cells compared to control. Genomic regions are ordered by H3K4me3 read density in control samples. (g/h) Violin-plots show (g) baseline mRNA expression (normalised read counts), and (h) Log_2_FC in gene expression, for genes showing either reduced or increased KMT2A occupancy in *MEN1* KO cells compared to control. Two-sided Wilcoxan t-test, p=2.2e-16. (i) Average profile plot of Menin occupancy from Menin ChIP-seq data in K-562 Cas9 control and *MEN1* KO cells at loci that have reduced or increased KMT2A occupancy in *MEN1* KO cells compared to control. (a/b) show representative plots from 3 experiments (Supplementary Figure 2).

Given that Menin loss unexpectedly increased H3K4me3 at bivalent genes, we investigated the role of *KMT2A* and *KMT2B* in this process. In direct contrast to the effects of *MEN1* deletion or VTP50469 treatment, combined knockout of *KMT2A* and *KMT2B*, impaired IFN-*γ*-induced MHC-I activation (Fig. 7a). Knockout of KMT2B alone modestly impaired MHC-I expression, consistent with a dominant role of KMT2B in maintaining H3K4me3 at bivalent promoters^22,23,53^ (Extended Data Fig. 9a). However, the enhanced effects of combined KMT2A/B depletion indicates that KMT2A can compensate for KMT2B activity at some bivalent genes. Importantly, KMT2A/B were essential to drive the increased MHC-I expression induced following Menin inhibition, and were also required for MHC-I gene expression and increased H3K4me3 at bivalent promoters following disruption of polycomb function (Fig. 7b-d & Extended Data Fig 9b-e). Collectively these data implicate KMT2A/B in promoting bivalent MHC-I gene expression following either Menin or polycomb inhibition.

To investigate the effects of Menin loss on genome-wide localisation of KMT2A, we identified loci that showed either reduced or increased KMT2A occupancy in *MEN1 KO* compared to control cells. Loci showing a significant reduction in KMT2A occupancy upon *MEN1* KO comprised the vast majority of KMT2A bound sites (9,258 of 11,583) and demonstrated high baseline H3K4me3 levels and low/absent SUZ12 and H3K27me3 (Fig. 7e). There was no reduction in total KMT2A protein levels in the absence of Menin (Extended Data Fig. 9f). Despite the dramatic displacement of KMT2A after *MEN1* knockout, only a subset of genes showed a substantial loss of H3K4me3 (Fig. 7e and Extended Data Fig. 9g/h), which is consistent with few genes showing significantly reduced expression following Menin loss and with evidence for a primary role of SETD1A/B in maintaining H3K4me3 at most active genes^64,65^. Conversely, a smaller number of loci, including bivalent MHC-I genes, showed increased KMT2A binding upon *MEN1* knockout (Fig. 7f and Extended Data Fig. 9i). Of the 142 genes which showed increased KMT2A occupancy around the TSS, 63% were concurrently marked by H3K4me3 and H3K27me3 prior to *MEN1* KO. Most were also bound by SUZ12 and showed low basal mRNA expression. Following *MEN1* knockout, these loci showed increased H3K4me3, reduced H3K27me3 and SUZ12, and enhanced gene expression (Fig. 7f-h). Significant baseline Menin binding was not detected at these sites, while loci showing reduced KMT2A upon *MEN1* KO had high baseline Menin occupancy (Fig. 7i). Collectively, these findings support our proposal that Menin loss liberates KMT2A from active genes and leads to redistribution of KMT2A to a subset of transcriptionally silent bivalent genes, which is associated with a shift to a higher H3K4me3:H3K27me3 ratio and increased gene expression. As commercially available reagents to monitor KMT2B binding at chromatin are inadequate, our analyses based only on KMT2A accumulation at bivalent genes likely underestimate the consequences of liberating KMT2A/B from active genes after Menin loss.

### Transcription factor binding bypasses KMT2A/B

**Extended Data Fig. 10:**
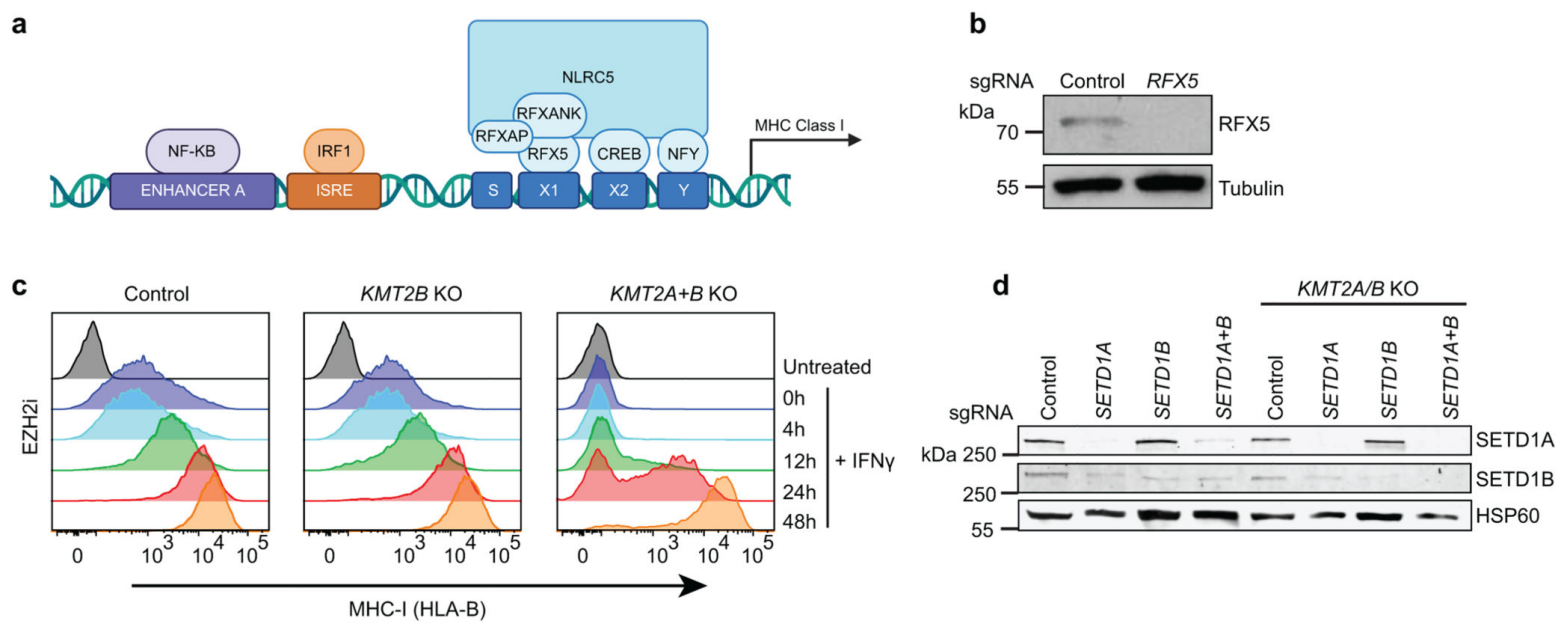
KMT2A/B is dispensable for MHC enhanceosome driven activation. (a) Schematic overview of cis-regulatory elements in the MHC-I promoter. NLRC5 forms an enhanceosome with the RFX (regulatory factor X) complex, made up of RFX5, RFXANK and RFAXP (RFX-associated ankyrin-containing protein); CREB (cAMP-responsive-element-binding); and NFY (nuclear transcription factor Y), which bind the SXY-molecule to activate transcription of MHC-I. (b) Immunoblot of K-562 Cas9 cells transduced with control and *RFX5* sgRNA. (c) IFN-*γ* time-course in K-562 Cas9 and indicated KO cells treated with 3μM EPZ-011989 and 25ng/mL IFN-*γ* for the indicated time points. (d) Immunoblot of K-562 Cas9 and *KMT2A/B* KO cells transduced with control, *SETD1A* and/or *SETD1B* sgRNA.

**Figure 8:**
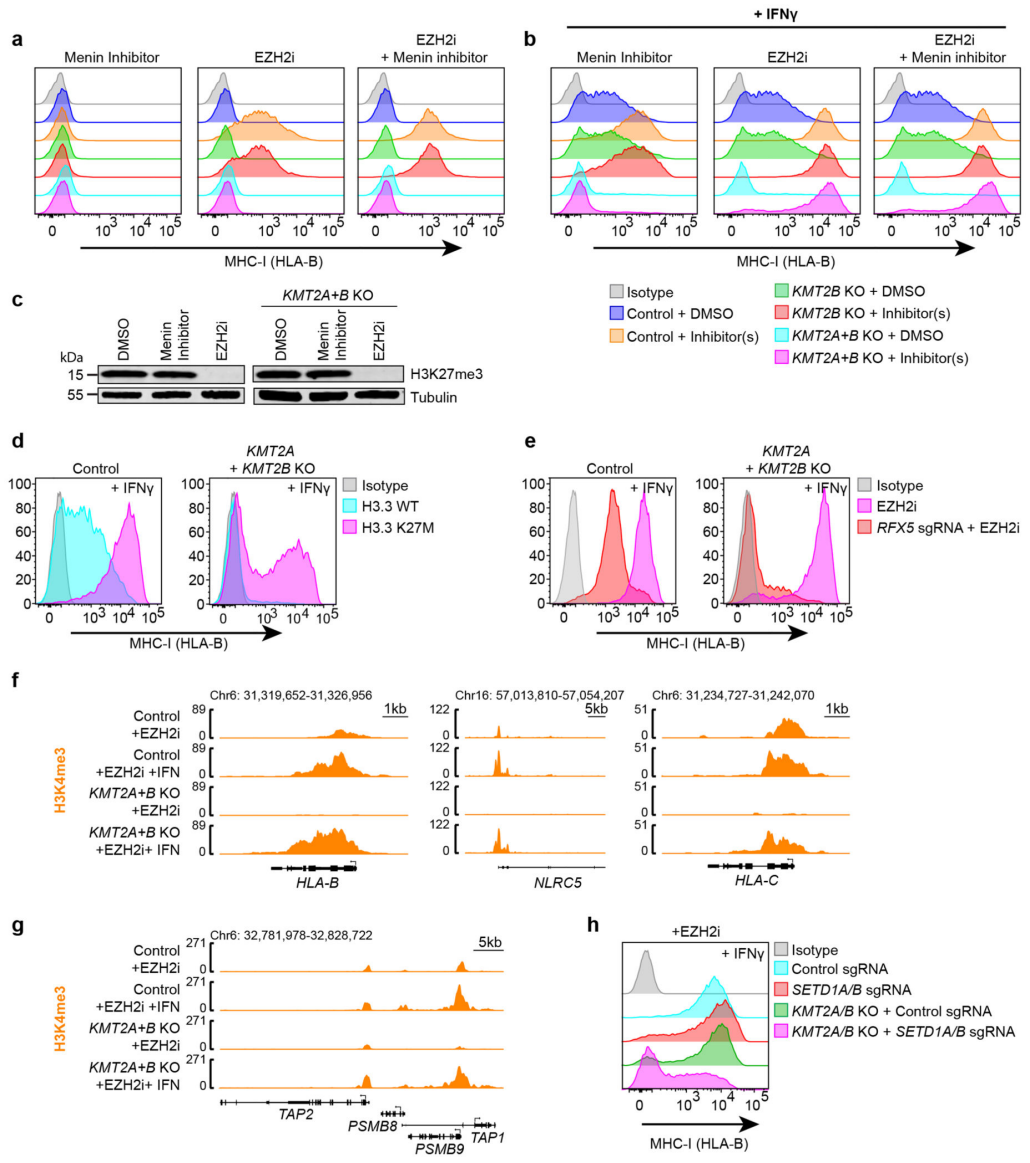
Transcription factor binding bypasses the requirement for KMT2A/B for bivalent gene activation. (a & b) Cell surface MHC-I in K-562 Cas9, *KMT2B* KO and *KMT2A/KMT2B* KO cells treated with 1μM VTP50469 (left), 3μM EPZ-011989 (middle) and combination (right), and (b) treated with 25ng/mL IFN-*γ* for 48h. (c) Immunoblot of K-562 Cas9 and *KMT2A/B* KO cells treated with DMSO, 1μM VTP50469 or 3μM EPZ-011989. (d) Cell surface MHC-I in K-562 Cas9 and *KMT2A/KMT2B* KO cells transduced with lentiviral vectors encoding H3.3 WT or K27M and treated with 25ng/mL IFN-*γ* for 48h. Representative plots from 2 experiments (Supplementary Figure 2). (e) Overcoming dependence on KMT2A/B is reliant on the NLCRC5-RFX5-enhanceosome. Cell surface MHC-I in K-562 Cas9 and *KMT2A/KMT2B* KO cells transduced with *RFX5* sgRNA and treated with 3μM EPZ-011989 and 25ng/mL IFN-*γ* (48h). (f & g) Genomic snapshots of MHC-I genes showing H3K4me3 CUT&Tag in K-562 Cas9 and *KMT2A/B* KO cells pre-treated with EPZ-011989 ± 25ng/mL IFN-*γ* (48h). The EZH2i treated (no IFN-*γ*) tracks are also shown in Fig. 7c. (h) Cell surface MHC-I in K-562 Cas9 and *KMT2A/B* KO cells transduced with control or *SETD1A* + *SETD1B* sgRNA, treated with EPZ-011989 and 25ng/mL IFN-*γ* (48h). Representative plot from 4 experiments (Supplementary Figure 2). (a/b) and (e) are representative plots from 3 experiments (Supplementary Figure 2).

Our findings highlighted a critical role for KMT2A/B in opposing polycomb at bivalent genes. To investigate whether KMT2A/B are required for bivalent MHC-I gene transcription once polycomb-mediated repression is removed, we pre-treated cells with MENi and/or EZH2i to create a permissive environment for transcription factor binding, and then added IFN-*γ*. Strikingly, in the absence of H3K27me3, IFN-*γ* induced similarly robust MHC-I expression in control and *KMT2A*/*KMT2B* knockout cells (Fig. 8a-c). A similar bypass of the KMT2A/B requirement was observed following expression of H3.3 K27M (Fig. 8d), an oncohistone variant H3 that inhibits H3K27me3 spreading^66,67^. The major transcription factors required for IFN-*γ*-induced MHC-I expression include IRF1 and the MHC-I transactivator NLRC5^68^. NLRC5 localises to MHC-I promoters through association with the MHC enhanceosome, a multisubunit complex of which RFX5 is a core component (Extended Data Fig. 10a/b). In contrast to control cells, IFN-*γ* induced MHC-I expression in dual *KMT2A/KMT2B* knockout cells was entirely dependent on the NLRC5-RFX5-enhanceosome, confirming an essential role for this transcription factor complex in bypassing the KMT2A/B requirement for bivalent MHC-I gene activation (Fig. 8e). Although maximal MHC-I expression was not impaired, *KMT2A/KMT2B* knockout cells showed a variable delay in MHC-I activation (Extended Data Fig. 10c). Thus, although KMT2A/B and baseline H3K4me3 are not absolutely required to induce MHC-I gene expression, they may facilitate uniform transcriptional activation.

Despite the close correlation between promoter H3K4me3 levels and transcription, whether H3K4me3 is required for gene expression remains unclear. Whilst the block in EZH2i induced MHC-I gene activation in *KMT2A/B* knockout cells was associated with loss of promoter H3K4me3; the bypass of *KMT2A/B* for MHC-I gene activation after exposing EZH2i treated cells to IFN-*γ*, was associated with complete H3K4me3 restoration (Fig. 8f/g). Additional depletion of the H3K4me3 methyltransferases SETD1A and SETD1B in KMT2A/B knockout EZH2i treated cells, blocked RFX5-dependent MHC-I activation (Fig. 8e-h & Extended Data Fig. 10a-d). Our findings highlight distinct functions for mammalian H3K4me3 methyltransferase complexes in regulating bivalent MHC-I gene expression. KMT2A/B are required to counteract PRC2-mediated repression at bivalent genes. However, in the absence of restriction from PRC2 activity, transcription factor driven gene expression can be sustained by compensation from SETD1A/B, which maintain tight coupling between promoter H3K4me3 and transcription.

## Discussion

Bivalent chromatin is a hallmark of genes governing cell fate, which frequently become deregulated in cancer. Our genome-scale screens identified roles for PCGF1-PRC1.1 and MTF2-PRC2.1 in bivalent gene regulation. Whilst mutually co-operative, the additional independent contributions of these two complexes to restriction of bivalent gene activation may reflect capacity of both MTF2 and KDM2B to associate with CpG islands and recruit PRC2.1 and PRC1.1 to bivalent promoters^15,69–73^. We observe partial retention of H2AK119Ub at bivalent promoters following *EED* knockout and recent studies support PRC2-independent effects of H2AK119Ub in gene silencing^74,75^.

Outside its role in KMT2A-FP-driven leukaemia, the precise function of Menin in chromatin regulation has remained largely enigmatic. Here we identify an unexpected facet of Menin function, showing that Menin contributes to silencing of bivalent genes by preserving a balance of polycomb and KMT2A/B activity. Menin-dependent recruitment of KMT2A to target genes is well established; however, evidence also supports Menin-independent KMT2A/B function. In mouse ESCs, the CXXC domain of KMT2B can mediate recruitment to chromatin^76,77^ and in KMT2A-FP leukaemia cells there is minimal overlap between genes differentially expressed following Menin deletion compared to combined KMT2A/B deletion^78^. Furthermore, inhibitors targeting the Menin-KMT2A or KMT2A-WDR5 interaction induce distinct transcriptional changes^79^.

We propose that release of KMT2A/B from active genes upon Menin knockout or inhibition, leads to redistribution of KMT2A/B to bivalent promoters. Increased KMT2A/B and H3K4me3 opposes polycomb-mediated repression and is associated with a modest increase in transcription at some genes and an enhanced response to transcription factor induced activation. Synthetic CpG rich DNA inserted into the genome acquires H3K4me3 independently of CFP1, implying that CpG islands can drive KMT2A/B recruitment^3,23,76,80,81^. Additional interactions, for example with WDR5 or with H3K4me3 via its PHD domains, could reinforce KMT2A/B binding at bivalent loci. KMT2B also regulates H3K4me3 at some enhancers controlling bivalent developmental genes^53,76,82^, providing another potential mechanism by which targeting Menin could regulate bivalent genes. Intriguingly, inactivating mutations in *MEN1* can drive neuroendocrine tumour development. PRC2 components can have either tumour suppressive or oncogenic functions depending on the cellular context^24^, raising the possibility that dysregulation of bivalent gene expression could play a role in the tissue-specific tumour development associated with Menin loss.

Future comprehensive molecular and biochemical studies will be required to understand what drives the differential localisation of Menin and KMT2A/B in the genome, and how this influences polycomb function. However, our findings provide an important paradigm that reveals therapeutic opportunities. We show that targeting Menin enhances T-cell killing of MHC-I low cancer cells, providing a rationale for using MENi to enhance immunotherapy responses in malignancies exhibiting polycomb-mediated silencing of MHC-I. Moreover, the dependency of many cancers on EZH2 highlights broader therapeutic applications. EZH2i are in clinical trials for diverse malignancies and the striking EZH2 and Menin co-dependency suggests that sensitivity to EZH2i may predict tumours likely to also respond to MENi. The additive effects of Menin and EZH2 inhibition in alleviating polycomb-mediated repression also provides a basis for rational therapeutic combinations. Our findings highlight how understanding the processes controlling bivalency can reveal approaches to target gene dysregulation in cancer that arises due to perturbation of polycomb and trithorax function.

## Methods

All animal experiments presented in this study were conducted according to regulatory standards approved by the Peter MacCallum Cancer Centre Ethics and Experimentation committee.

### Cell lines

Human neuroblastoma cell line Kelly were a gift from Paul Ekert (Murdoch Children’s Research Institute). Human SCLC line NCI-H82 were a gift from Jonoathan Yewdell (National Institute of Allergy and Infectious Diseases, Bethesda) and NCI-H524, NCI-H889, NCI-H446 and DMS79 from (Christopher Vakoc, CSHL, New York). HEK-293ET cells were a gift from Dr. Felix Randow (MRC-LMB, Cambridge, UK). H9 and PB010 iPS cell lines were kindly provided by Dr. Katerina Vlahos (MCRI)^61^. The following cells were acquired from ATCC, DSMZ or CellBank Australia as indicated: K-562 (ATCC, CCL-243), Drosophila S2 (ATCC, CRL-1963), human diffuse large b-cell lymphoma (DLBCL) DB (ATCC, CRL-2289), Karpas422 (CellBank Australia, 06101702) and OCI-ly19 (DSMZ, ACC 528). Mouse SCLC cell lines RP-48, SPC-545 and SPC-548 were established from primary lung tumours in genetically engineered models of SCLC driven by conditional bi-allelic inactivation of the tumour suppressor genes *Trp53* and *Rb1* (p53Rb)^45^, with (SPC-545 and SPC-548) or without (RP-48) *Myc* overexpression. RP-48 cells were isolated from mice carrying conditional alleles for Rb1 (floxed exon 19) and Trp53 (floxed exons 2–10), following intra-nasal delivery of an adenoviral CMV-Cre-recombinase vector to p53Rb mice resulting in somatic inactivation of *Trp53* and *Rb1* in pulmonary cells and the subsequent development of high-grade neuroendocrine tumours phenocopying human SCLC^45^. Tumours were mechanically disaggregated and cultured in DMEM/F12 (Gibco) with 15 mM HEPES, supplemented with 2 mM Glutamax (Invitrogen), 100 IU/mL Penicillin, 100 μg/mL Streptomycin, 4 μg/mL Hydrocortisone (Sigma-Aldrich), 5 ng/mL EGF (Invitrogen), 5 mL of Insulin-Transferrin-Selenium solution (Life Technologies) and 10% HI-FCS. SPC-545 and SPC-548 were isolated from mice carrying conditional alleles for *Rb1* (stock number 026563) and *Trp53* (stock number 008462) as above, plus LSL-Myc (stock number 020458), following intranasal delivery of adenoviral Ad5-SPC-Cre (VVC-Berns-1168). High-grade neuroendocrine tumours in the lungs of mice were diced in HBSS and dissociated in HBSS, 2% FCS, 2.5mM EDTA and 10mM HEPES for 30 minutes at 37°C. Cells were collected by centrifugation at 900g and digested in a collagenase solution (RPMI, 5% FCS, 1mg/ml Collagenase type 3 and 4ng/ml DNAse I) for 90 minutes at 37°C. Cells were passed through a 70μm strainer, resuspended in 44% iso-osmotic percoll, layered on top of 66% Percoll and centrifuged at 1800g for 20 minutes without brakes. Tumour cells were recovered from the Percoll concentration interface, washed in media and cultured in Advanced RPMI supplemented with 1% FCS, antibiotics and glutamax. Cells were validated by genotyping PCR. SPC-545, SPC-548 and human cell lines, were cultured in RPMI-1640 or DMEM (HEK-293ET) supplemented with 2 mM Glutamax, 100 IU/mL Penicillin, 100 μg/mL Streptomycin and 10% HI-FCS. CD8 T cells isolated from mice were cultured in complete RPMI-1640 media (10% HI-FBS, 100 IU/mL Penicillin, 100 μg/mL Streptomycin, 1 mM sodium pyruvate, 2 mM Glutamax and 55 μM 2-mercaptoethanol). H9 and iPS cell lines were maintained in mTESR Plus medium on 1% Geltrex coated plates, and routinely passaged using EDTA dissociation buffer in 1:8 ratio, in presence of 2mM Thiazovivin. Drosophila S2 cells were cultured in Schneider’s Drosophila media (Life Technologies) supplemented with 100 IU/mL Penicillin, 100 μg/mL Streptomycin and 10% HI-FCS. All cell lines were cultured in 5% CO2 at 37°C, except Drosophila S2, which were cultured at room temperature.

Cell lines were authenticated by STR profiling through the Australian Genome Research Facility (Melbourne, Victoria). Cell lines were regularly tested and verified to be mycoplasma-negative by PCR analysis through the Victorian Infectious Diseases Reference Laboratory (Melbourne, Victoria).

### Chemicals

VTP50469 was a kind gift from Syndax Pharmaceuticals and subsequently purchased from MedChemExpress (MCE) (Cat# HY-114162; CASRN: 2169916-18-9). EPZ-011989 and MI-503 were purchased from Selleck Chemicals (Cat# S7805; CASRN: 1598383-40-4, Cat# S7817; CASRN: 1857417-13-0, respectively). Titration and time-course experiments were performed for inhibitors and cytokines. For inhibitors, cells were treated for 7-10 days with 0.5-1μM VTP50469, 500nM MI-503 or 3-5μM EPZ-011989 prior to analysis (unless specified in figure legends), and refreshed 3 times per week. For interferon induction of MHC-I expression in validation experiments, human cells were treated with 10-25ng/mL recombinant human interferon gamma (Sigma-Aldrich, Cat# SRP3058) for 24-48 h prior to analysis (specified in figure legends). This concentration range provided more reliable induction of HLA-B than the 1ng/ml dose used in the MHC-I high CRISPR screens. Mouse cells were treated with recombinant mouse interferon gamma (Abcam, Cat# ab123747) at 10ng/mL for 24 h prior to analysis (or as specified in figure legend). For TNF alpha induction of MHC-I expression, human cells were treated with recombinant human TNF-α (Abcam, Cat# ab259410) at 20ng/mL for 48 h prior to analysis.

### Plasmids

pBABE hygro MEN1 WT was a gift from Matthew Meyerson (Addgene 11024)^85^. pCDH-EF1-Puro lentiviral vectors encoding HA-FLAG-tagged wildtype H3.3 and H3.3 K27M were a kind gift from Peter Lewis (University of Wisconsin)^86^. All plasmids were verified by Sanger sequencing analysis through the Australian Genome Research Facility (Melbourne, Victoria). An ovalbumin (OVA) expressing retroviral vector was generated by PCR amplification of full-length chicken OVA from pcDNA3-OVA (Addgene 64599, a gift from Sandra Diebold & Martin Zenke)^87^ and cloned into both pMSCV-IRES-mCherry FP (a gift from Paul Beavis) and pMSCV-IRES-GFP (modified from pMSCV-IRES-mCherry FP) via EcoRI and XhoI sites.

### CRISPR sgRNA Library

The screen used the Bassik Human CRISPR KO Library (a gift from Michael Bassik, Addgene 101296-101934). This 10-sgRNA-per-gene CRISPR/Cas9 deletion library targets all ~20,500 protein-coding human genes and contains both non-targeting control sgRNA with no binding sites in the genome and safe-targeting sgRNA targeting genomic locations with no annotated function, details described in Morgens DW et al^88^. Library sgRNAs are expressed in the pMCB320 lentiviral sgRNA expression vector, encoding puromycin and mCherry selection markers.

### CRISPR Screen

K-562 cells were transduced with a lentiviral vector encoding Cas9 and selected with blasticidin. For the screen, 10^8^ K-562 Cas9 cells were infected with the pooled lentiviral genome-wide sgRNA library at a multiplicity of infection of 0.3. Percentage of mCherry positive (sgRNA expressing) cells was assessed at 72 hours by flow-cytometry. Infected cells were selected with 1μg/mL puromycin for 72 hours, commencing 48 hours after transduction. To avoid non-specific background cells in the sorting gates, cells were either pulsed with IFN*γ* at a low dose of 1ng/mL or high dose of 25ng/mL 24 hours prior to FACS sorting, then enriched for MHC-I positive and MHC-I low cells, respectively, by two rounds of sorting at day 18 and 25 (positive) and day 7 and 15 (low) post transduction. Cells were sorted in parallel, stained with either APC-conjugated anti-human HLA-A,B,C specific antibody (W6/32, Biolegend) or APC-conjugated anti-human HLA-B specific antibody (REA143, Miltenyi Biotec), incubated on ice for 15 minutes and washed with PBS plus 2% FCS prior to sorting for mCherry positive (sgRNA expressing) MHC-I positive or low cells on the BD FACSAria Fusion or BD Influx cell sorter. Genomic DNA was extracted (Puregene Core Kit A, Qiagen) from sorted cells and an unselected pool of mutagenised cells grown in parallel. sgRNA sequences were amplified by two rounds of PCR, with the second round primers containing adaptors for Illumina sequencing. Samples were sequenced with single-end 50 bp or 75 bp reads on an Illumina HiSeq or NextSeq500, respectively. Sequence reads were trimmed to remove the constant portion of the sgRNA sequences with fastx clipper (http://hannonlab.cshl.edu/fastx_toolkit/), or cutadapt^89^, then mapped to the reference sgRNA library with bowtie2^90^. After filtering to remove multi-aligning reads, read counts were computed for each sgRNA. The RSA algorithm was used to rank genes for significantly enriched sgRNA in the sorted populations compared to control unsorted populations^83^. Genes were ranked according to p-value and candidate genes showing enrichment of at least 4 sgRNA were classified into ‘High’, ‘Moderate’, and ‘Low’ confidence hits, respectively defined by p-values < 10^-5^, < 10^-4^ to >10^-5^, and <10^-3^ to >10^-4^, respectively. To limit false positive hits within moderate and low confidence groups, the genes identified in both HLA-B and HLA-A/B/C screens were prioritised for validation.

### CRISPR/Cas9-Mediated Gene Disruption and Generation of Knockout Clones

Single guide RNA (sgRNA) oligonucleotides (Sigma-Aldrich) were cloned into lentiviral expression vector pKLV-U6gRNA(BbsI)-PGKpuro2ABFP as described (Addgene 50946, a gift from Kosuke Yusa)^91^. For CRISPR/Cas9 mediated gene disruption, cells were first transduced with the Cas9 expression vector pHRSIN-PSFFV-Cas9-PPGK-Blasticidin^27^ or FUCas9Cherry (a gift from Marco Herold, Addgene 70182)^92^, and selected with blasticidin or sorted for mCherry expression respectively. To generate polyclonal populations with targeted gene disruption, cells were subsequently transduced with pKLV-gRNA-PGKpuro2ABFP encoding either gene specific sgRNAs or with a control sgRNA targeting a ‘safe’ genomic location with no annotated function^88^. Efficient functional CRISPR/Cas9 mediated gene disruption of target genes were confirmed by immunoblot. For *BAHD1* knock-out, where immunoblot was unsuccessful due to lack of effective antibodies, Sanger sequencing of the expected DNA break site was performed, with insertion-deletion (indel) frequency estimated by online ICE analysis (https://ice.synthego.com).To generate knockout clones, sgRNAs targeting the gene of interest in parallel with a control targeting a ‘safe’ genomic location were electroporated into Cas9-expressing K-562 cells using the Neon Transfection System (Invitrogen). Cells were selected with puromycin 2μg/mL for 24 hours, beginning 24 hours following electroporation and subsequently sorted for BFP negative single cells into 96-well plates using a BD FACSAria Fusion flow cytometer. Clones were screened by immunoblot and FACS for cell surface MHC-I. Successful clones were verified to be BFP negative and sensitive to puromycin to exclude integration of the sgRNA vector. For *MEN1* complementation, K-562 MEN1 KO clones were infected with pBABE-hygro-MEN1 and selected with hygromycin.

### Virus Production and Transduction

Lentivirus was produced by triple transfection of HEK-293ET cells with a lentiviral transfer vector, and the packaging plasmids psPAX2 and pMD.G at a 0.5:0.35:0.15 ratio. Retrovirus was produced by triple transfection of HEK-293ET cells with a retroviral transfer vector structural pMD1-gag-pol-plasmid and pMD.G envelope plasmid at a 0.75:0.22:0.03 ratio. All transfections were performed using polyethylenimine (PEI). Viral supernatants were collected 60 hours following transfection, filtered through a 0.45 μm filter and added to target cells.

### Antibodies

Flow Cytometry: Alexa Fluor 488 monoclonal mouse anti-human HLA-A,B,C (BioLegend, Cat# 311413; clone W6/32; RRID: AB_493133), APC monoclonal mouse anti-human HLA-A,B,C (BioLegend, Cat# 311410; clone W6/32; RRID: AB_314879), Alexa Fluor 647 monoclonal mouse anti-human HLA-A,B,C (BioLegend, Cat# 311414; clone W6/32; RRID: AB_493135), APC-Vio770 anti-HLA class I Bw6 (Miltenyi Biotec, Cat# 130-099-837; clone REA143; RRID: AB_2652034), APC anti-HLA class I Bw6 (Miltenyi Biotec, Cat# 130-099-845; clone REA143; RRID: AB_2652026), Alexa Fluor 647 mouse anti-mouse MHC class I (H-2Kb) (BioLegend, Cat# 116512; clone AF6-88.5; RRID: AB_492917), FITC Monoclonal rat anti-mouse CD45 (BioLegend, Cat# 103108; clone 30-F11; RRID: AB_312973), APC monoclonal rat anti-mouse CD3 (BioLegend, Cat# 100236; clone 17A2; RRID: AB_2561456), PE monoclonal rat anti-mouse CD8a (BioLegend, Cat# 100708; clone 53-6.7; RRID: AB_312747), FITC anti-human CD9 antibody (BioLegend Cat# 312104; clone HI9a; RRID: AB_2075894), Brilliant Violet 421(TM) anti-human CD13 antibody (BioLegend Cat# 301716; clone WM15; RRID: AB_2562526), Brilliant Violet 785(TM) anti-human CD184 (CXCR4) antibody (BioLegend Cat# 306530; clone 12G5; RRID: AB_2687009), PE/Cyanine7 anti-human CD309 (VEGFR2/KDR) antibody (BioLegend Cat# 393008; clone A16085H; RRID: AB_2783297).

Immunoblot: Monoclonal rabbit anti-MLL1/KMT2A (Carboxy-terminal Antigen) (Cell Signaling Technology, Cat# 14197; clone D6G8N; RRID:AB_2688010), Monoclonal rabbit anti-MLL1/KMT2A (Amino-terminal Antigen) (Cell Signaling Technology, Cat# 14689; clone D2M7U; RRID: AB_2688009), Monoclonal rabbit anti-MLL2/KMT2B (Carboxy-terminal Antigen) (Cell Signaling Technology, Cat# 63735; clone D6X2E; RRID:AB_2737357)), Monoclonal mouse anti-human EZH2 (BD Transduction Laboratories, Cat# 563491; clone 11/EZH2; RRID: AB_2738239), Monoclonal rabbit anti-Tri-Methyl-Histone H3 (Lys27) (Cell Signaling Technology, Cat# 9733; clone C36B11; RRID: AB_2616029), Polyclonal rabbit anti-STAT1 (Merck-Millipore, Cat# 06-501; RRID: AB_310145), Monoclonal rabbit anti-phospho-Stat1 (Tyr701) (Cell Signaling Technology, Cat# 9167; Clone 58D6; RRID: AB_561284), Monoclonal rabbit anti-NF-κB p65 (Cell Signaling Technology, Cat# 8242; clone D14E12; RRID: AB_10859369), Monoclonal rabbit anti-Phospho-NF-κB p65 (Ser536) (Cell Signaling Technology, Cat# 3033; clone 93H1; RRID: AB_331284), Polyclonal rabbit anti-Menin (Abcam, Cat# ab2605; RRID: AB_303203), Monoclonal mouse alpha Tubulin (Invitrogen, Cat# 62204; clone DM1A; RRID: AB_1965960), Polyclonal rabbit anti-LEDGF/p75 (Bethyl, Cat# A300-847; RRID: AB_609466), Polyclonal rabbit anti-LEDGF/p75 (Bethyl, Cat# A300-848; RRID: AB_2171223), Monoclonal mouse anti-PCGF1 (Santa Cruz, Cat# sc-515371; clone E-8; RRID: AB_2721914), Polyclonal rabbit anti-MDA5 (Abcam, Cat# ab79055; RRID: AB_1640683), Monoclonal mouse anti-JunD (Santa Cruz, Cat# sc-271938; clone D-9; RRID: AB_10650101), Monoclonal mouse anti-EED (Merck-Millipore, Cat# 05-1320; Clone AA19; RRID:AB_1586999), Monoclonal mouse anti-MHC class I heavy chain (Origene, Cat# AM33035PU-N; Clone HC10; RRID: AB_2728622), Monoclonal mouse anti-RFX5 (Santa Cruz Biotechnology, Cat# sc-271756; Clone C-3, RRID:AB_10710389), Monoclonal rabbit anti-Histone H2A (Lys119) (Cell Signaling Technology, Clone D27C4; Cat# 8240; RRID:AB_10891618), Monoclonal rabbit anti-SET1A (Cell Signaling Technology, Cat# 61702; Clone D3V9S; RRID:AB_2799614), Monoclonal rabbit anti-SET1B (Cell Signaling Technology, Cat# 44922; Clone D1U5D; RRID:AB_2799275), Monoclonal rabbit anti-AEBP2 (Cell Signaling Technology, Cat# 14129; Clone D7C6X; RRID:AB_2798398), Polyclonal rabbit anti-MTF2 (Proteintech, Cat# 16208-1-AP; RRID:AB_2147370), Monoclonal mouse anti-HSP60 (C-10) (Santa Cruz, Cat# sc-376240; RRID: AB_10986282), Monoclonal rabbit anti-SUZ12 (Cell Signaling Technology, Cat# 3737; Clone D39F6; RRID:AB_2196850).

Chromatin Immunoprecipitation: Monoclonal rabbit anti-Tri-Methyl-Histone H3 (Lys27) (Cell Signaling Technology, Cat# 9733; clone C36B11; RRID: AB_2616029), Polyclonal rabbit anti-Menin (Bethyl, Cat# A300-105A; RRID:AB_2143306), Monoclonal rabbit anti-SUZ12 (Cell Signaling Technology, Cat# 3737; clone D39F6; AB_2196850), Polyclonal rabbit anti-H3K4me3 (Abcam, Cat# ab8580; RRID: AB_306649), Polyclonal rabbit anti-H3K27ac (Abcam, Cat# ab4729; RRID: AB_2118291). Polyclonal rabbit anti-MLL1/KMT2A (Bethyl, Cat# A300-086A; RRID:AB_242510).

CUT&Tag: Polyclonal rabbit anti-H3K4me3 (Abcam, Cat# ab8580; RRID: AB_306649), Monoclonal rabbit anti-Tri-Methyl-Histone H3 (Lys27) (Cell Signaling Technology, Cat# 9733; clone C36B11; RRID: AB_2616029), Monoclonal rabbit anti-Histone H2A (Lys119) (Cell Signaling Technology, Clone D27C4; Cat# 8240; RRID:AB_10891618).

CUT&Run: Polyclonal rabbit anti-MLL1/KMT2A (Bethyl, Cat# A300-086A; RRID:AB_242510).

### Flow Cytometry

Cells were washed in PBS and stained on ice for 20-30 min in PBS plus 2% FCS. After washing in PBS/2% FCS, samples were either resuspended in PBS/2% FCS or fixed in 1% paraformaldehyde (PFA). Data were acquired on a BD LSR Fortessa, BD LSR II or BD FACSymphony and analysed in FlowJo.

### Immunoblotting

Cells were lysed in 1% SDS in 100 mM Tris-HCl pH 8.0 with Roche complete EDTA-free protease inhibitor at room temperature. DNA was fragmented either by sonication or adding Benzonase (Sigma) 1:100. Lysates were heated to 70°C in SDS sample buffer with 50 mM DTT for 10 min, separated by SDS-PAGE, and transferred to PVDF membrane (Millipore). Membranes were blocked in 5% milk in TBS + 0.1% Tween-20 or intercept (TBS) blocking buffer (LI-COR), probed with the indicated antibodies, and reactive bands visualised using West Pico (Thermo Fisher Scientific) or LI-COR system, respectively.

### Isolation and In Vitro Activation of CD8+ T Cells

OT-I TCR (C57BL/6-Tg, female, 6-8 weeks old) transgenic mice were bred in house from Joseph Trapani (Peter MacCallum Cancer Centre). Splenocytes were harvested from the spleen of OT-I mice and stimulated by incubation with 20ng/mL of SIINFEKL (OVA peptide) (Sigma-Aldrich, Cat# S7951; CASRN: 138831-86-4) for 72 hours in media supplemented with IL-2 at 100U/mL (NIH, Cat# Ro-23-6019). Cells were washed to remove peptide then re-cultured in media supplemented with IL-2 for 3 days. CD8+ T cells were confirmed by flow cytometry staining for CD45, CD3 and CD8.

### T-cell Cytotoxicity Assays

#### OVA Peptide Processing and Presentation Assay

Ovalbumin (OVA) expressing mouse SCLC cells (SPC-548-OVA, SPC-545-OVA and RP-48-OVA) were generated by infection with MSCV-OVA-mCherry or MSCV-OVA-GFP. OVA expressing cells were pre-treated as indicated with ethanol/DMSO control, 1μM VTP50469 and/or 3μM EPZ-011989 for 7 days, and with 10ng/mL of mIFN-*γ* for 24h (RP-48-OVA) or 20ng/mL of mIFN-*γ* for 2 hours (SPC-545-OVA). Prior to co-culture set up, cells were washed thoroughly to remove inhibitors and IFN-*γ*.

##### Co-cultures measured by flow cytometry

Pre-treated RP-48-OVA expressing tumour cells (mCherry) were mixed with untreated parental cells (expressing MSCV-GFP control vector) at a 0.75:0.25 ratio (mCherry:GFP) and analysed by flow cytometry at T0 to determine the baseline ratio. Cell mixtures were individually plated in triplicate in 96-well plates with T cells added at indicated effector:target ratios. Co-cultures were incubated at 37°C for 24h and analysed by flow cytometry. Relative depletion of OVA expressing tumour cells were calculated for each effector:target ratio, comparing relative percent of target (OVA expressing) tumour cells remaining to control wells (No T cells). Tumour cells were identified as viable (Fixable Violet negative), CD45 negative population.

##### Co-cultures measured by IncuCyte

Pre-treated SPC-548-OVA expressing tumour cells (GFP positive) were plated in triplicate in flat clear bottom black 96-well plates (Corning) coated with 5 μg/mL of fibronectin (Merck) prior to plating. T cells were added at 2:1 (effector:target) ratio. Plates were incubated for 24 hours in the Incucyte SX5 Live Cells Analysis System at 37°C. Images were acquired every 4 hours measuring green object count (GFP positive cells). Depletion of OVA expressing tumour cells in the presence and absence of T cells was calculated by fold-change of green object count at each time point normalised to T0.

### Measurement of T-cell Cytokine Secretion

1x10^5^ (RP-48-OVA and SPC-545-OVA) cells, pre-treated as indicated in the figure legends, were co-cultured with 2x10^5^ OT-I T cells, in triplicate, in 96-well plates. Supernatant was collected after 24 hours (RP-48-OVA) and 4 days (SPC-545-OVA) of co-culture. TNF and mIFN-*γ* was measured with the BD cytometric bead array Mouse TNF and IFN-*γ* Flex sets, acquired on a BD FACSVerse and analysed using FCAP Array software.

### CellTiter-Glo assay

Cells were plated in 96-well plates in triplicate and allowed to adhere (10^4^ cells per well). Cells were treated with ethanol, 0.5-1μM VTP50469 and/or 1μM EPZ-011989 for 5 days. Following this, cells were lysed and viability measured relative to control using the Cell Titer-Glo assay (Promega) according to manufacturers’ instructions. Cell lysates were read on a bioluminometer (Cytation3).

### qRT–PCR

mRNA was prepared with a Qiagen RNeasy kit, and cDNA synthesis was performed with a SuperScript VILO kit (Life Technologies), per the manufacturers’ instructions. Quantitative PCR analysis was performed on an Applied Biosystems StepOnePlus System or Roche LightCycler 480 Real-Time PCR System with SYBR Green reagents. All samples were assayed in triplicate. Relative expression levels were determined with the ΔCt method and normalized to *GAPDH*.

### RNA-Sequencing

RNA was extracted using the Qiagen RNeasy kit. RNA concentration was quantified using a Qubit Fluorometer (Thermo Fisher Scientific). Libraries were prepared using QuantSeq 3’ mRNA-seq Library Prep kit (Lexogen) and sequenced on the NextSeq500 using 75 bp single end chemistry.

### RNA-Sequencing Analysis

Bcl2fastq (Illumina) was used to perform sample demultiplexing and to convert BCL files generated from the sequencing instrument into FastQ files. Reads were aligned to the human genome (G1k V37) using HiSAT2^93^ and assigned to genes using htseq-count^94^. Differential expression was calculated using DESeq2^95^. Genes with a false discovery rate corrected for multiple testing using the method of Benjamini and Hochberg below 0.05 and a log fold change greater than 1 were considered significantly differentially expressed. Heatmaps were generated in R using pheatmap, volcano plots with EnhancedVolcano, venn diagrams with eulerr and violin with ggplot2.

### Chromatin Immunoprecipitation (ChIP)

20-50x10^6^ cells were cross-linked with 1% formaldehyde (Methanol-free, Thermo Fisher Scientific, Cat# 10751395) for 15 min, or 20 min for Menin ChIP-seq, at room temperature, followed by formaldehyde quenching by adding 0.125 M of glycine. For KMT2A and SUZ12 ChIP-Seq, cells were first fixed with 2mM DSG (Thermo Scientific, Cat# 11836794) for 30 min at room temperature, followed by cross-linking as described for 15 min. Cells were lysed in 1% SDS, 10 mM EDTA, 50 mM Tris-HCl, pH 8.0, and Roche complete EDTA-free protease inhibitor, then sonicated in a Covaris ultrasonicator to achieve a mean DNA fragment size of 500 bp. Samples were diluted 1:10 in modified RIPA buffer (1% Triton X-100, 0.1% deoxycholate, 90 mM NaCl, 10 mM Tris-HCl pH 8.0, and protease inhibitors) and incubated rotating with antibody and Protein A Dynabeads (Life Technologies) for a minimum of 12 h at 4°C. After washing twice with low salt buffer (0.1% SDS, 1% Triton X-100, 20 mM Tris-HCl pH 8.0, 2 mM EDTA, 150 mM NaCl), once with high salt buffer (0.1% SDS, 1% Triton X-100, 20 mM Tris-HCl pH 8.0, 2 mM EDTA, 500 mM NaCl) and once with TE (10 mM Tris pH 8.0, 1 mM EDTA), samples were eluted from beads in elution buffer (1% SDS, 0.1 M NaHCO3) then de-crosslinked overnight at 65°C with RNAse A and 0.2 M NaCl. DNA was purified using MinElute PCR purification kit (Qiagen). Sequencing libraries were prepared from eluted DNA using Rubicon ThruPLEX DNA-seq kit. Libraries were size selected between 200-500bps and sequenced on the NextSeq500 using 75bp single-end chemistry.

### ChIP-reChIP

50x10^6^ cells were cross-linked with 1% formaldehyde (Merck) for 15 min at room temperature, followed by formaldehyde quenching by adding 0.125 M of glycine. Cells were lysed in 0.5% SDS, 10 mM EDTA, 50 mM Tris-HCl pH 8.0, and Roche complete EDTA-free protease inhibitor and sonicated in a Covaris ultrasonicator to achieve a mean DNA fragment size of 500 bp. Samples were diluted 1:10 in modified RIPA buffer (1% Triton X-100, 0.1% deoxycholate, 90 mM NaCl, 10 mM Tris-HCl pH 8.0, and protease inhibitors) then pre-cleared with Protein A Dynabeads (Life Technologies), rotating at 4°C for 2 h. Following this, chromatin was transferred to pre-bound anti-H3K27me3-bead mixture (Protein A Dynabeads) and rotated overnight at 4°C. After incubation, samples were washed twice with low salt buffer (0.1% SDS, 1% Triton X-100, 20 mM Tris-HCl pH8.0, 2 mM EDTA, 150 mM NaCl), twice with high salt buffer (0.1% SDS, 1% Triton X-100, 20 mM Tris-HCl pH 8.0, 2 mM EDTA, 500 mM NaCl), once with TE (10 mM Tris [pH 8.0], 1 mM EDTA), then eluted from beads for 30 min at 37°C in elution buffer 1 (50 mM Tris pH 8.0, 1% SDS, 10 mM DTT). Eluates were diluted 1:20 in modified RIPA buffer, 10% was kept as the primary/single ChIP control, and the remainder was pre-cleared with Protein A Dynabeads (Life Technologies), rotating overnight at 4°C. Samples were transferred to the second pre-bound anti-H3K4me3-bead mixture and rotated again overnight at 4°C. Samples were washed twice with low salt buffer, twice with high salt buffer, once with TE and eluted from beads for 30 min at 65°C in elution buffer 2 (1% SDS, 0.1 M NaHCO3). Eluates were de-crosslinked overnight at 65°C with RNAse A and 0.2 M NaCl. Next, Proteinase K was added to 400 mg/mL, incubating at 60°C for 1 hour. DNA was purified using MinElute PCR purification kit (Qiagen). Sequencing libraries were prepared from eluted DNA using Rubicon ThruPLEX DNA-seq kit. Libraries were size selected between 200-500bps and sequenced on the NextSeq500 using 75bp single-end chemistry.

### CUT&Tag

CUT&Tag was performed as previously described^96^. Nuclei were isolated by permeabilizing 5x10^5^ cells in NE1 Buffer (20 mM HEPES-KOH pH 7.9, 10 mM KCl 0.5 mM spermidine, 0.1% Triton-X100, 20% glycerol, 1x Roche complete EDTA-free protease inhibitor) on ice for 10 minutes, then washed with cold PBS and resuspended in Wash Buffer (20 mM HEPES pH 7.5, 150 mM NaCl, 0.5 mM spermidine, 1 x protease inhibitor cocktail). Nuclei were immobilized on concanavalin A magnetic beads activated in Binding Buffer (20 mM HEPES pH 7.9, 10 mM KCl, 1 mM CaCl_2_, 1 mM MnCl_2_). Bead-bound nuclei were then incubated for 2 hours at room temperature with antibody in Antibody Buffer (Wash Buffer supplemented with 2 mM EDTA and 0.1% BSA). Following incubation, beads were washed twice and incubated for 1 hour at room temperature in 300 Buffer (20 mM HEPES pH 7.5, 300 mM NaCl, 0.5 mM spermidine, 1 x protease inhibitor cocktail) with CUTANA pAG-Tn5 enzyme (EpiCypher). Next, beads were washed twice with 300 Buffer and incubated for 1 hour at 37°C in Tagmentation Buffer (300 Buffer supplemented with 10 mM MgCl_2_). Tagmentation was stopped by the addition of 2x Stop Buffer (300 Buffer supplemented with 40 mM EDTA, 0.2% SDS, 400 μg/mL proteinase K) and incubated at 55°C for 2 hours, then held at 4°C overnight. DNA fragments were collected in the supernatant and purified using MinElute PCR purification kit (Qiagen). To amplify libraries, DNA was mixed with universal i5 primer, a uniquely barcoded i7 primer^97^ and NEBNext HiFi 2X PCR Master Mix. The samples were amplified using the following cycling conditions: 72°C for 5 min (gap filling); 98°C for 30 s; 14 cycles of 98°C for 10 s and 63°C for 10 s; final extension at 72°C for 1 min and hold at 8 °C. Post-PCR cleanup was performed using 1x AMPure XP beads (Beckman Coulter), then sequenced on the NextSeq500 using 75 bp paired-end chemistry.

### CUT&RUN

CUT&RUN was performed as previously described^98^. 5x10^5^ cells were washed twice in PBS and resuspended in Wash Buffer (20 mM HEPES pH 7.5, 150 mM NaCl, 0.5 mM spermidine, 1 x Roche complete EDTA-free protease inhibitor), followed by immobilization on concanavalin A magnetic beads activated in Binding Buffer (20 mM HEPES pH 7.9, 10 mM KCl, 1 mM CaCl_2_, 1 mM MnCl_2_). Bead-bound cells were then incubated with antibody in Antibody Buffer (Wash Buffer supplemented with 2 mM EDTA and 0.05% Digitonin) for 2 hours at room temperature. Following incubation, beads were washed twice with Digitonin Buffer (Wash Buffer supplemented with 0.05% Digitonin) and incubated 1 hour at room temperature in Digitonin Buffer with pAG-MNase enzyme (Cell Signaling). Next, beads were washed twice with Digitonin Buffer and MNase was activated by adding CaCl_2_ (50 μL of Digitonin Buffer supplemented with 4mM CaCl_2_) and incubated for 1 hour at 37°C. DNA digestion was stopped by the addition of 2X Stop Buffer (340 mM NaCl, 20 mM EDTA, 4 mM EGTA, 0.1% Digitonin, 100 μg/mL RNAse A, 50 μg/mL Glycogen) and incubated at 37°C for 1 hour. DNA fragments were collected in the supernatant and purified using MinElute PCR purification kit (Qiagen). Sequencing libraries were prepared using Rubicon ThruPLEX DNA-seq kit, followed by sequencing on the NextSeq500 using 75bp paired-end chemistry.

### ChIP, CUT&Tag and CUT&RUN -Sequencing Analysis

Bcl2fastq (Illumina) was used to perform sample demultiplexing and to convert BCL files generated from the sequencing instrument into FastQ files. ChIP-seq data reads were aligned to the human genome (GRCh37) combined with fly genome (BDGP5) with BWA-MEM^99^. Peak calling was performed with MACS2^100^ with default parameters, plus -B for additional bedgraph output. CUT&Tag and CUT&RUN, reads were aligned with bowtie2^89^ and peaks calls were from MACS2 output, both as implemented via the CUT&RUN tools pipeline. For all experiments, reads in peaks were tabulated using getCounts from the chromVAR package in R which were then used for calculation of LFC. deepTools^101^ was used to normalize bams by RPGC to generate bigwig files subsequently used for heatmaps and gene profile plots. Log fold change was calculated using read normalised counts summed for all peaks occurring within a gene body and within 1kb of the promoter, comparing knockout to control samples. Bivalent genes were classified as genes with peaks called for both H3K4me3 and H3K27me3 within 2kbp of the TSS. The reduced/increased KMT2A analysis was performed using all peaks which had at least 1 read per million (RPM) in the KMT2A CUT&RUN and H3K4me3 ChIPseq samples. Reduced/increased KMT2A groups were defined as peaks within 2kbp of the TSS showing either an increase or reduction of 1RPM in the KMT2A CUT&RUN in *MEN1* knockout cells compared to control. H3K4me3 and H3K27me3 occupancy at sites with increased KMT2A in *MEN1* knockout cells were defined across the same regions. Genome browser plots were generated with Spark^102^ using bedgraph files output from MACS peak calling.

### Statistics and Reproducibility

Statistical analysis was carried out using GraphPad Prism 9. Details of statistical analysis and significance values are specified in the figures and figure legends, non-significant data were not annotated. Data were reported as mean ± SD, SEM or independent replicates shown as individual data points.

Representative gating strategy for flow cytometry data is shown in Supplementary Figure 1. Flow cytometry data were experimentally reproduced three times, unless indicated otherwise in figure legends. This data is shown in Supplementary Figure 2 & 3. Immunoblots were experimentally reproduced at least two times.

### sgRNA and primers sequences

The sequences for sgRNA and primers are included in Supplementary Tables 6 & 7.

## Extended Data

## Supplementary Material

Inventory of Supporting Information

Source Data Extended Data Fig. 1

Source Data Extended Data Fig. 2

Source Data Extended Data Fig. 2

Source Data Extended Data Fig. 3

Source Data Extended Data Fig. 3

Source Data Extended Data Fig. 4

Source Data Extended Data Fig. 4

Source Data Extended Data Fig. 5

Source Data Extended Data Fig. 6

Source Data Extended Data Fig. 6

Source Data Extended Data Fig. 7

Source Data Extended Data Fig. 9

Source Data Extended Data Fig. 9

Source Data Extended Data Fig. 10

Source Data Fig. 1

Source Data Fig. 2

Source Data Fig. 2

Source Data Fig. 3

Source Data Fig. 3

Source Data Fig. 4

Source Data Fig. 5

Source Data Fig. 5

Source Data Fig. 6

Source Data Fig. 7

Source Data Fig. 8

Source Data Fig. 8

Supplementary Figures 1-3.

Supplementary Tables 1-7

## Data Availability

ChIP-seq, RNA-seq, CUT&Tag and CUT&Run data that support the findings of this study have been deposited in the Gene Expression Omnibus (GEO) under the accession code GSE181829. Human embryonic stem cell (hESC) line H9 ChIP-seq data was used from GEO:GSE96336 and GEO:GSE96353, EZH2 null H9 hESC RNAs-seq data from GEO: GSE76626, and human induced pluripotent stem cell line iPS-20b ChIP-seq data from GEO:GSM772844 and GEO:GSM772847. All other data supporting the findings of this study are available from the corresponding authors on reasonable request.
