# Supplementary material for: Targeting Menin disrupts the KMT2A/B and polycomb balance to paradoxically activate bivalent genes
Source: Nat Cell Biol. Author manuscript; Available in PMC 2023 Feb 17. (PMC7614190; doi:10.1038/s41556-022-01056-x)
Supplement: Inventory of Supporting Information [file EMS157168-supplement-Inventory_of_Supporting_Information.docx]

Inventory of Supporting Information

**Manuscript #:** NCB-D46667

**Corresponding author name(s):** Mark A. Dawson & Marian L. Burr

| Please complete each of the Inventory Tables below to outline your Extended Data and Supplementary Information items.  There are four sections:   - *Extended Data* - *Supplementary Information: Flat Files* - *Supplementary Information: Additional Files* - *Source Data*   Each section includes specific instructions. Please complete these tables as fully as possible. We ask that you avoid using spaces in your file names, and instead use underscores, i.e.: Smith_ED_Fig1.jpg not Smith ED Fig1.jpg  Please note that titles and descriptive captions will only be lightly edited, so please ensure that you are satisfied with these prior to submission.  If you have any questions about any of the information contained in this inventory, please contact the journal. |
| --- |
|  |

1. **Extended Data**

**Complete the Inventory below for all Extended Data figures.**

- Keep Figure Titles to one sentence only
- Upload your files as ‘Figure Files’ in our Manuscript Tracking system
- File names should include the Figure Number. i.e.: *Smith_ED_Fig1.jpg*
- Please be sure to include the file extension in the Filename. Note that Extended Data files must be submitted as .jpg, .tif or .eps files *only*, and should be approximately 10MB
- All Extended Data figure legends must be provided in the Inventory below and should not exceed 300 words each *(if possible)*
- Please include Extended Data *ONLY* in this table

| Figure # | Figure title  One sentence only | Filename  This should be the name the file is saved as when it is uploaded to our system. Please include the file extension. i.e.: *Smith_ED_Fig1.jpg* | Figure Legend  If you are citing a reference for the first time in these legends, please include all new references in the main text Methods References section, and carry on the numbering from the main References section of the paper. If your paper does not have a Methods section, include all new references at the end of the main Reference list. |
| --- | --- | --- | --- |
| Extended Data Fig. 1 | MHC-I genes harbour bivalent H3K4me3 and H3K27me3 modifications. | Extended_Figure1.eps | (a) Genomic snapshots of MHC-I genes showing H3K4me3 and H3K27me3 CUT&Tag in K-562 and ChIPseq in Neuroblastoma KELLY cell lines. The K-562 tracks are also shown in the control cells in Fig. 2h and H3K27me3 control cells in Fig. 6f. (b & c) Cell surface MHC-I in K-562 (left) and KELLY (right) cells following treatment with EPZ-011989 and (c) ± 10ng/mL IFN-γ (48h K-562, 24h KELLY). (d) Genomic snapshots of MHC-I genes showing ChIP-seq for H3K4me3, H3K27me3 and H3K27ac in KELLY cells treated with EtOH (control) or EPZ-011989 ± IFN-γ. (e & f) ChIP re-ChIP-seq of single H3K27me3, single H3K4me3 and reChIP (H3K27me3 and H3K4me3) in K-562 cells. (e) Genomic snapshots of bivalent MHC-I genes. (f) Heatmaps show bivalent genes -3kb TSS/ +3kb TES, with genomic regions ordered by H3K27me3 read density in the single H3K27me3 ChIP sample. (b/c) show representative plots from 3 experiments (Supplementary Figure 3). |
| Extended Data Fig. 2 | Genome wide CRISPR/Cas9 screen identifies regulators of MHC Class I expression. | Extended_Figure2.eps | (a) Cell surface MHC-I, pan-HLA-A,B,C (top panel) and HLA-B (bottom) specific antibodies, in K-562 Cas9 cells treated with the indicated IFN-γ doses for 24h. (b) K-562 cells stably expressing Cas9 were mutagenised by infection with a pooled lentiviral sgRNA library and treated with 1ng/mL IFN-γ for 24h prior to FACS sorting. Rare MHC-I high cells were enriched by 2 successive rounds of FACS sorting for mCherry positive (containing sgRNA vector) MHC-I positive cells. FACS dot plots and histograms show MHC-I expression in unsorted, post sort 1 and post sort 2 in K-562 Cas9 cells transduced with the CRIPSR sgRNA library and sorted with either pan-HLA-A,B,C (top panels) or HLA-B (bottom panels) specific antibodies. (c) Table depicting correlation between CRISPR gene effect scores (Fig. 1e) for top 20 shared *EZH2* and *EED* co-dependent genes calculated from combined CRISPR survival screens in 990 cancer cell lines in Cancer Dependency Map (<https://depmap.org/portal/>)^31, 32^. Table indicates Pearson correlation coefficients. (d & e) Immunoblots of K-562 Cas9 cells transduced with control and (d) *MTF2* or (e) *AEBP2* sgRNA. (f) H3K4me3 and H3K27me3 CUT&Tag. Genomic snapshots of bivalent MHC-I genes in K-562 cells transduced with control, *MTF2* and *AEBP2* sgRNA. The H3K4me3 control tracks are the same control tracks in Fig. 7c. (g) Cell surface MHC-I in K-562 Cas9 cells transduced with control or *BAHD1-*specific sgRNAs and treated with 10ng/mL IFN-γ for 48h. Representative plots from 3 experiments (Supplementary Figure 3). (h) Knockout scores of individual sgRNA targeting *BAHD1* measured using Synthego Performance Analysis, Interference of CRISPR editing (ICE) Analysis. |
| Extended Data Fig. 3 | Loss of PRC1 drives derepression of bivalent genes. | Extended_Figure3.eps | (a) Immunoblot of K-562 Cas9, *PCGF1* KO and *EED* KO cells ± 10ng/mL IFN-γ (40h). (b & c) Cell surface MHC-I in K-562 Cas9 cells transduced with either control or *PCGF1* sgRNA. (c) Bars show mean percentage of MHC-I expression from 3 experiments, indicated by points. Unpaired two-tailed t-test, p=0.0295. (d) qRT-PCR for MHC-I genes in K-562 Cas9 cells transduced with control or *PCGF1* sgRNA. Bars indicate mean ± s.d. of technical triplicates from a representative experiment. (e) Cell surface MHC-I in *EED* KO cells transduced with control or *MTF2* sgRNA. Representative plot from 3 experiments (Supplementary Figure 3). (f) Immunoblot of K-562 Cas9 and *EED* KO cells transduced with control and *PCGF1* sgRNA. (g & h) Cell surface MHC-I in K-562 Cas9 cells transduced with *RING1A* and/or *RING1B* sgRNA, following treatment with 10ng/mL IFN-γ for 36h. (h) Bars show mean fold change in MFI from 3-5 experiments, indicated by points. Unpaired two-tailed t-test, p-values are indicated. (i) Immunoblot of K-562 Cas9 cells transduced with indicated sgRNA. (j) Genomic snapshots of bivalent MHC-I genes showing H3K4me3, H3K27me3 and H2AK119Ub CUT&Tag in K-562 Cas9 (control), *EED* KO and *PCGF1* KO cells. The H3K4me3 and H3K27me3 control tracks are the same control tracks in Fig. 6f. (k) H2AK119Ub CUT&Tag in K-562 cells transduced with control or *MTF2* sgRNA. Heatmaps show bivalent genes -3kb TSS/ +3kb TES. Genomic regions are ordered by H2AK119Ub read density in the control sample. |
| Extended Data Fig. 4 | Depletion of Menin or LEDGF enhances basal and IFN-γ induced bivalent MHC-I gene expression. | Extended_Figure4.eps | (a & b) Cell surface MHC-I in K-562 Cas9 cells transduced with either control, *MEN1* or *PSIP1* sgRNA. (b) Bars show mean percentage of MHC-I expression from 3 experiments, indicated by points. Unpaired two-tailed t-test, significant changes are indicated, p=0.0356. (c) qRT-PCR for MHC-I genes in K-562 Cas9 cells transduced with control or *MEN1* sgRNA. Bars indicate mean ± s.d. of technical triplicates from a representative experiment. (d) Immunoblot of K-562 Cas9, *MEN1* KO and *PSIP1* KO cells ± 10ng/mL IFN-γ for 40h. (e) Cell surface MHC-I in K-562 Cas9 cells transduced with control or indicated sgRNA targeting *MEN1.* (f & g) Immunoblots of (f) K-562 Cas9 cells transduced with control sgRNA or sgRNA targeting *MEN1*, (g) *MEN1* KO cells ± *MEN1* cDNA. (h & i) JunD is not required for enhanced MHC-I expression following *MEN1* KO. K-562 Cas9 and *MEN1* KO cells transduced with control or *JunD* sgRNA and analysed by (h) flow cytometry, following treatment with 10ng/mL IFN-γ for 48h, and (i) immunoblot. (h) Shows representative plots from 3 experiments (Supplementary Figure 3). |
| Extended Data Fig. 5 | Pharmacological targeting of Menin-KMT2A/B and PRC2 similarly augment IFN-γ induced MHC-I expression in MHC-I low cancers and enhance T cell mediated killing. | Extended_Figure5.eps | (a) qRT-PCR analysis in K-562 cells treated ± 500nM VTP50469. Bars indicate mean ± s.d. of technical triplicates. (b) MI-503, a chemically distinct inhibitor of the Menin-MLL interaction, also enhanced IFN-γ induced MHC-I expression. Cell surface MHC-I in K562 Cas9 cells pre-treated with 500nM MI-503 and 10ng/mL IFN-γ (48h). Representative plot from 3 experiments (Supplementary Figure 3). (c) Cell surface MHC-I in cells treated with DMSO or 3µM EPZ-011989 and 10ng/mL IFN-γ (24h SCLC, 40h KELLY), (VTP50469 treatment: Figure 4a). Representative plots from independent experiments (n=2 SCLC, n=3 KELLY (Supplementary Figure 3)). (d) Cell surface MHC-I expression in SCLC cells treated with DMSO, 1µM VTP50469 or 3µM EPZ-011989 and 10ng/mL IFN-γ for 24h. Representative plots from 2 experiments (Supplementary Figure 3). (e) Scatter plot indicating *MEN1* and *EED* CERES gene perturbation effects for neuroblastoma cell lines evaluated in combined CRISPR screens in DepMap (DepMap 21Q2 Public+Score, CERES (<https://depmap.org/portal/>)^31, 32^. (f) Flow cytometry analysis of RP-48-OVA cells pre-treated with DMSO or 1µM VTP50469 and 10ng/mL murine IFN-γ (24h) prior to co-culture with OVA antigen-specific OT-I T cells at the indicated effector:target (E:T) ratios. Bars indicate mean percent remaining mCherry positive (RP-48-OVA) cells compared to no T-cell control from 3 independent replicates, indicated by points. Unpaired two-tailed t-tests compared to respective DMSO controls. Significant changes are indicated. (g) Cytometric Beads Array (CBA) assay for mIFN-γ following 24h co-culture of RP-48-OVA cells pre-treated with DMSO or 1µM VTP50469 and 10ng/mL murine IFN-γ (24h) prior to co-culture with OVA antigen-specific OT-I T cells at a 2:1 (E:T) ratio. Bars show mean expression from 2-3 independent replicates, indicated by points. Unpaired two-tailed t-test, p=0.01. (h) Cell surface MHC-I in SPC-545-OVA cells pre-treated with DMSO, 1µM VTP50469 and/or 3µM EPZ-011989, and 1ng/mL murine IFN-γ (24h). Representative plot from 2 experiments (Supplementary Figure 3). (i) CBA assay for mIFN-γ and TNF following 4 days co-culture of pre-treated SPC-545-OVA cells (DMSO,  1µM VTP50469 and/or 3µM EPZ-011989 and 2h 20ng/mL mIFN-γ) with OVA antigen-specific OT-I T cells at a 2:1 (E:T) ratio. Bars show mean expression from 3 independent replicates, indicated by points. Unpaired two-tailed t-test compared to respective DMSO +mIFN-γ controls. Significant changes are indicated. |
| Extended Data Fig. 6 | Targeting Menin drives expression of bivalent genes independently of interferon and NFkB signalling. | Extended_Figure6.eps | (a & b) Immunoblot in K-562 *EED* KO cells depleted of (a) *MEN1, PSIP1* or (b) *PCGF1,* then transduced with indicated sgRNA. (c) Immunoblot in K-562 Cas9 and *EED* KO cells transduced with indicated sgRNA and treated ± 10ng/mL IFN-γ for 48h. (d-h) K-562 *EED* KO cells depleted of *MEN1, PSIP1* or *PCGF1* and transduced with indicated sgRNA, analysed by (d & f) flow cytometry, and (e, g & h) immunoblot. (i) Immunoblot in K-562 Cas9 and *EED* KO cells transduced with indicated sgRNA and treated ± 20ng/mL TNF-α for 48h. (j) Cell surface MHC-I expression in K-562 *EED* KO cells transduced with control or *PCGF1* sgRNA and treated ± 25ng/mL IFN-γ for 24h. (d), (f) and (j) each show representative plots from 3 experiments (Supplementary Figure 3). |
| Extended Data Fig. 7 | Loss of Menin alleviates repression of bivalent genes. | Extended_Figure7.eps | (a) Volcano plot showing Log_2_FC gene expression from RNA-seq data in K-562 cells expressing *MEN1* sgRNA compared with control sgRNA. Selected MHC class I genes are labelled. Two-sided Wald test, p-values adjusted for multiple testing.  (b) Venn diagram depicting overlap in genes down-regulated (p-adj <0.05 and fold-change >2) after CRISPR deletion of *MEN1*, *PSIP1* or *EED*. (c) Venn diagrams depicting overlap in genes up and down-regulated (p-adj <0.05 and fold-change >2) after CRISPR deletion of *MEN1* or *PSIP1* or 500nM VTP50469 treatment. (d) Pharmacological inhibition of Menin-KMT2A/B induces genome wide displacement of Menin from chromatin. Menin ChIP-seq in K-562 cells treated for 48h with DMSO or 1µM VTP50469. Average profile plots (top) and heatmaps (bottom) of Menin occupied sites -3kb TSS/ +3kb TES. Genomic regions are ordered by Menin occupancy in control sample. (e & f) Immunoblots of K-562 Cas9 (control), *MEN1* KO, *PSIP1* KO and *PCGF1* KO cells. (g) Genomic snapshots of MHC-I genes from SUZ12 ChIP-seq data in K-562 Cas9 control and *MEN1* KO cells. (h) Genomic snapshots of H3K4me3, SUZ12 ChIP-seq and H3K27me3 CUT&Tag in K-562 Cas9 control and *MEN1* KO cells. |
| Extended Data Fig. 8 | Targeting Menin potentiates bivalent gene derepression in human pluripotent stem cells. | Extended_Figure8.eps | (a) RNA-seq in H9 hESCs treated with DMSO, 1µM VTP50469 and/or 3µM EPZ-011989 for 5 days. Heatmap includes bivalent genes significantly up- or down-regulated in combination Menin/EZH2 inhibitor treated cells compared to DMSO control (p-adj <0.05 and Log_2_FC >1 or <-1). (b & c) RNA-seq in wildtype (WT), EZH2null (*EZH2*-/-) and EZH2-complemented EZH2null (*EZH2*-/-+*EZH2*) H9 hESCs (GEO: GSE76626)^60^. (b) Box-plots include the top upregulated bivalent genes in combination Menin + EZH2 inhibitor treated H9 hESCs (Log_2_FC >4 compared to DMSO control) and depict median Log_2_FC in expression in EZH2null or EZH2-complemented H9 hESCs compared to wildtype control^60^. Whiskers represent the minimum and maximum, the box represents the interquartile range, and the centre line represents the median. (c) Heatmap shows Log_2_FC in expression of selected germ layer specific genes in either EZH2null or EZH2-complemented H9 hESCs compared to wildtype control^60^. (d) Heatmap shows Log_2_FC in expression of selected germ layer specific genes in H9 hESCs treated with 1µM VTP50469 and/or 3µM EPZ-011989 compared to DMSO control. (e & f) ChIP-seq in H9 hESCs. Genomic snapshots showing data from (e) KMT2A, and (f) KMT2A, H3K4me3 (GEO: GSE96336) and H3K27me3 (GEO: GSE96353)^84^. |
| Extended Data Fig. 9 | KMT2A/B is required for basal MHC-I expression. | Extended_Figure9.eps | (a) Cell surface MHC-I in K-562 Cas9 cells transduced with *KMT2A* or *KMT2B* sgRNA compared to control sgRNA and treated with 10ng/mL IFN-γ for 48h. Bars show mean percentage of MHC-I expression from 3 experiments, indicated by points. Unpaired two-tailed t-test compared to control sgRNA. Significant changes are indicated, p<0.0001. (b & c) Immunoblots in K-562 Cas9 and, (b) *KMT2B* KO cells, (c) *KMT2A* KO ± *KMT2B* KO cells. (d) Cell surface MHC-I in K-562 *KMT2B + PCGF1* KO cells transduced with indicated sgRNA and treated for 5 days with DMSO, 1µM VTP50469 or 3µM EPZ-011989. Representative plot from 3 experiments (Supplementary Figure 3). (e) Genomic snapshots of H3K4me3 CUT&Tag in K-562 Cas9 and *KMT2A/B* KO cells treated ± EPZ-011989. The EZH2i treated (no IFN-γ) track is also shown in 8g. (f) Immunoblots in K-562 Cas9, *MEN1* KO and *KMT2A* KO cells. (g-i) Genomic snapshots of K-562 Cas9, and *MEN1* KO cells (g & h) H3K4me3 ChIP-seq and KMT2A CUT&RUN. The H3K4me3 tracks are also shown in Extended Data Fig. 7h. (i) KMT2A CUT&RUN. |
| Extended Data Fig. 10 | KMT2A/B is dispensable for MHC enhanceosome driven activation. | Extended_Figure10.eps | (a) Schematic overview of cis-regulatory elements in the MHC-I promoter. NLRC5 forms an enhanceosome with the RFX (regulatory factor X) complex, made up of RFX5, RFXANK and RFAXP (RFX-associated ankyrin-containing protein); CREB (cAMP-responsive-element-binding); and NFY (nuclear transcription factor Y), which bind the SXY-molecule to activate transcription of MHC-I. (b) Immunoblot of K-562 Cas9 cells transduced with control and *RFX5* sgRNA. (c) IFN-γ time-course in K-562 Cas9 and indicated KO cells treated with 3µM EPZ-011989 and 25ng/mL IFN-γ for the indicated time points. (d) Immunoblot of K-562 Cas9 and *KMT2A/B* KO cells transduced with control, *SETD1A* and/or *SETD1B* sgRNA. |

***Delete rows as needed to accommodate the number of figures (10 is the maximum allowed).***

1. **Supplementary Information:**
2. **Flat Files**

**Complete the Inventory below for all additional textual information and any additional Supplementary Figures, which should be supplied in one combined PDF file.**

- **Row 1:** A combined, flat PDF containing any Supplementary Text, Discussion, Notes, Additional Supplementary Figures, Supplementary Protocols, simple tables, and all associated legends. Only one such file is permitted.
- **Row 2:** Nature Research’s Reporting Summary; if previously requested by the editor, please provide an updated Summary, fully completed, without any mark-ups or comments. **(Reporting Summaries are not required for all manuscripts.)**

| Item | Present? | Filename  This should be the name the file is saved as when it is uploaded to our system, and should include the file extension. The extension must be .pdf | A brief, numerical description of file contents.  i.e.: *Supplementary Figures 1-4, Supplementary Discussion, and Supplementary Tables 1-4.* |
| --- | --- | --- | --- |
| Supplementary Information | Yes | Supplementary_Flow_Cytometry_Figures.pdf | Supplementary Figures 1-3. |
| Reporting Summary | Yes | NCB_Reporting_Summary_October.pdf |  |
| Peer Review Information | No | *OFFICE USE ONLY* |  |

1. **Additional Supplementary Files**

**Complete the Inventory below for all additional Supplementary Files that cannot be submitted as part of the Combined PDF.**

- Do not list Supplementary Figures in this table (see section 2A)
- Where possible, include the title and description within the file itself
- Spreadsheet-based tables & data should be combined into a workbook with multiple tabs, not submitted as individual files.
- Compressed files are acceptable where necessary. ZIP files are preferred.
- Please note that the *ONLY* allowable types of additional Supplementary Files are:

| - Supplementary Tables | - Supplementary Audio | - Supplementary Videos | - Supplementary Software |
| --- | --- | --- | --- |
| - Supplementary Data, for example: raw NMR Data, Cryo-EM Data, Computational Data, Crystallographic Data, etc. | | | |

| Type | Number  If there are multiple files of the same type this should be the numerical indicator. i.e. “1” for Video 1, “2” for Video 2, etc. | Filename  This should be the name the file is saved as when it is uploaded to our system, and should include the file extension. i.e.: *Smith_ Supplementary_Video_1.mov* | Legend or Descriptive Caption  Describe the contents of the file |
| --- | --- | --- | --- |
| Supplementary Table | 1-7 | Supplementary_Tables.xlsx | Supplementary Tables 1-7.  Supplementary Tables 1-3: CRISPR Screen results. Related to Figure 1. Supplementary Table 4: Gene lists for RNA-seq data. Related to Figure 6 and Extended Data Figure 7. Supplementary Table 5: Gene list intersection of CRISPR Screen and RNA-seq results. Supplementary Tables 6 & 7: Primer sequences. |

***Add rows as needed to accommodate the number of files.***

1. **Source Data**

**Complete the Inventory below for all Source Data files.**

- Acceptable types of Source Data for Main Figures and Extended Data Figures are:
  - Statistical Source Data
    - Plain Text (ASCII, TXT) or Excel formats only
    - One file for each relevant Figure, containing all source data
  - Full-length, unprocessed Gels or Blots
    - JPG, TIF, or PDF formats only
    - One file for each relevant Figure, containing all supporting blots and/or gels
- ‘Source Data’ is only allowed for Main Figures and Extended Data Figures.
  - Include Unprocessed Gels or Blots for Supplementary Figures as additional Supplementary Figures.
  - Include Statistical Source Data for Supplementary Figures as ‘Supplementary Data’ files and list them in section 2B.
  - Please see [this example of Source Data](https://www.nature.com/articles/s41591-019-0505-4) in a publication.

| Parent Figure or Table | Filename  This should be the name the file is saved as when it is uploaded to our system, and should include the file extension. i.e.: *Smith_SourceData_Fig1.xls,* or *Smith_ Unmodified_Gels_Fig1.pdf* | Data description  i.e.: Unprocessed Western Blots and/or gels, Statistical Source Data, etc. |
| --- | --- | --- |
| Source Data Fig. 1 | Source_Fig1.xlsx | Source Data |
| Source Data Fig. 2 | Source_Fig2.xlsx,  Source_blots_Fig2.pdf | Source Data, Unprocessed Western Blots |
| Source Data Fig. 3 | Source_Fig3.xlsx, Source_blots_Fig3.pdf | Source Data, Unprocessed Western Blots |
| Source Data Fig. 4 | Source_Fig4.xlsx | Source Data |
| Source Data Fig. 5 | Source_Fig5.xlsx, Source_blots_Fig5.pdf | Source Data, Unprocessed Western Blots |
| Source Data Fig. 6 | Source_Fig6.xlsx | Source Data |
| Source Data Fig. 7 | Source_Fig7.xlsx | Source Data |
| Source Data Fig. 8 | Source_Fig7.xlsx,  Source_blots_Fig8.pdf | Source Data, Unprocessed Western Blots |
| Source Data Extended Data Fig. 1 | Source_ED_Fig1.xlsx, | Source Data |
| Source Data Extended Data Fig. 2 | Source_ED_Fig2.xlsx,  Source_blots_ED_Fig2.pdf | Source Data, Unprocessed Western Blots |
| Source Data Extended Data Fig. 3 | Source_ED_Fig3.xlsx, Source_blots_ED_Fig3.pdf | Source Data, Unprocessed Western Blots |
| Source Data Extended Data Fig. 4 | Source_ED_Fig4.xlsx, Source_blots_ED_Fig4.pdf | Source Data, Unprocessed Western Blots |
| Source Data Extended Data Fig. 5 | Source_ED_Fig5.xlsx | Source Data |
| Source Data Extended Data Fig. 6 | Source_ED_Fig6.xlsx, Source_blots_ED_Fig6.pdf | Source Data, Unprocessed Western Blots |
| Source Data Extended Data Fig. 7 | Source_blots_ED_Fig7.pdf | Unprocessed Western Blots |
| Source Data Extended Data Fig. 8 |  |  |
| Source Data Extended Data Fig. 9 | Source_ED_Fig9.xlsx, Source_blots_ED_Fig9.pdf | Source Data, Unprocessed Western Blots |
| Source Data Extended Data Fig. 10 | Source_blots_ED_Fig10.pdf | Unprocessed Western Blots |
