# Supplementary material for: Targeting Menin disrupts the KMT2A/B and polycomb balance to paradoxically activate bivalent genes
Source: Nat Cell Biol. Author manuscript; Available in PMC 2023 Feb 17. (PMC7614190; doi:10.1038/s41556-022-01056-x)

Source Data: Western blot gels

Western blot images. Extended Data Figure 3

Extended Data Fig. 3a

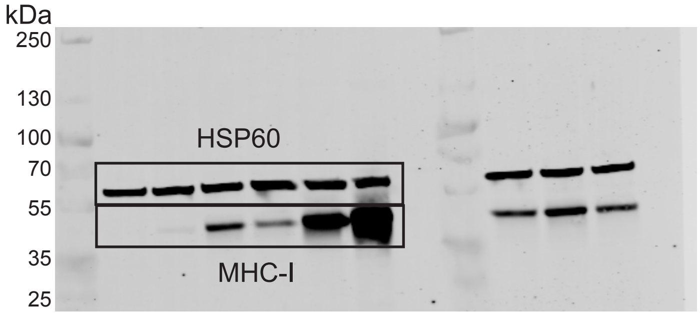

Extended Data Fig. 3f

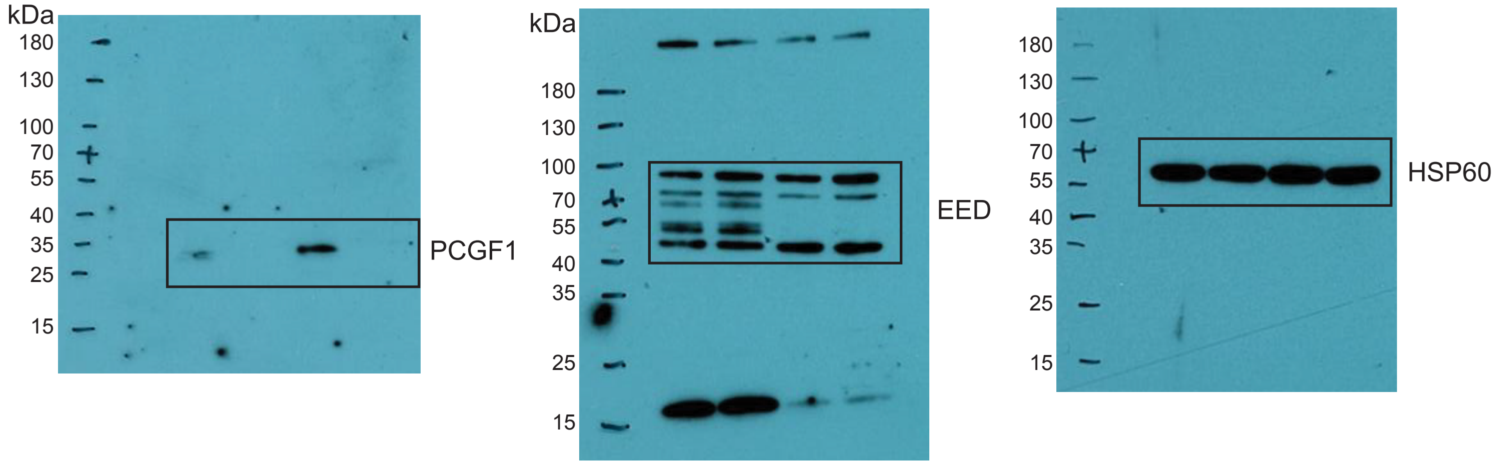

Extended Data Fig. 3i

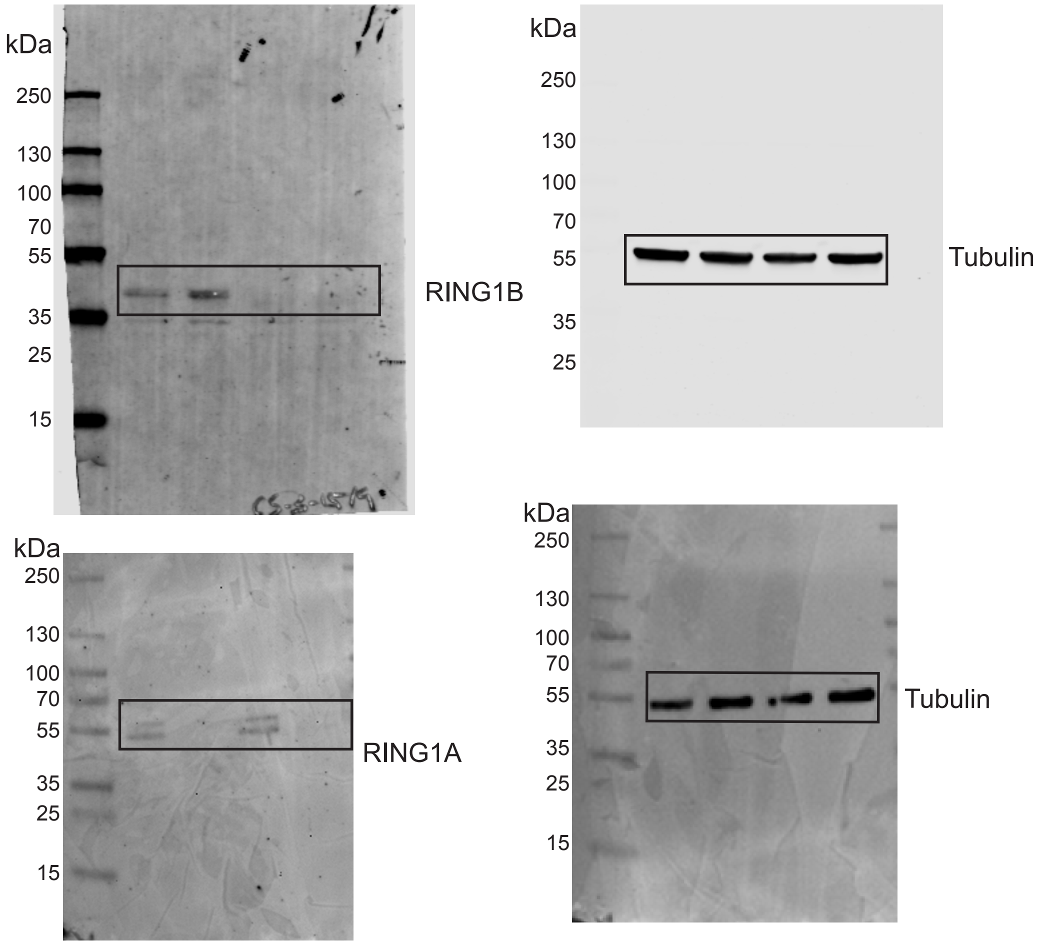

Supplement: Source Data Extended Data Fig. 3 [file EMS157168-supplement-Source_Data_Extended_Data_Fig__3.pdf]
