# Supplementary material for: Targeting Menin disrupts the KMT2A/B and polycomb balance to paradoxically activate bivalent genes
Source: Nat Cell Biol. Author manuscript; Available in PMC 2023 Feb 17. (PMC7614190; doi:10.1038/s41556-022-01056-x)

## Source Data: Western blot gels

**Western blot images. Extended Data Figure 6**

Extended Data Fig.6a

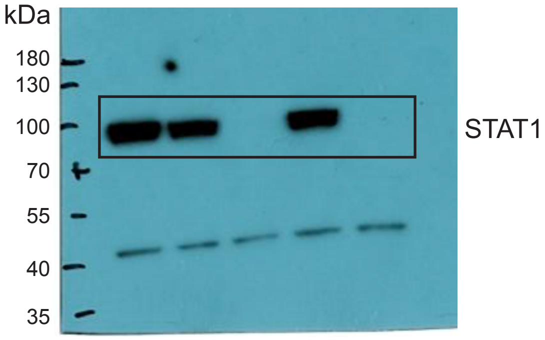


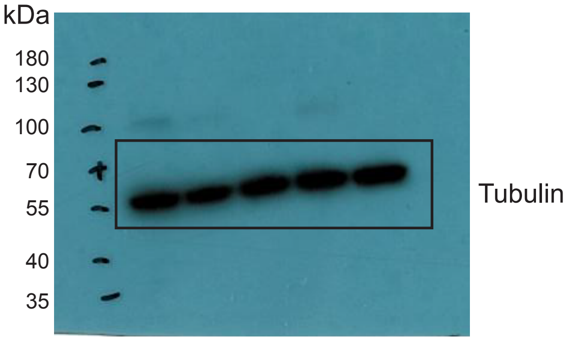


Extended Data Fig.6b

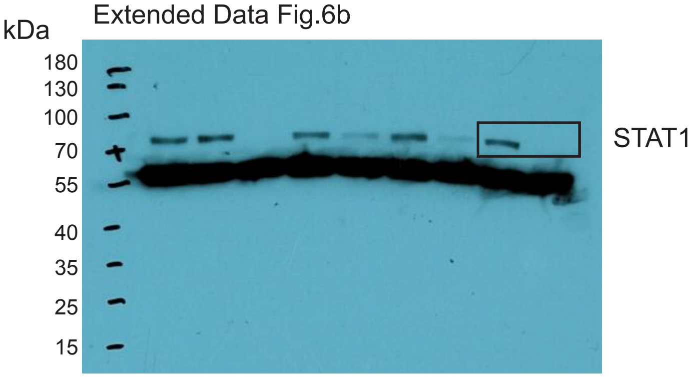


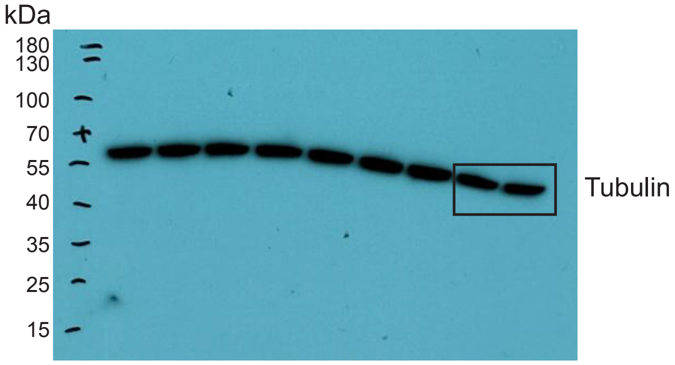


Extended Data Fig.6c

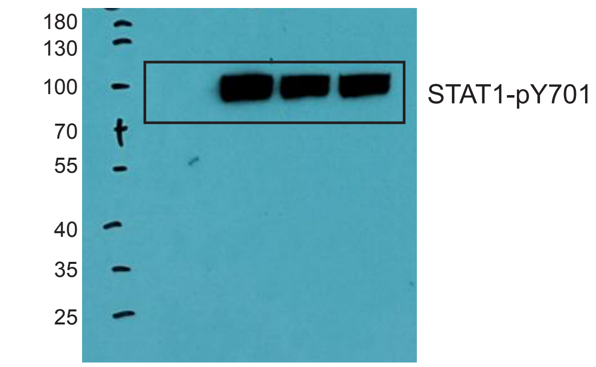


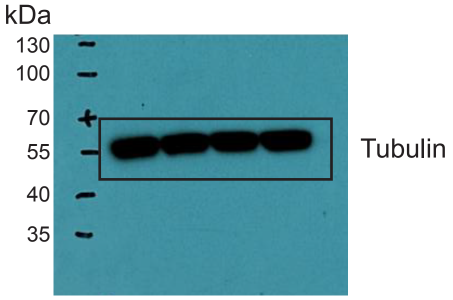


Extended Data Fig.6e

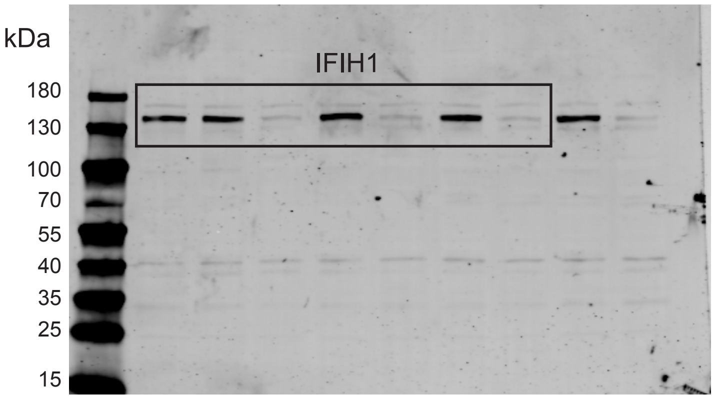


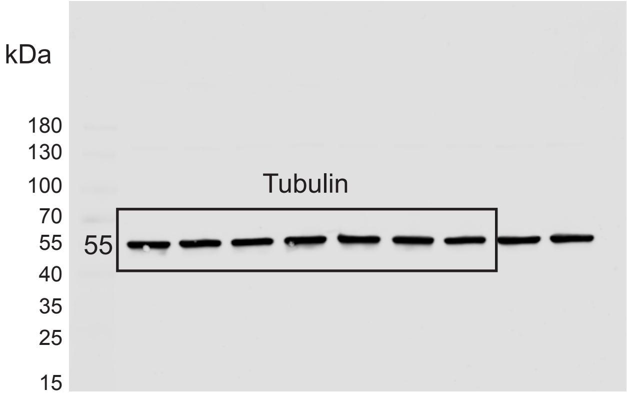

**Western blot images. Extended Data Figure 6**

Extended Data Fig.6g

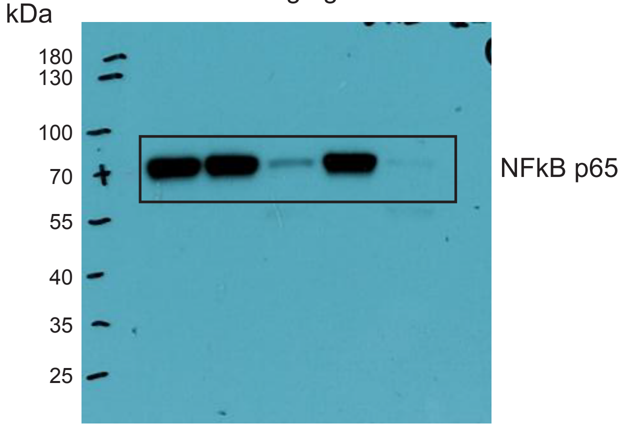


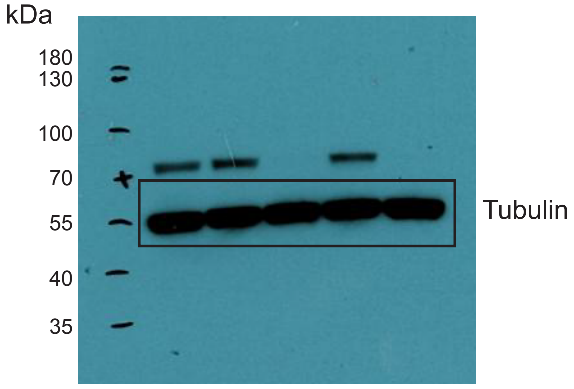


Extended Data Fig.6h

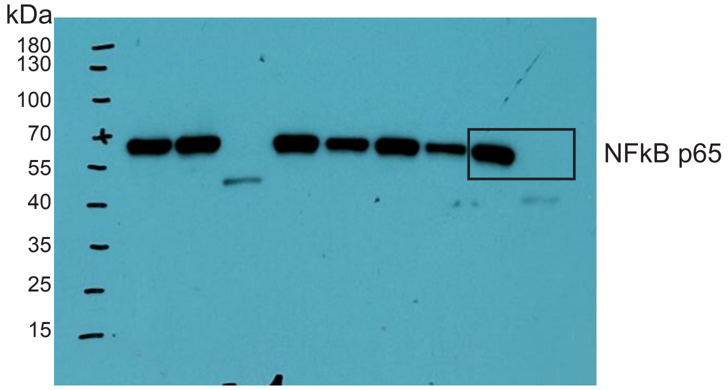


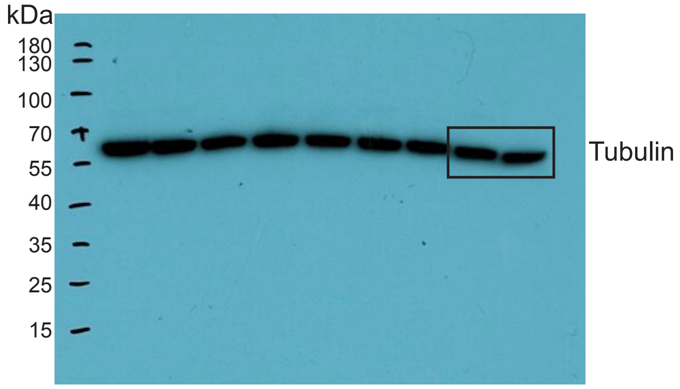


Extended Data Fig.6i

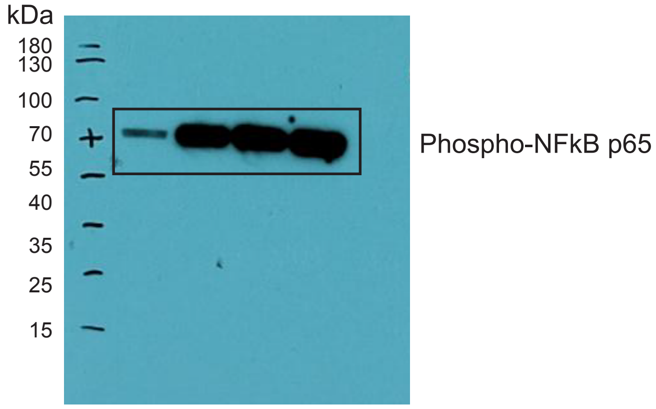


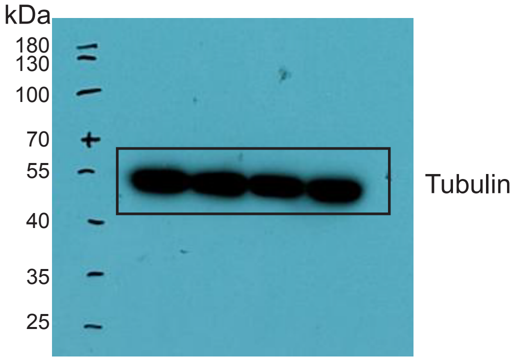

Supplement: Source Data Extended Data Fig. 6 [file EMS157168-supplement-Source_Data_Extended_Data_Fig__6.pdf]
