# Supplementary figures and images for: Targeting Menin disrupts the KMT2A/B and polycomb balance to paradoxically activate bivalent genes
Source: Nat Cell Biol. Author manuscript; Available in PMC 2023 Feb 17. (PMC7614190; doi:10.1038/s41556-022-01056-x)

Source Data: Western blot gels

Western blot images. Figure 2.

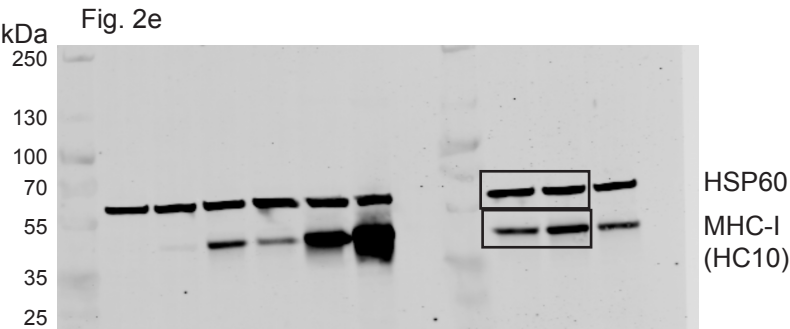

Supplement: Source Data Fig. 2 [file EMS157168-supplement-Source_Data_Fig__2.pdf]

Source Data: Western blot gels

Western blot images. Figure 3.

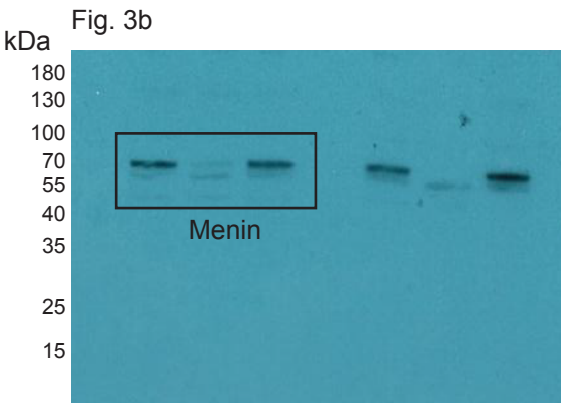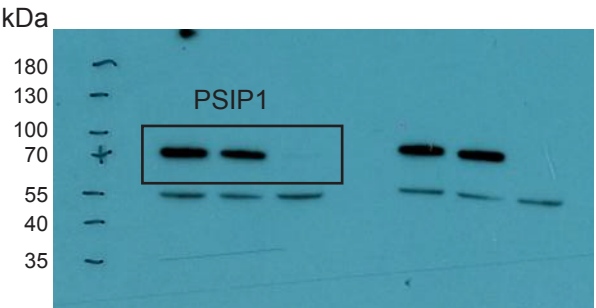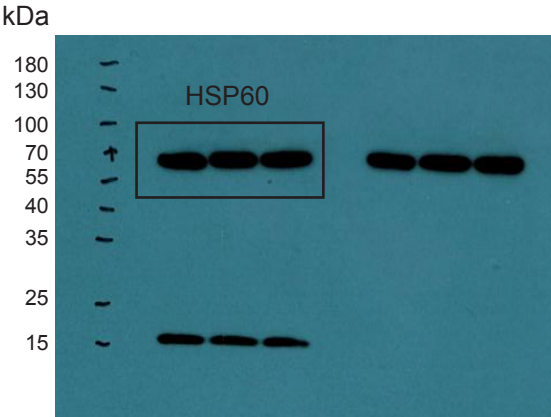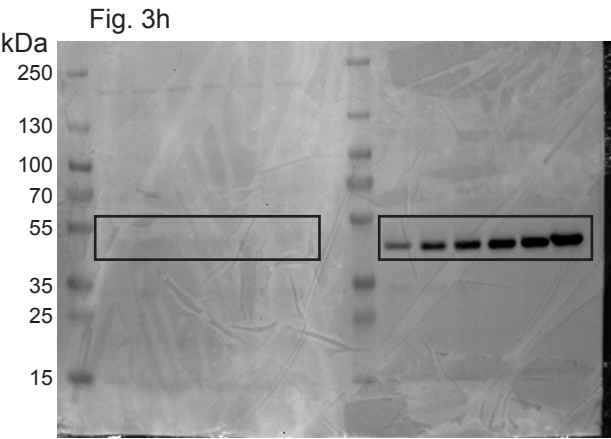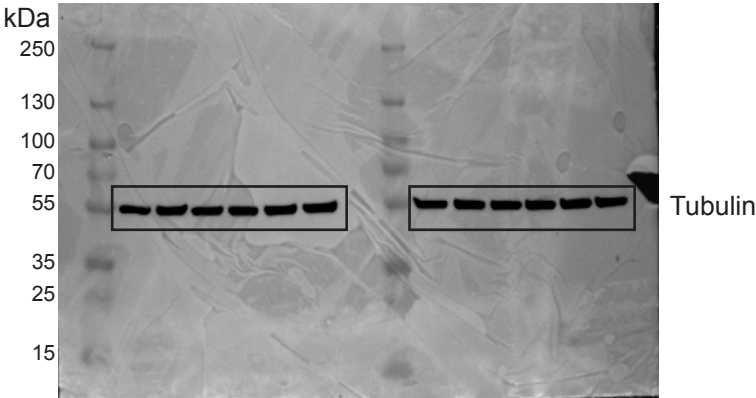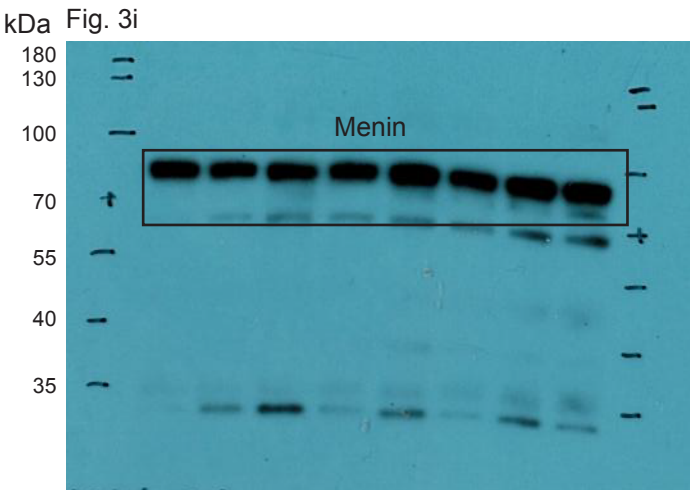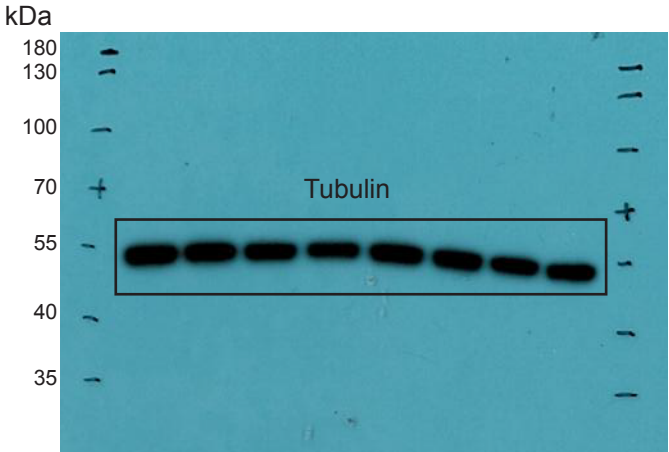

Supplement: Source Data Fig. 3 [file EMS157168-supplement-Source_Data_Fig__3.pdf]

Source Data: Western blot gels

Western blot images. Figure 5

Fig. 5b

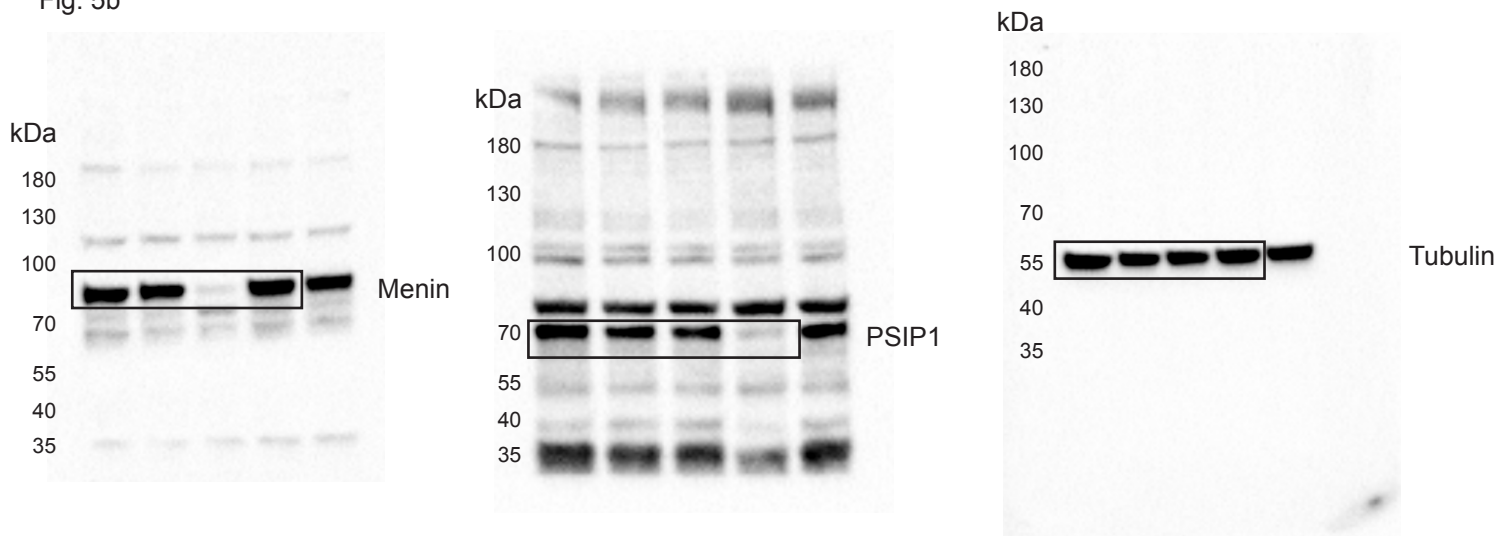

Supplement: Source Data Fig. 5 [file EMS157168-supplement-Source_Data_Fig__5.pdf]

Source Data: Western blot gels

Western blot images. Figure 8

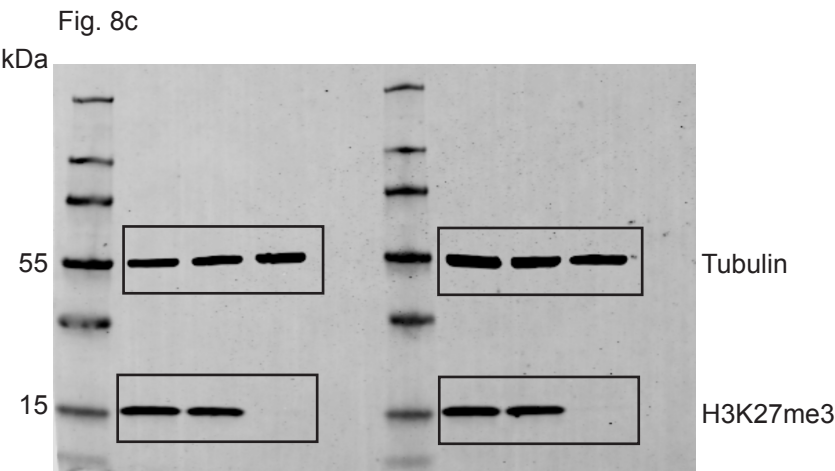

Supplement: Source Data Fig. 8 [file EMS157168-supplement-Source_Data_Fig__8.pdf]
