# Supplementary material for: Targeting Menin disrupts the KMT2A/B and polycomb balance to paradoxically activate bivalent genes
Source: Nat Cell Biol. Author manuscript; Available in PMC 2023 Feb 17. (PMC7614190; doi:10.1038/s41556-022-01056-x)
Supplement: Supplementary Figures 1-3. [file EMS157168-supplement-Supplementary_Figures_1_3_.pdf]

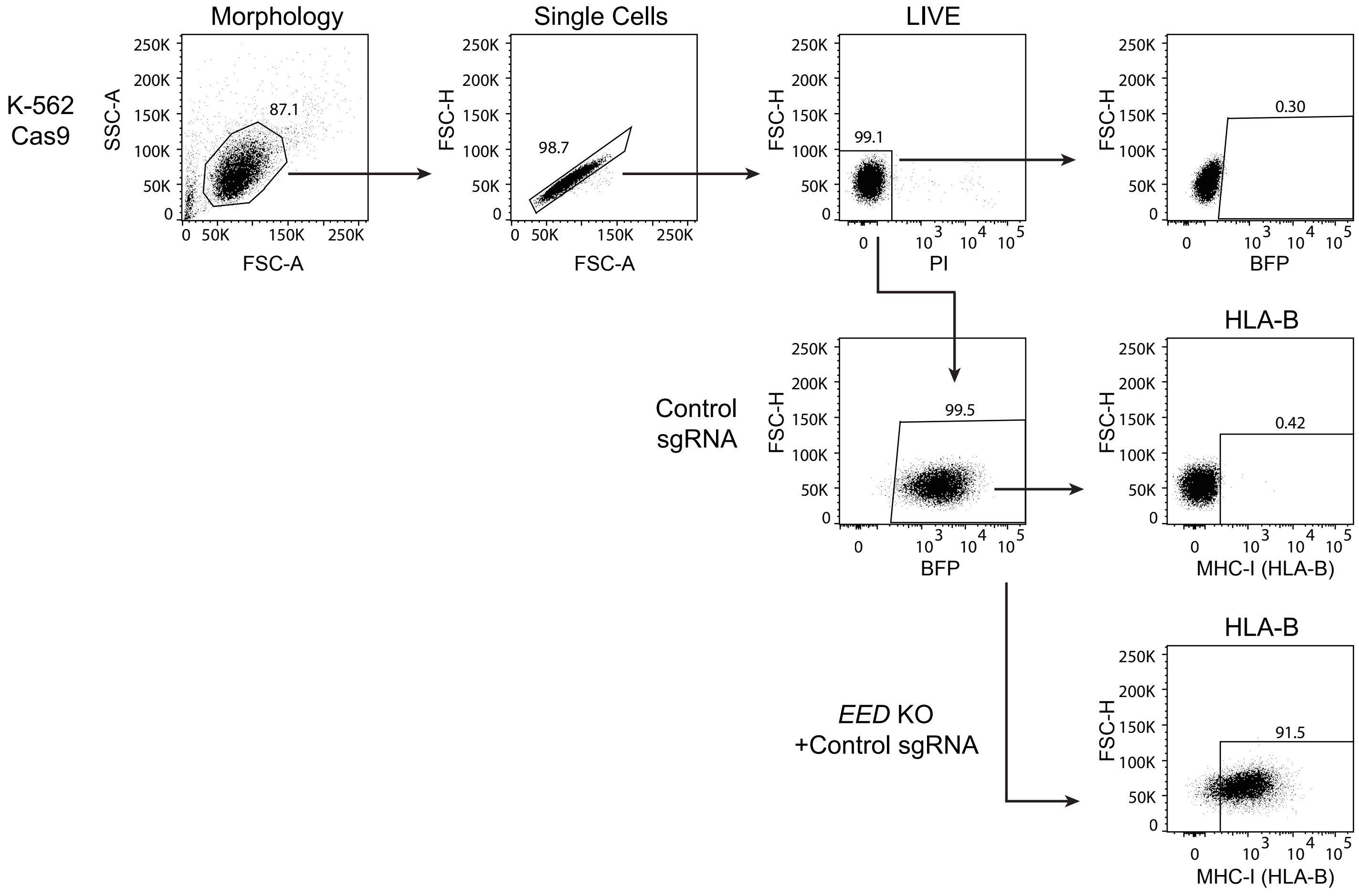


**Supplementary Figure 1:** Representative gating strategy of flow cytometry in K-562 Cas9 (top), K-562 Cas9 control sgRNA expressing (middle) and K-562 Cas9 *EED* KO control sgRNA expressing (bottom) cells, to assess cell surface MHC-I expression.

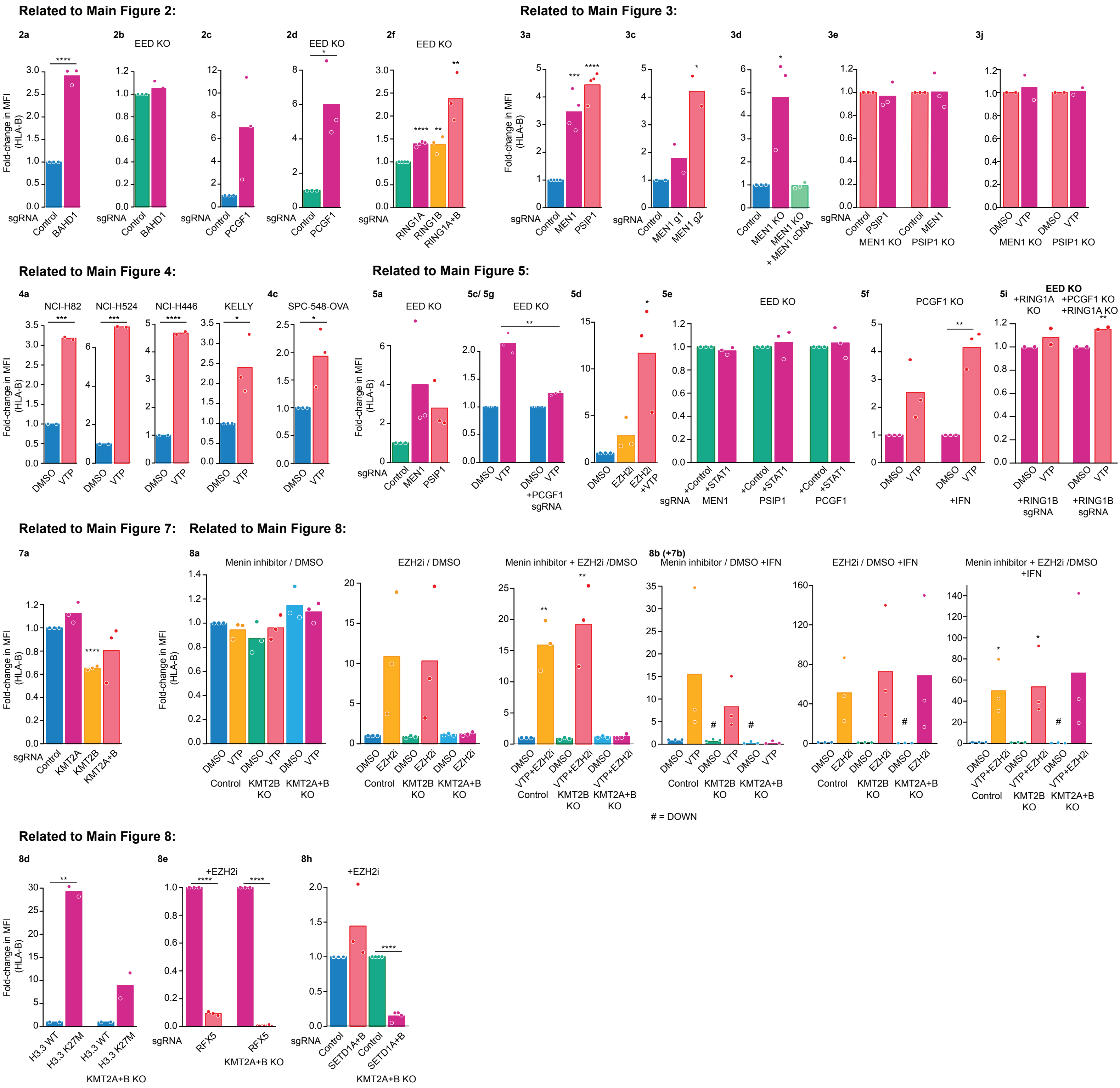


**Supplementary Figure 2: Related to Main Figures.**
Bar graphs depicting cell surface MHC-I as mean fold change in MFI from the indicated Main and Extended Data Figures. Points indicate independent replicates. Unpaired two-tailed t-test, significance is defined as p-value *<0.05, **<0.01, ***<0.001, ****<0.0001, non-significant data were not annotated.

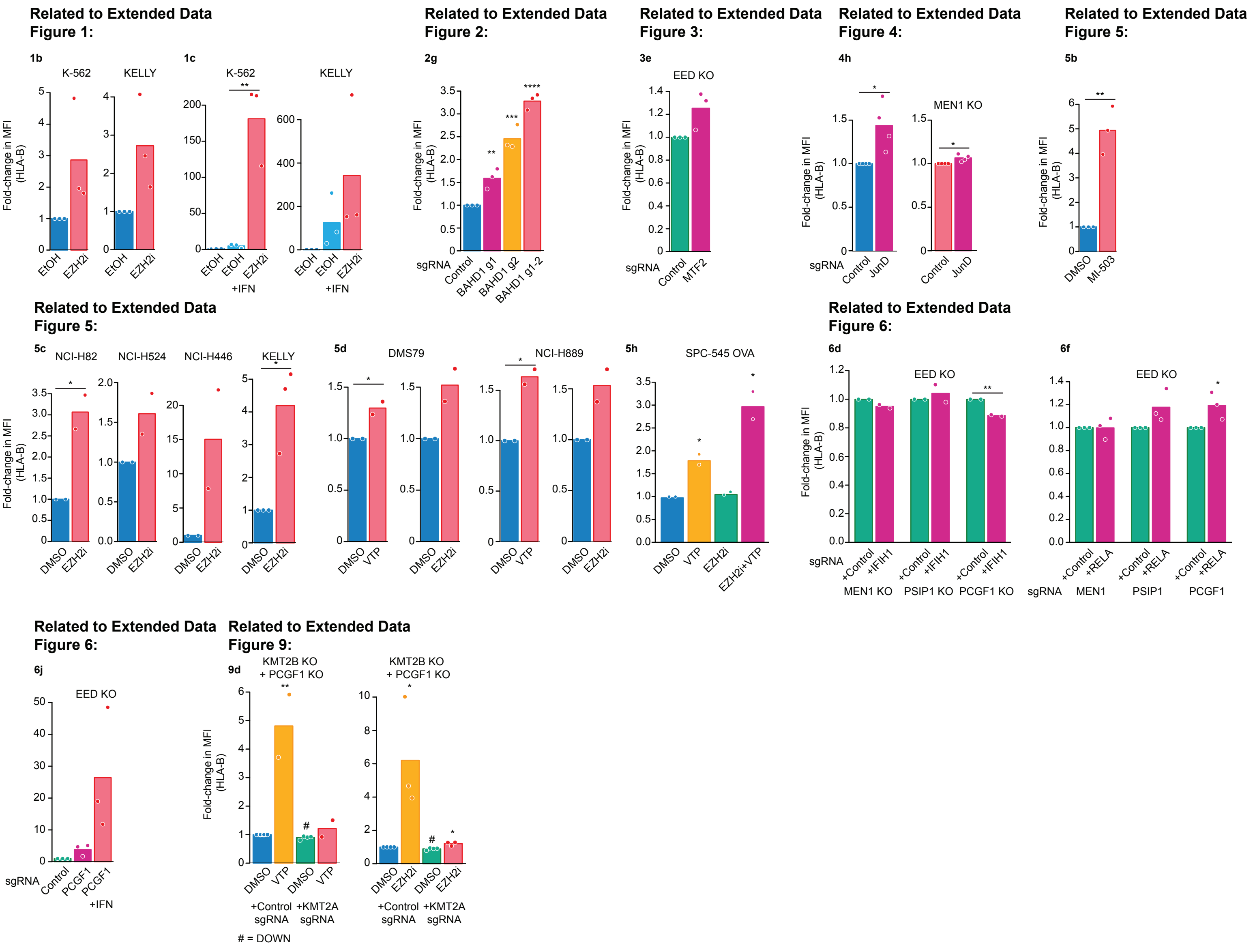


**Supplementary Figure 3: Related to Extended Data Figures.**
Bar graphs depicting cell surface MHC-I as mean fold change in MFI from the indicated Main and Extended Data Figures. Points indicate independent replicates. Unpaired two-tailed t-test, significance is defined as p-value *<0.05, **<0.01, ***<0.001, ****<0.0001, non-significant data were not annotated.
